# Using X‑ray Crystallography and 1,*n*‑ADEQUATE NMR to Revise the Structures of Highly Substituted Aromatic Natural Products: The Absolute Configuration of Formicamycin Congeners

**DOI:** 10.1021/acs.jnatprod.6c00145

**Published:** 2026-04-16

**Authors:** Edward S. Hems, Joseph A. Wright, Sergey A. Nepogodiev, Rebecca Devine, Corinne J. Arnold, David L. Hughes, Julia E. A. Mundy, Sandra Eltschkner, David M. Lawson, Matthew I. Hutchings, Barrie Wilkinson

**Affiliations:** † Molecular Microbiology, 15549 John Innes Centre, Norwich Research Park, Norwich NR4 7UH, United Kingdom; ‡ Centre for Microbial Interactions, Norwich Research Park, Norwich NR4 7UG, United Kingdom; § School of Chemistry, 6106 University of East Anglia, Norwich NR4 7TJ, United Kingdom; ∥ NMR Platform, 15549 John Innes Centre, Norwich Research Park, Norwich NR4 7UH, United Kingdom; ⊥ Biochemistry and Metabolism, 15549 John Innes Centre, Norwich Research Park, Norwich NR4 7UH, United Kingdom

## Abstract

Formicamycins and their biosynthetic precursors, the fasamycins, form part of the phenylnaphthacenoid family of polyketide natural products. A recent atroposelective total synthesis of formicamycin H brought into question our original stereochemical assignment of the axially chiral linkage between C-6 and C-7. To address this, we obtained an X-ray crystal structure for formicamycin H that unambiguously confirmed our original assignment as the *S*_a_ atropisomer. X-ray structures for multiple additional fasamycins and formicamycins confirmed that this is common to all congeners. However, these studies identified a compounded error made by us whereby several structures previously reported as *para*-methoxy were found to have *ortho*-methoxy groups on the hanging E-ring. To address this for congeners that did not crystallize or gave nondiffracting crystals, we turned to the surprisingly underutilized 1,*n*-ADEQUATE NMR experiment. In total, we generated X-ray structures for 15 phenylnaphthacenoid metabolites and by combining these results report the corrected structures for three formicamycins, six fasamycins, and three biosynthetic lactone intermediates, noting that several revised fasamycin structures now match previously reported naphthacemycins. Our results highlight the utility of 1,*n*-ADEQUATE experiments for regiochemical determination in polysubstituted aromatic molecules. Moreover, our investigations uncovered a potential deracemization step during biosynthesis of the formicamycin framework.

## Graphical abstract

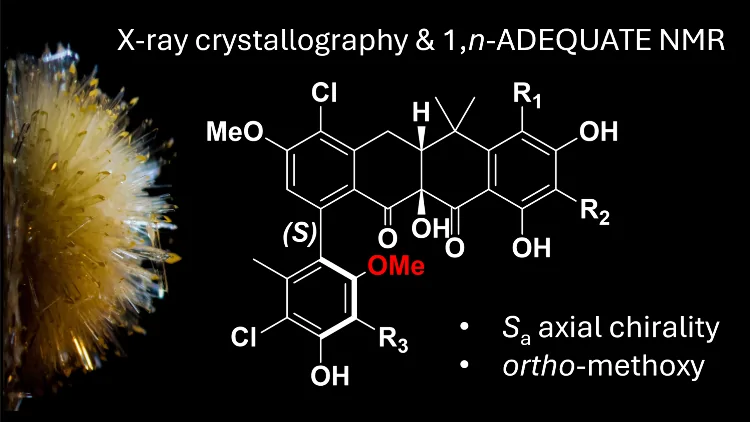

Fasamycins A and B were first reported after the heterologous expression of environmental DNA from soil.^1^ These polyketide specialized metabolites were found to have activity against both methicillin-resistant *Staphylococcus aureus* (MRSA) and vancomycin-resistant *Enterococcus faecalis* (VRE) by inhibiting the fatty acid condensing synthase enzyme FabF.^2^ Subsequently, we isolated further fasamycins in addition to the structurally related formicamycins from *Streptomyces formicae* KY5, which was isolated from the nests of Kenyan *Tetraponera penzigi* plant ants.^3,4^ Fasamycin congeners and their glycosylated derivatives have also been isolated from other actinomycetes and reported under multiple names including the fasamycins,^5^ streptovertimycins,^6–8^ naphthacemycins,^9–12^ and accramycins.^13–15^ These molecules collectively belong to the phenylnaphthacenoid family of specialized metabolites.^16^

In previous work, we delineated the biosynthetic pathway for formicamycins and showed that fasamycins are their biosynthetic precursors, which first undergo an oxidative Baeyer–Villiger ring expansion catalyzed by the flavin-dependent monooxygenase, ForX.^17^ The resultant lactone intermediate then undergoes reductive ring contraction catalyzed by the flavin-dependent reductase ForY to yield a formicamycin, which exhibits a significantly different three-dimensional structure when compared to the fasamycins. Derailment of this pathway leads to a racemic mixture of formicapyridine shunt metabolites.^18^ All of these phenylnaphthacenoids may be further halogenated and/or *O*-methylated to give a complex mixture of biosynthetic congeners.

During our campaign to understand the biosynthetic pathway of formicamycins, we have isolated and reported 39 different phenylnaphthacenoid structures from *S. formicae* KY5 and various knockout mutants,^4,17–19^ in addition to eight further glycosylated fasamycins after the heterologous expression^20^ of the formicamycin biosynthetic gene cluster (*for* BGC). To date, we have reported structures based primarily on NMR and mass spectrometry studies, and the absolute configurations of fasamycin E and formicamycin B were originally proposed by comparing calculated electronic circular dichroism (ECD) spectra with experimental ECD data.^4^ Because the carbon skeletons of subsequently reported congeners are all products of the same biosynthetic pathway, the relative stereochemistry of all congeners was assumed to be the same.

Recently, the atroposelective total synthesis of formicamycin H suggested that our original assignment of the natural product as having *S*_*a*_ axial chirality between C-6 and C-7 of the hanging E-ring may be incorrect.^21^ To investigate this, we successfully grew crystals of formicamycin H, which diffracted to 0.76 Å resolution on a synchrotron beamline, and unambiguously confirmed that natural formicamycin H isolated from *S. formicae* KY5 has *S*_*a*_ axial chirality, as originally proposed. In addition, we obtained X-ray crystallographic structures for several of our previously reported phenylnaphthacenoid congeners. These data confirmed the *S*_a_ axial chirality between C-6 and C-7 for all formicamcyins and fasamycins that crystallized, as well as the 10*R*,19*R* stereochemistry at the bridgehead position of formicamycins. However, X-ray crystallography unexpectedly showed that several structures that had previously been reported as having a *para*-methoxy group on the hanging E-ring instead have *ortho*-methoxy groups. This error was caused by misinterpretation of the original NOESY NMR spectrum of fasamycin C, which was compounded into the assignment of subsequent phenylnaphthacenoids.^4^ This result brought into question our assignment of several congeners that had not crystallized. To address this problem, we turned to 1,*n*-ADEQUATE NMR,^22^ and in combination with X-ray crystallography, we report the corrected structures of six fasamycins (four of which have been published under different names by other groups), three formicamycins, and three biosynthetic lactone intermediates in addition to the structure of one new lactone intermediate. To our surprise, the biosynthetic intermediate lactones crystallized as racemic mixtures, indicating that these molecules racemize in solution (an observation supported by DFT calculations also reported here) and that the reductive cyclization of these lactone intermediates may involve a previously cryptic deracemization step during the biosynthetic pathway.

## Results and Discussion

### Formicamycin H Has *S*_a_ Axial Chirality

A recent total synthesis of formicamycin H prompted us to re-examine our original assignment of the axial chirality about the C-6/C-7 bond of that compound, after suggesting that formicamycin H has *R*_a_ axial chirality.^21^ Preliminary experiments reported in this study tested the atroposelectivity of a Suzuki cross-coupling reaction, and resulting model compounds were shown to have *R*_a_ axial chirality by X-ray crystallography. The same Suzuki cross-coupling was then employed to synthesize formicamycin H, and the ^1^H NMR spectrum of the synthetic compound in methanol-*d*_4_ was found to match our original ^1^H NMR data for the natural product. Unfortunately, X-ray diffraction data were not reported for the synthetic material, and so while no claim was made about the absolute configuration of synthetic formicamycin H, it was strongly suggested that based on the available evidence that structural revision of the axial chirality of natural formicamycin H may be required. Additionally, a publication describing the structure of naphthacemycin A_9_ was incorrectly cited as a crystallographic example of a phenylnaphthacenoid natural product with *R*_a_ axial chirality.^21^ However, the published crystal structure of naphthacemycin A_9_ has a Flack parameter of 1.4(6) and as such no claim was made about the absolute configuration by the original authors.^9^

Our original assignment of phenylnaphthacenoid axial chirality was made by comparison of calculated electronic circular dichroism (ECD) spectra with experimental ECD spectra, which led us to suggest formicamycin H existed as the *S*_a_ atropisomer.^4^ Following our initial stereochemical proposal, several crystal structures have been reported for other phenylnaphthacenoid metabolites, which have sufficient quality to make claims about their absolute configurations. These include streptovertimycin W [Flack = 0.00(6)],^8^ streptovertimycin Z_1_ [Flack = −0.008(12)],^8^ and accramycin A [Flack = 0.03(7)]:^7^ thus, these three compounds were all unambiguously assigned as the *S*_a_ atropisomers. Moreover, a recent publication has shown that FasU, the enzyme that catalyzes the formation of the biphenyl framework during fasamycin biosynthesis (biosynthetic precursors of the formicamycins), does so with strict *S*_a_ stereospecificity.^23^

We first ran ^1^H and ^13^C NMR of natural formicamycin H (**1**) in CDCl_3_, and this was found to match the NMR spectral data for synthetic formicamycin H, which was reported in the same solvent (Supporting Information, Tables S1 and S2 and Figures S1 and S2).^21^ This supports the published experiment, which showed that the ^1^H NMR spectra of synthetic formicamycin H in methanol-*d*_4_ matched with our original assignment of natural formicamycin (**1**) in the same solvent.^4,21^ Next, we successfully crystallized natural formicamycin H (**1**) by slow evaporation from a 2:1 mixture of dichloromethane/cyclohexane. The resulting crystals diffracted on a synchrotron beamline to 0.76 Å resolution, which allowed us to unambiguously assign natural formicamycin H as the *S*_a_ atropisomer [Flack = 0.032(7)], as originally proposed (Figure 1).

**Figure 1:**
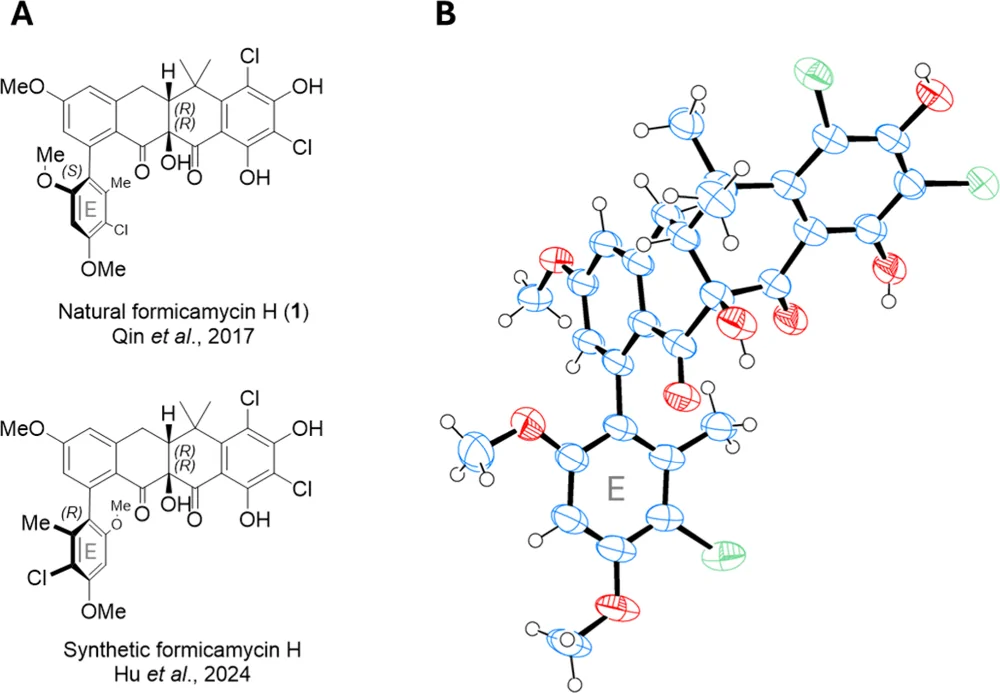
(A) Proposed atropisomers of natural formicamycin H (**1**) isolated from *S. formicae* KY5 and synthetic formicamycin H. (B) ORTEP representation of the crystal structure of natural formicamycin H that was unambiguously assigned as the *S*_a_ atropisomer. Ellipsoids are given at the 50% probability level.

### Revising Published Formicamycin and Fasamycin Structures

We next re-examined the structures of additional formicamycin and fasamycin congeners produced by *S. formicae* KY5 to demonstrate that, as we proposed, they all exhibit the same *S*_a_ axial chirality about the C-6/C-7 bond. By growing crystals under similar conditions to those used for formicamycin H (**1**), we obtained crystal structures of six formicamycins (Table 1), four fasamycins (Table 2), two biosynthetic lactone intermediates (Table 3), and two formicapyridines (Table 4). All formicamycins and fasamycins were isolated as single enantiomers and crystallized in Sohncke space groups with Flack parameters which allowed the determination of their absolute configurations.^24^ All were unambiguously solved as the *S*_a_ atropisomer, which is in keeping with all previously published high-quality phenylnaphthacenoid crystal structures and the recent study showing the strict *S*_a_ atropstereospecificity of the biosynthetic enzyme FasU (*vide supra*). Based on our crystallography data (Tables 1 and 2) and complementary crystallography and enzymology data from other groups, we suggest that all formicamycin and fasamycin congeners have *S*_a_ axial chirality.

**Table 1:**
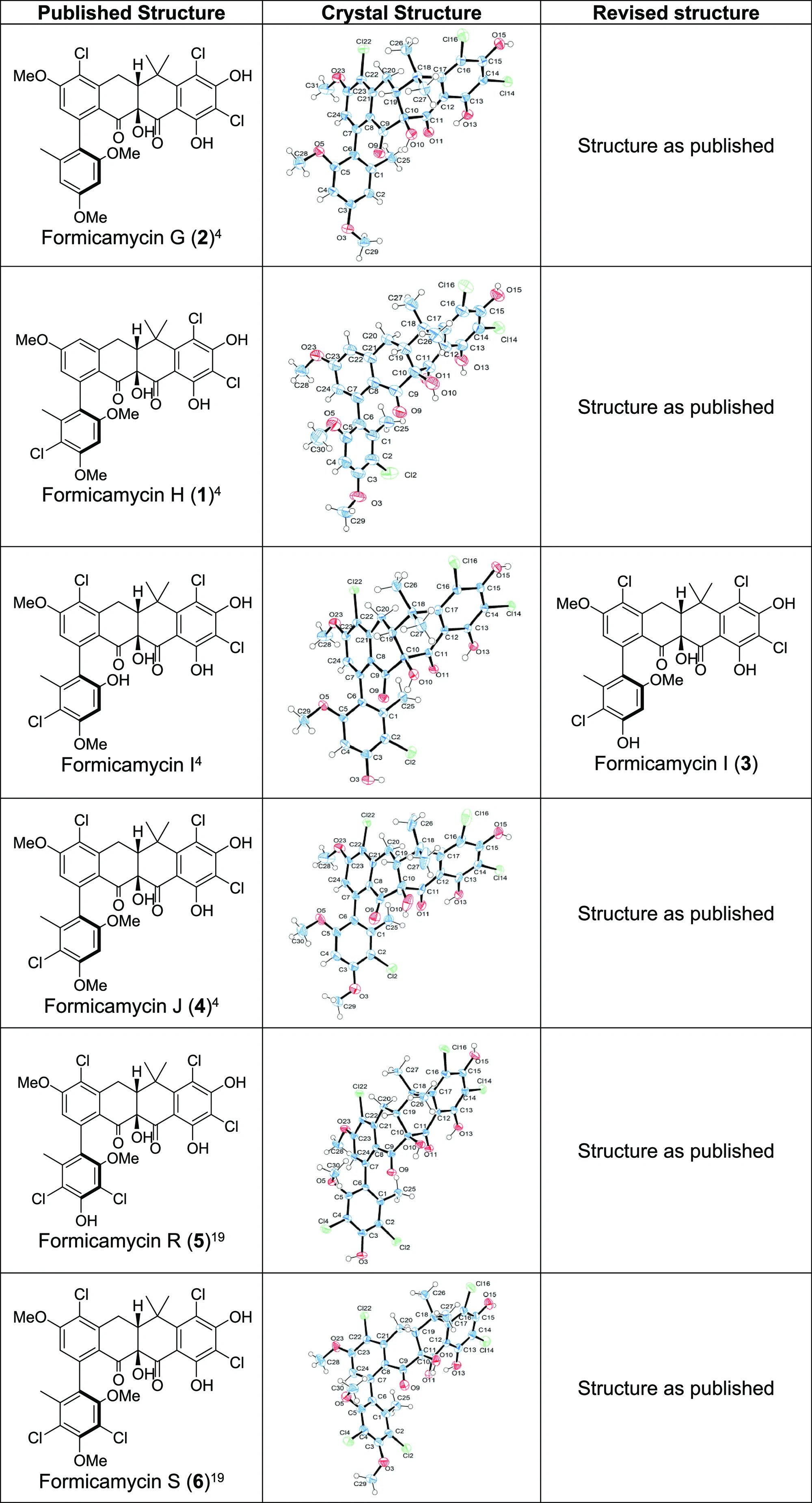
Comparison of Previously Published Formicamycin Metabolites from Our Groups with the ORTEP Representation of Their Crystallographic Structures^a^

a Ellipsoids are given at the 50% probability level. Where necessary, a revised structure is given.

**Table 2:**
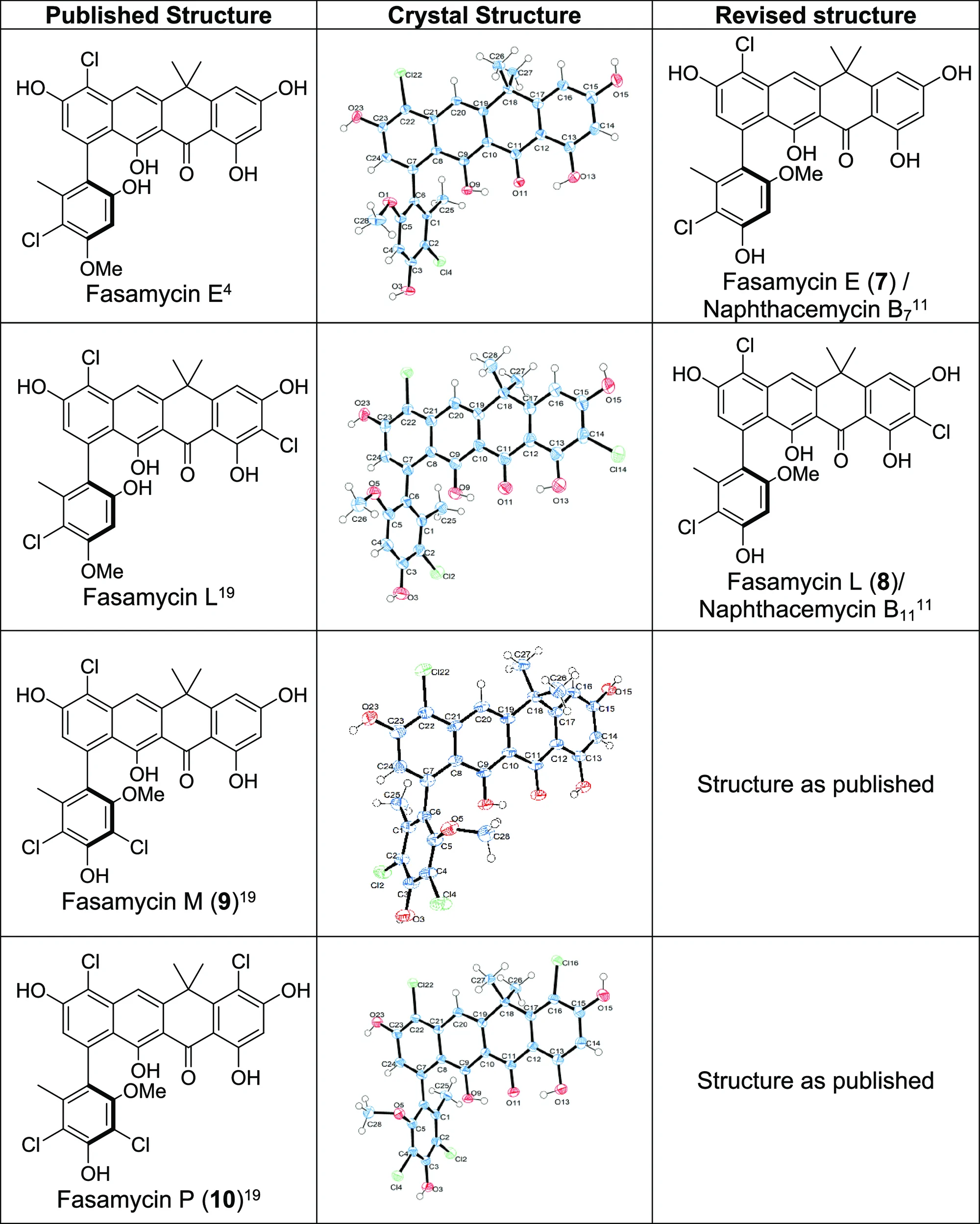
Comparison of Previously Published Fasamycin Metabolites from Our Groups with the ORTEP Representation of Their Crystallographic Structures^a^

a Ellipsoids are given at the 50% probability level. Where necessary, a revised structure is given.

**Table 3:**
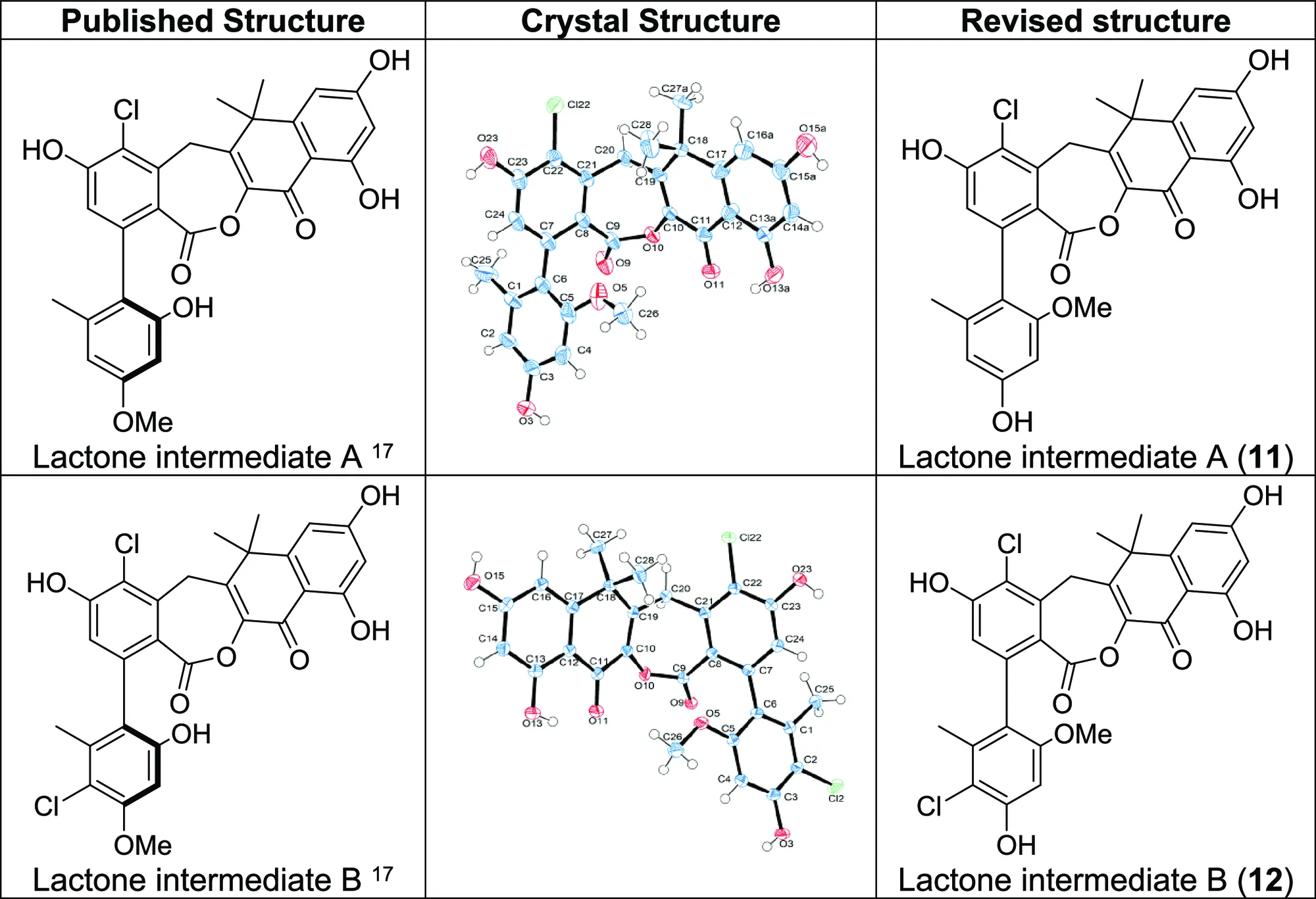
Comparison of Previously Published Biosynthetic Lactone Intermediates from Our Groups with the ORTEP Representation of Their Crystallographic Structures^a^

a Ellipsoids are given at the 50% probability level. Revised structures are given.

**Table 4:**
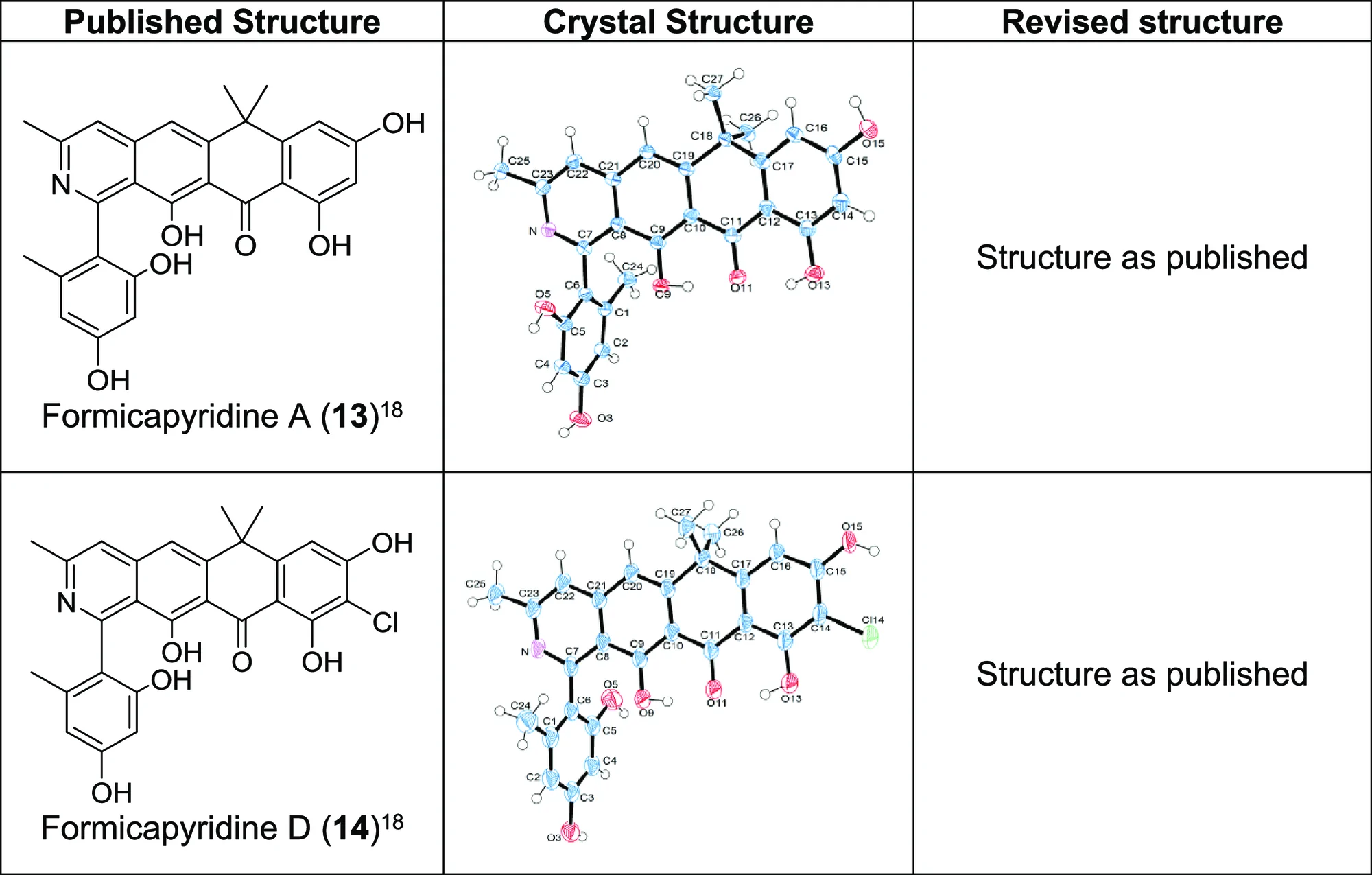
Comparison of Previously Published Formicapyridine Shunt Products from Our Groups with the ORTEP Representation of Their Crystallographic Structures^a^

a Ellipsoids are given at the 50% probability level. Where necessary a revised structure is given.

Although our crystallography data confirmed the *S*_a_ axial chirality of formicamycins and fasamycins, they also highlighted a compounded error in some of our previously published structures. Formicamycin I (**3**) was found to have *ortho*-methoxy groups on the hanging E-ring whereas the structures had originally been reported as *para*-methoxy (Table 1). Furthermore, fasamycin E (**7**) and fasamycin L (**8**) were both found to have *ortho*-methoxy groups whereas the structures had previously been reported as *para*-methoxy (Table 2). The crystallographic structure of fasamycin E (**7**) matched the structure of naphthacemycin B_7_ reported by Huo et al.,^11^ and as such, we reran ^1^H and ^13^C NMR spectra in acetone-*d*_6_ for comparison with published spectra (Supporting Information, Tables S9 and S10 and Figures S5 and S6). We noticed splitting of peaks in both our ^1^H and ^13^C NMR spectra, but the midpoint of each signal was again in good agreement; thus, fasamycin E (**7**) and naphthacemycin B_7_ are the same compound. Similarly, the crystallographic structure of fasasmycin L (**8**) matched the published structure of naphthacemycin B_11_.^11^ This time, ^1^H and ^13^C NMR spectra of fasamycin L (**8**) were recorded in CDCl_3_ and were in good agreement with those published by Huo et al.^11^ (Supporting Information, Tables S11 and S12 and Figures S7 and S8); thus, fasamycin L (**8**) and naphthacemycin B_11_ are the same compound.

Next, we examined the biosynthetic lactone intermediates (Table 3).^17^ These compounds are biosynthetic intermediates between fasamycins and formicamycins and are produced by the oxidative ring expansion of fasamycins catalyzed by the Baeyer–Vanilliger monooxygenase ForX. These lactones then undergo a reductive ring contraction catalyzed by ForY to give formicamycins. We expected the crystal structures of these intermediates to exhibit the same strict *S*_a_ axial chirality as their fasamycin precursors and formicamycin end products and were surprised to see that both lactones A (**11**) and B (**12**) crystallized in non-Sohncke space groups indicating a racemic mixture, prompting us to revisit our previous DFT-calculated barriers to rotation (*vide infra*). We also report in this work a novel intermediate isolated from the *S. formicae* KY5 Δ*forJ*Δ*forY* mutant (lactone intermediate F), which also crystallized in a non-Sohncke space group (*vide infra*) (Figure 5). Here again, X-ray crystallography highlighted an error in our previously published structures, with lactones A (**11**) and B (**12**) having *ortho*- rather than *para*-methoxy groups on the hanging E-ring.

Finally, formicapyridines are known shunt metabolites formed by derailment of cyclization stage of fasamycin biosynthesis and are isolated as a mixture of atropisomers.^18^ As such, both formicapyridine A (**13**) and D (**14**) crystallized in non-Sohncke space groups with *P*2_1_/*n* symmetry (Table 4). We note that we previously published structures for formicapyridines B, E, and H with *para*-methoxy groups on the hanging E-ring.^18^ Unfortunately, we no longer have these compounds to hand, but considering the structural revisions reported in this paper, we would suggest these structures may require future revision.

### Using 1,*n*-ADEQUATE NMR to Determine the Regiochemistry of *O*-Methoxy Groups on Highly Substituted Aromatic Rings

Following the structural revisions of formicamycin I (**3**), fasamycins E (**7**) and L (**8**), and biosynthetic lactone intermediates A (**11**) and B (**12**) by X-ray crystallography, we believed it prudent to check the assignment of the remaining phenylnaphthacenoids previously reported by our laboratories with a single methoxy group on the hanging E-ring, which had failed to crystallize despite extensive experimentation. Specifically, this meant re-examining formicamycins B (**15**)^4^ and D (**16**);^4^ fasamycins C (**17**),^4^ D (**18**),^4^ F (**19**),^18^ N (**20**),^19^ and Q (**21**);^19^ and lactone intermediate D (**22**).^17^ We regret that we no longer have any other formicapyridines to check. The original structural assignments of these phenylnaphthacenoids were based on the assignment of fasamycin C, which was determined to have a *para*-methoxy substitution on the E-ring by using NOESY cross-peaks between the methoxy group and H-2 and H-4.^4^ On re-examining and repeating this initial experiment, protons H-2 and H-4 have the same chemical shift and appear as a single peak in the ^1^H NMR spectrum (methanol-*d*_4_ at 298 K). As such, it is not possible to distinguish between the *ortho-* and *para-*methoxy regioisomers of fasamycin C by Overhauser effect spectroscopy under these conditions (Supporting Information, Figure S18). Unfortunately, this was inappropriately assigned as having *para*-methoxy regiochemistry without further investigation, and this incorrect assignment was carried forward for many related phenylnaphthacenoids reported by our groups.

To re-examine the regiochemistry of the methoxy group on the hanging E-ring for the phenylnaphthacenoids that would not crystallize, we used 1,*n*-ADEQUATE NMR, which has previously been described for characterizing highly substituted aromatic compounds.^25^ The 1,*n*-ADEQUATE experiment allows us to view pseudo ^4^ *J*_CH_ correlations (^1^ *J*_CH_ + ^3^ *J*_CC_) that could be used to readily determine the regiochemistry of the methoxy group on the phenylnaphthacenoid E-ring. To test this, we first ran 1,*n*-ADEQUATE NMR with a delay for the ^*n*^ *J*_CC_ coupling of 7 Hz for fasamycin E/naphthacemycin B_7_ (**7**), with the structure having already been revised by X-ray crystallography (Figure 2).

**Figure 2:**
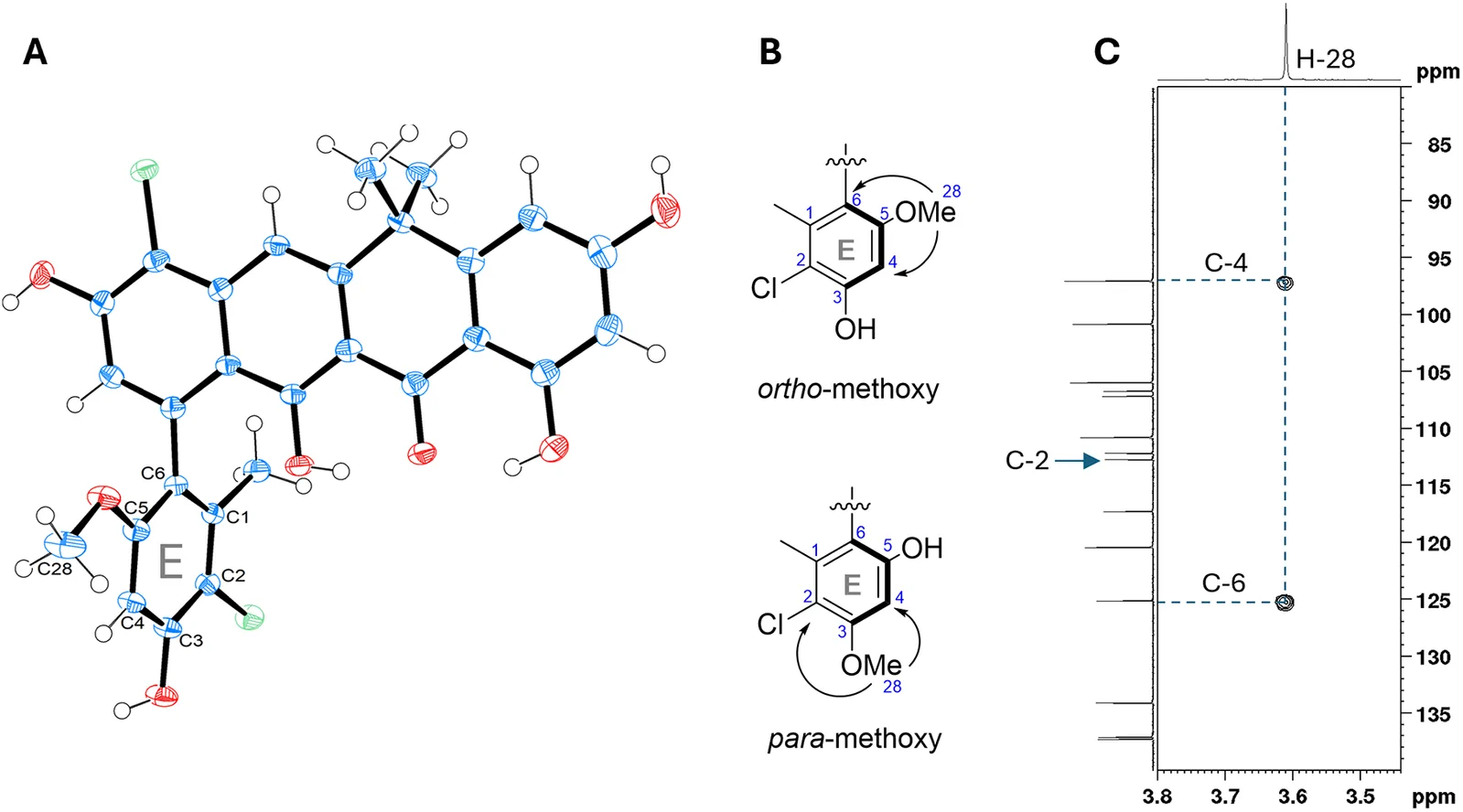
(A) ORTEP representation of the X-ray crystal structure for fasamycin E/naphthacemycin B_7_ (**7**). Ellipsoids were set to 50% probability. (B) Expected pseudo ^4^ *J*_CH_ correlations in the 1,*n*-ADEQUATE spectrum for *ortho*-methoxy and *para*-methoxy regioisomers of fasamycin E. (C) Expansion of the 1,*n*-ADEQUATE spectrum for formicamycin E/naphthacemycin B_7_ (**7**) with a delay for ^*n*^ *J*_CC_ coupling of 7 Hz. ^4^ *J*_CH_ correlations are visible from the methoxy protons (H-28) to C-4 and C-6, consistent with the *ortho-* regiochemistry observed in the X-ray crystal structure.

Here 1,*n*-ADEQUATE NMR data for fasamycin E (**7**) showed pseudo ^4^ *J*_CH_ correlations from the methoxy protons (H-28, δ_H_ 3.61) to C-4 (δ_C_ 98.4) and C-6 (δ_C_ 126.5) while no cross-peak was seen from H-28 to C-2 (δ_C_ 114.0) (Supporting Information, Figure S20). Where there was sufficient material, we also ran 1,*n*-ADEQUATE NMR for other phenylnaphthacenoids, which had crystallized with a single *ortho*-methoxy group on the hanging E-ring: formicamycin I (**3**) (Supporting Information, Figure S27), formicamycin R (**5**) (Supporting Information, Figure S28), fasamycin L (**8**) (Supporting Information, Figure S21), fasamycin M (**9**) (Supporting Information, Figure S22), lactone intermediate A (**11**) (Supporting Information, Figure S29), and lactone intermediate B (**12**) (Supporting Information, Figure S30). All showed pseudo ^4^ *J*_CH_ correlations from the methoxy protons (H-28) with C-4 and C-6, consistent with their crystal structures.

Next, we turned our attention to the phenylnaphthacenoids with a single methoxy group on the hanging E-ring, which we had not been able to crystallize. We were successful in obtaining usable 1,*n*-ADEQUATE data in methanol-*d*_4_ at concentrations above 10 mM using a 5 mm Shigemi tube on a Bruker Neo 600 MHz spectrometer equipped with 5 mm TCI CryoProbe. The 1,*n*-ADEQUATE NMR spectrum for fasamycin C (**17**) (Supporting Information, Figure S19) showed pseudo ^4^ *J*_CH_ correlations from the methoxy protons H-28 (δ_H_ 3.60) to C-4 (δ_C_ 97.4) and C-6 (δ_C_ 125.9) indicative of the *ortho*-methoxy regioisomer (Figure 3). As before, we checked the literature to see if these revised structures had been reported by other groups and found that the revised structure of fasamycin C matched the structure of naphthacemycin B_2_ first reported by Fukumoto et al.^9^ As such, we ran the ^1^H and ^13^C NMR spectra of fasamycin C (**17**) in acetone-*d*_6_ for comparison with published spectra (Supporting Information, Tables S3 and S4). We assigned the signals corresponding to C-9 and C-23 the opposite way around to Fukumoto et al., but allowing for this, the 1D NMR spectra are in good agreement. The ^1^H and ^13^C NMR spectra of naphthacemycin B_2_ have also been reported in acetone-*d*_6_ by Huo et al.^11^ so we also compared our ^1^H and ^13^C NMR spectra of fasamycin C in acetone-*d*_6_ with their published spectra (Supporting Information, Tables S5 and S6 and Figures S4 and S5), which were also in good agreement; thus, fasamycin C (**17**) and naphthacemycin B_2_ are the same compound.

**Figure 3:**
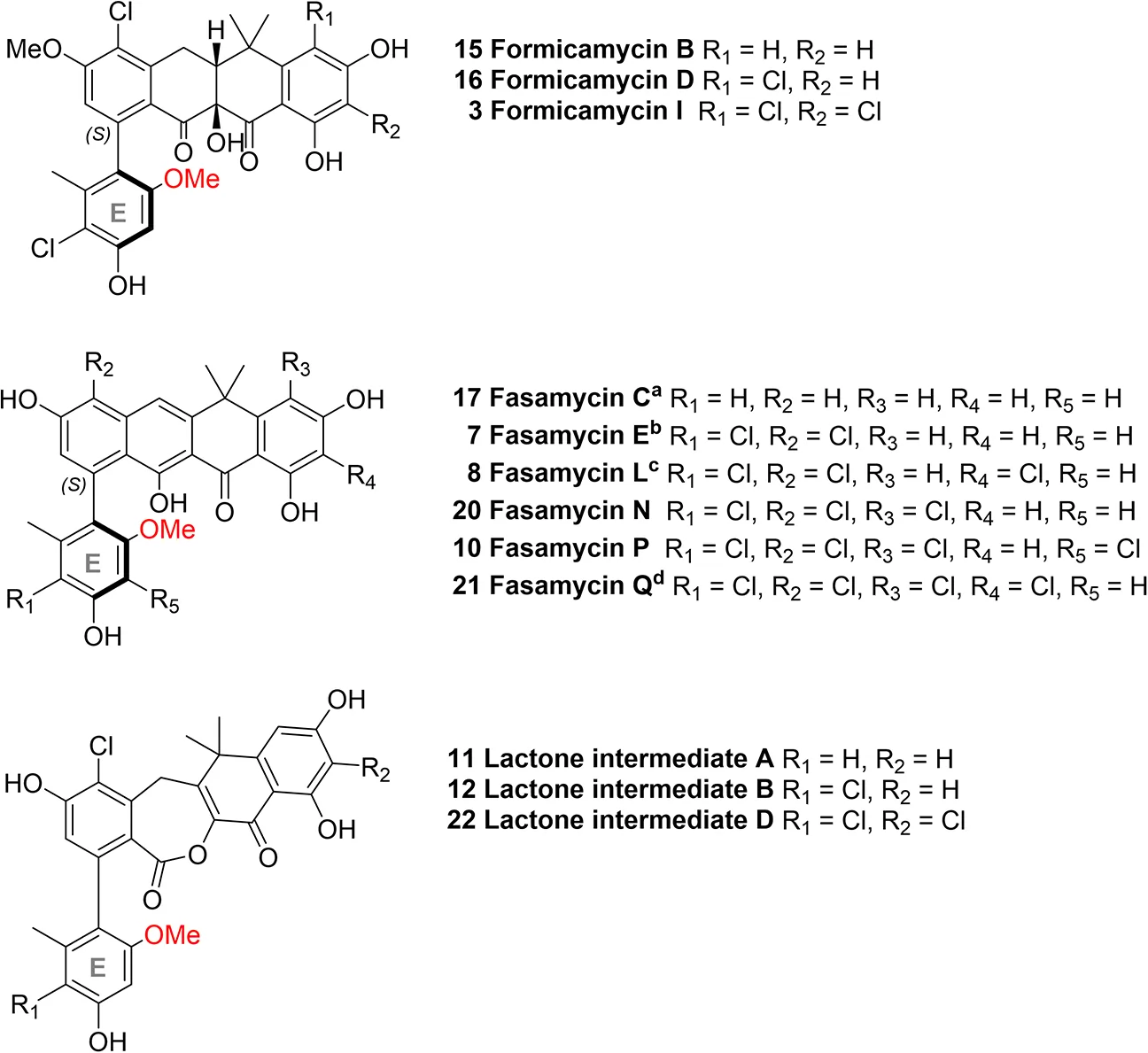
Revised structures of phenylnaphthacenoids previously incorrectly reported by our groups as *para* methoxylated on the E-ring. ^a^Fasamyin C (**17**) is the same molecule as naphthacemycin B_2_; ^b^fasamycin E (**7**) is the same molecule as naphthacemycin B_7_; ^c^fasamycin L (**8**) is the same molecule as naphthacemycin B_11_; and ^d^fasamycin Q (**21**) is the same molecule as naphthacemycin B_12_.

The 1,*n*-ADEQUATE NMR spectra for fasamycin N (**20**) (Supporting Information, Figure S23) showed pseudo ^4^ *J*_CH_ correlations from the methoxy protons H-28 (δ_H_ 3.46) to C-4 (δ_C_ 114.6) and C-6 (δ_C_ 131.8) indicative of the *ortho*-methoxy regioisomer (Figure 3). The revised structure does not match any published phenylnaphthacenoids.

The 1,*n*-ADEQUATE NMR spectra for fasamycin Q (**21**) (Supporting Information, Figure S24) showed pseudo ^4^ *J*_CH_ correlations from the methoxy protons H-28 (δ_H_ 3.59) to C-4 (δ_C_ 98.4) and C-6 (δ_C_ 126.4) indicative of the *ortho*-methoxy regioisomer (Figure 3). A search of the literature showed that the revised structure for fasamycin Q matched that of naphthacemycin B_12_. The ^1^H and ^13^C NMR of fasamycin Q were recorded in CDCl_3_ for comparison with those published by Huo et al.^11^ in the same solvent (Supporting Information, Tables S13 and S14 and Figures S9 and S10) and found to be in good agreement; thus, fasamycin Q (**21**) and naphthacemycin B_12_ are the same compound.

Moving on to formicamycins, we recorded the 1,*n*-ADEQUATE NMR spectra for formicamycin B (**15**) (Supporting Information, Figure S25), which showed pseudo ^4^ *J*_CH_ correlations from the methoxy protons H-28 (δ_H_ 3.60) to C-4 (δ_C_ 98.5) and C-6 (δ_C_ 124.1) indicative of the *ortho*-methoxy regioisomer (Figure 3). The revised structure does not match any published phenylnaphthacenoids.

The 1,*n*-ADEQUATE NMR spectrum for formicamycin D (**16**) (Supporting Information, Figure S26) showed pseudo ^4^ *J*_CH_ correlations from the methoxy protons H-28 (δ_H_ 3.61) to C-4 (δ_C_ 98.5) and C-6 (δ_C_ 124.1) indicative of the *ortho*-methoxy regioisomer (Figure 3). The revised structure does not match any published phenylnaphthacenoids.

The final compound for which we were able to obtain 1,*n*-ADEQUATE NMR was lactone intermediate D (**22**) (Supporting Information, Figure S31), which showed pseudo ^4^ *J*_CH_ correlations from the methoxy protons H-28 (δ_H_ 3.41) to C-4 (δ_C_ 98.4) and C-6 (δ_C_ 122.4) indicative of the *ortho*-methoxy regioisomer (Figure 3). The revised structure does not match any published phenylnaphthacenoids.

We do not have any remaining fasamycin D (**18**)^4^ with which to run 1,*n*-ADEQUTE NMR, so we decided to check the literature for the *ortho*-methoxy analogue and found that this has been reported as naphthacemycin B_4_ by Fukumoto et al.^9^ However, the original NMR spectrum was recorded in acetone-*d*_6_. As such, a comparison was made between our published NMR for fasamycin D (**18**)^4^ in methanol-*d*_4_ to the later NMR for napthacemycin B_4_ reported by Yuan et al.^5^ in the same solvent (Supporting Information Tables S7 and S8). These spectra are in good agreement, and considering all other examples in this paper, we suggest that fasamycin D (**18**) and naphthacemycin B_4_ are most likely to be the same compound.

We no longer have any stock of fasamycin F^4^, and no *ortho*-methoxy analogue has been reported in the literature. However, given all other examples in this paper, the regiochemistry of the methoxy group on the hanging E-ring may require revision from *para-*methoxy to *ortho*-methoxy.

### Determining the Barrier to Rotation about the C-6/C-7 Bond Using DFT Calculations

We previously reported the calculated barrier to rotation for fasamycin C using DFT calculations as Δ*E* = 156.1 kJ mol^–1^.^4^ We now know that these calculations were performed by following a lowest energy conformation search using the incorrect structure. With unambiguous X-ray crystallography structures in hand, we decided to repeat these calculations for the rotational energy barrier of the C-6/C-7 bond. Starting with fasamycin E (**7**), a gas-phase geometry optimization followed by a dihedral angle sweep in two directions was performed. From this transition state, energies on the inversion points were calculated, and the forward and backward barriers were 182 and 159 kJ mol^–1^, respectively. LaPlante’s qualitative guide to classification of atropisomers groups compounds with a rotational barrier greater than ∼125.5 kJ mol^–1^ as Class 3 atropisomers, meaning they should exhibit little to no axial rotation, with the frequency of rotation on the order of years.^26^ As such, fasamycin E (**7**) should be stable as the *S*_a_ atropisomer. Similarly, for formicamycin I (**3**), the calculated forward and backward barriers were 111 and 154 kJ mol^–1^, respectively, meaning **3** should likely be stable as the *S*_a_ atropisomer. The lower barrier is likely to be close to the Class 3 barrier, allowing for the typical variance of experimental and DFT energy values.

Next, we considered formicapyridines, which are shunt metabolites isolated as a racemic mix of atropisomers.^18^ Utilizing the crystal structure of formicapyridine A (**13**), it was interesting to see that the calculated forward and backward barriers to rotation were 150 and 130 kJ mol^–1^, respectively, meaning that there is no free rotation. This supports our original suggestion that racemization occurs at the point of derailment from the biosynthetic pathway rather than by free rotation about the C-6/C-7 bond.^18^

Finally, we were very interested in calculating the barrier to rotation for a biosynthetic lactone intermediate after these unexpectedly crystallized as a racemic mixture. On initial inspection of the crystal structures of lactone intermediates, we noticed that the carbonyl group on C-9 is twisted away from the hanging E-ring when compared with the hydroxy group on fasamycins or the carbonyl group on formicamycins at this position (Figure 4). We considered whether this could reduce the steric barrier to rotation and lead to racemization in solution. Starting from the crystal structure of lactone intermediate B (**12**), we calculated that the forward and backward barriers to rotation were 94 and 176 kJ mol^–1^, respectively. The forward rotation is now clearly within the LaPlante Class 2 range where slow rotation is viable, which likely accounts for the loss of chirality seen here.

**Figure 4:**
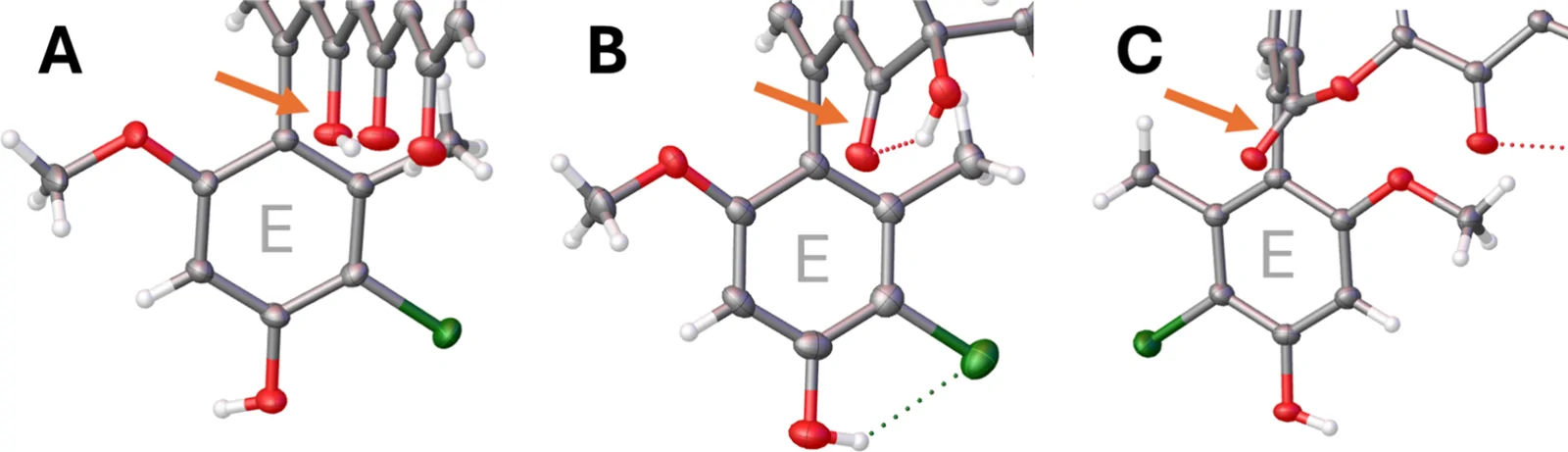
Hydroxy or carbonyl group on C-9 sterically blocks rotation about the C-6/C-7 bond in fasamycins and formicamycins, respectively. (A) X-ray crystal structure showing the C-9 hydroxy group on fasamycin E (**7**). (B) X-ray crystal structure showing the C-9 carbonyl group on formicamycin I (**3**). (C) X-ray crystal structure showing the C-9 carbonyl group on lactone intermediate B (**12**), which is twisted away from the hanging E-ring when compared with **3** or **7**.

### Isolation and Characterization of New Biosynthetic Lactone Intermediate F

ForJ is a MarR family regulator that represses expression of formicamycin biosynthesis genes in *S. formicae* KY5. We have previously shown that deleting *forJ* in *S. formicae* KY5 increases production of formicamycins.^19^ Therefore, during a recent attempt to increase production titers of lactone intermediates produced by *S. formicae* KY5 Δ*forY*, we engineered the double knockout mutant *S. formicae* KY5 Δ*forJ*Δ*forY*. This strain was grown on MS agar for 10 days and extracted with ethyl acetate for comparison with the previously isolated yields of biosynthetic lactones from *S. formicae* KY5 Δ*forY* (Table 5).

**Table 5:** Comparison of Isolated Biosynthetic Intermediate Lactone Yields between *S. formicae* KY5 Δ*forY* and *S. formicae* KY5 Δ*forJ*Δ*forY*

lactone intermediate	molecular mass	isolated from *S. formicae* KY5 Δfor*Y* (mg/L)^17^	isolated from *S. formicae* KY5 Δ*forJ*Δ*forY* (mg/L)
**A** (**11**)^17^	522	3.3	29.0
**B** (**12**)^17^	556	2.5	25.1
**C (23**)^17^	570	1.3	not seen
**D** (**22**)^17^	590	3.3	36.6
**E** (**24**)^17^	604	1.6	2.1
**F** (**25**) (new)	556	n/a	7.4

We saw an order of magnitude increase in production of lactone intermediate A (**11**), B (**12**), and D (**22**) on deletion of the *forJ* gene from *S. formicae* KY5 Δ*forY*, but only a moderate increase for lactone intermediate E (**24**) and a loss of lactone intermediate C (**23**). We also found a new lactone intermediate, F (**25**) from *S. formicae* KY5 Δ*forJ*Δ*forY* ([Fig fig5]). This compound is a further example of a phenylnaphthacenoid with *ortho*-methoxy regiochemistry on the hanging E-ring.

**Figure 5:**
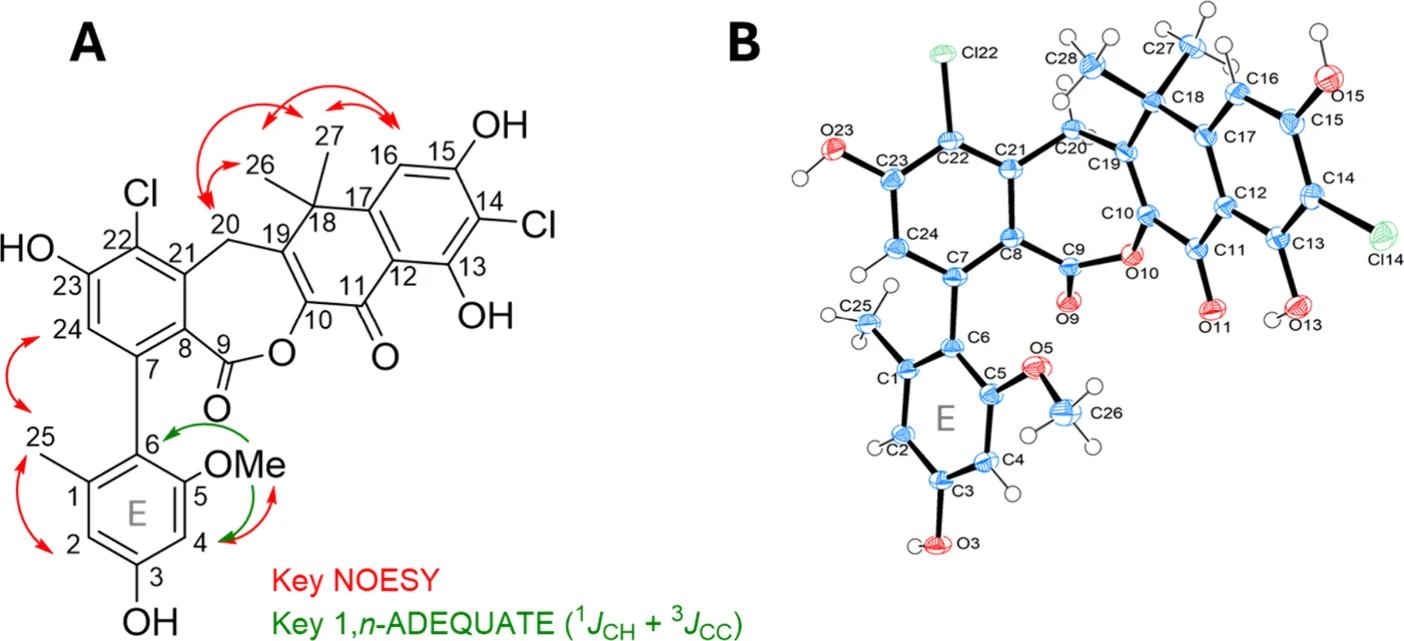
(A) Key NOESY and 1,*n*-ADEQUATE NMR correlations used to elucidate the structure of lactone intermediate F (**25**). (B) ORTEP representation of the crystal structure of **25**. Ellipsoids are given at the 50% probability level.

Lactone intermediate F (**25**) was obtained as an off-white powder, and high-resolution electrospray ionization mass spectrometric (HRESIMS) analysis indicated the presence of a species with molecular formula C_28_H_22_Cl_2_O_8_ (*m*/*z* 557.0768, [M + H]^+^). The isotopic peaks [M + H]^+^ (557.0768) and [M+H+2]^+^ (559.0741) had an abundance ration of 3:2, consistent with the presence of two chlorine atoms (Supporting Information, Figure S11). The extracted UV absorbance spectrum showed λ_max_ at 224, 250, and 317 nm. Examination of the ^1^H NMR spectrum (Table 6 and Supporting Information, Figure S12) showed three sets of methyl protons [δ_H_ 1.56 (s), 1.61 (s), and 2.02 (s)], a single methoxy group [δ_H_ 3.42 (s)], a diastereotopic pair of protons [δ_H_ 3.99 (d, *J* = 14.4 Hz) and 4.18 (d, *J* = 14.4 Hz)], and four aromatic protons; two had equal coupling constants consistent with an *meta* orientation [δ_H_ 6.25 (d, *J* = 1.8 Hz) and 6.32 (d, *J* = 1.8 Hz), while two were singlets [δ_H_ 6.69 (s), 6.73 (s)]. The ^13^C NMR spectrum (Supporting Information, Figure S13) was similar to previously published lactone intermediates, with a lactone carbonyl [δ_C_ 164.9] and a further downfield carbonyl [δ_C_ 182.3], suggesting the same carbon skeleton.^17^ The distribution of the two chlorine atoms was assigned first by NOESY NMR. The diastereotopic pair of protons H-20a (δ_H_ 3.99) and H-20b (δ_H_ 4.18) showed cross-peaks with one another and the methyl protons H-26 (δ_H_ 1.61) and H-27 (δ_H_ 1.56). The lack of NOESY cross-peaks from H-20a or H-20b with any aromatic protons suggested a chlorine atom at C-22. In addition to this, H-24 (δ_H_ 6.69), which is readily identified by its NOESY cross-peak with H-25 (δ_H_ 2.02), was present as a singlet suggesting no *meta* proton at C-22 (δ_C_ 117.4). The position of the second chlorine atoms was assigned by considering the NOESY cross-peaks from H-26 and H-27 with aromatic proton H-16 [δ_H_ 6.73 (s)]. H-16 is a singlet, suggesting there is no *meta* proton on C-14 (δ_C_ 107.2). C-22 and C-14 were assigned by HMBC correlations from H-24 and H-16, respectively. The regiochemistry of the methoxy group was determined as *ortho-* (on C-5) by 1,*n*-ADEQUATE NMR, which showed pseudo ^4^ *J*_CH_ coupling between the methoxy protons (δ_H_ 3.42) and C-4 (δ_C_ 97.4) and C-6 (δ_C_ 121.1) (Supporting Information, Figure S32). The remainder of the ^13^C NMR spectrum was assigned by a combination of HMBC correlations and a comparison with previously reported biosynthetic lactone intermediate spectra. The compound crystallized as a racemic mixture in the *C2*/*c* space group and diffracted to 0.76 Å resolution, thus confirming the structure by X-ray crystallography (Figure 5).

**Table 6:** ^1^H (600 MHz) and ^13^C (151 MHz) NMR Data for Lactone Intermediate F (**25**) in Methanol-*d*_4_ at 298 K^a^

position	δ_C_ ppm	δ_H_ ppm (number of protons, multiplicity, *J* in Hz)	HMBC	NOESY
1	138.7			
2	110.0	6.32 (1H, d, 1.8)	4, 5, 6, 25	25
3	158.9			
4	97.4	6.25 (1H, d, 1.8)	2, 3, 5, 6	5-O*Me*
5	158.1			
6	121.1			
7	141.9			
8	123.7			
9	164.9			
10	142.0			
11	182.3			
12	109.1			
13	161.3			
14	107.2			
15	161.9			
16	107.2	6.73 (1H, s)	11, 12, 13, 14, 15, 17, 18	26, 27
17	152.3			
18	42.2			
19	154.4			
20	29.4	4.18 (1H, d, 14.4)	8, 10, 19, 21, 22	26, 27
		3.99 (1H, d, 14.4)		
21	143.2			
22	117.4			
23	157.4			
24	120.1	6.69 (1H, s)	6, 7, 8, 9, 22, 23	25
25	20.6	2.02 (3H, s)	none seen	2, 24
26	28.8	1.61 (3H, s)	17, 18, 19, 27	16, 20
27	29.8	1.56 (3H, s)	17, 18, 19, 26	16, 20
5-O*Me*	56.0	3.42 (3H, s)	5	4

a HMBC correlations are from the proton(s) stated to the indicated carbon.

## Conclusions

Using single-crystal X-ray structure analysis of formicamycin H (**1**), we validated our previous characterization of the axial chirality of this molecule as *S*_a_. Further crystal structures for six formicamycin and four fasamycin metabolites were obtained, and all have the same *S*_a_ axial chirality suggesting that all such members of the phenylnaphthacenoid family of polyketide metabolites are produced biosynthetically as the *S*_a_ atropisomers. This is consistent with the three additional X-ray structures of streptovertimycins W and Z_1_ and accramycin A published previously^7,8^ and recent biochemical analysis of the polyketide cyclase enzyme FasU, which catalyzes formation of the biphenyl framework during fasamycin biosynthesis with strict *S*_a_ product stereospecificity.^23^ Moreover, we draw the attention to two notable technical aspects of the work presented here. First, as our misassignment of several phenylnaphthacenoid congeners shows, determining the regiochemistry of functional groups for highly substituted aromatic molecules can be problematic. To address this issue, we successfully applied the 1,*n*-ADEQUATE NMR experiment, which allows the visualization of pseudo ^4^ *J*_CH_ correlations (^1^ *J*_CH_ + ^3^ *J*_CC_) despite its relative insensitivity. Here, we found that samples of 10 mM concentration in conjunction with a 5 mm Shigemi tube were sufficient to allow cross-peaks to develop from the aromatic methoxy protons to C-4 and C-6 albeit with long experiment times. For example, the 1,*n*-ADEQUATE NMR of fasamycin M (Supporting Information, Figure S22) required 512 scans, which took 83 h. Second, we present 2:1 dichloromethane/cyclohexane as a versatile solvent system for producing phenylnaphthacenoid crystals by slow evaporation. This gave usable crystals for 13 out of 27 phenylnaphthacenoids tested. We commend both these techniques to colleagues researching phenylnaphthacenoids while understanding that the long NMR experiment times and diffractometer or synchrotron access for smaller crystals may pose a barrier. To that end, we are open to collaborating with groups who do not have their own access to such infrastructure.

Additionally, our X-ray crystallographic analysis led to the unexpected observation that lactone biosynthetic intermediates between fasamycin and formicamycins (obtained from mutant strains of *S. formicae* KY5 lacking the flavin-dependent reductase ForY) crystallized as racemic mixtures. This was surprising, as both fasamycin precursors and formicamycin products exhibit *S*_a_ axial chirality. Moreover, these lactone biosynthetic intermediates exhibit recordable optical rotations when measured, although the magnitude of these were significantly lower than recorded for fasamycin and formicamycins.^17^ Taken together, these observations suggest that the lactone intermediates can racemize in solution and are isolated as scalemic mixtures of reduced enrichment, which then crystallize as racemates. This is consistent with the results of DFT calculations for these species, which suggest a significantly lower barrier such that rotation about the C-6/C-7 bond can occur on a time scale that is slow but viable for racemization to occur, and which likely accounts for the loss of chirality observed. Thus, it appears that ForY must select only the *S*_a_ atropisomer as substrate. As lactone biosynthetic intermediates do not accumulate in the wild-type strain, this suggests ForY selects and converts lactone intermediates to formicamycins on a time scale faster than that of racemization or that racemization *in vivo* occurs more rapidly than our calculations suggest and that ForY effects an unusual stereoenrichment or deracemization process.

## Experimental Section

### General Experimental Procedures

Solvents used for extractions and HPLC were purchased from Fischer Scientific (UK). NMR solvents were purchased from Sigma-Aldrich (Merck). NMR spectra (1D and 2D) were recorded at 298 K on a Bruker Neo 600 MHz spectrometer equipped with a 5 mm TCI CryoProbe. Two-dimensional ^1^H–^1^H COSY, ^1^H–^13^C HSQCed, ^1^H–^13^C HMBC, and ^1^H–^1^H NOESY experiments were performed using standard pulse sequences from the Bruker Topspin library. 1,*n*-ADEQUATE experiments were performed using the standard 1,1-ADEQUATE pulse sequence with CNST3 adjusted to 7 Hz. Data were processed using TopSpin 4.0.1 and Mnova 15.0.1, and spectra were calibrated to either the ^1^H residual and ^13^C solvent signals (acetone-*d*_6_ δ_H_ 2.05, δ_C_ 29.8; DMSO-*d*_6_ δ_H_ 2.50, δ_C_ 39.5; methanol-*d*_4_ δ_H_ 3.31, δ_C_ 49.0) or where present TMS.^27^

For HRESIMS, the samples were dissolved into water + 0.1% formic acid/methanol (1:1) and infused into a Synapt G2-Si mass spectrometer (Waters, Manchester, U.K.) at 10 μL/min using a Harvard Apparatus syringe pump. The mass spectrometer was controlled with MassLynx 4.1 software (Waters). It was operated in a resolution and positive ion mode and calibrated using sodium iodide. The sample was analyzed for 1 min with a 1 s MS scan time over the *m*/*z* range 50–1200 with 2.0 kV capillary voltage, 40 V cone voltage, and 120 °C cone temperature. Leu-enkephalin peptide (1 ng/μL, Waters) was infused at 10 μL/min as a lock mass (*m*/*z* 556.2766) and measured every 10 s. Spectra were generated in MassLynx 4.1 by combining multiple scans, and peaks were centered using automatic peak detection with lock mass correction.

### Generation of *Streptomyces formicae* KY5 Δ*forJ* Δ*forY*

Growth media recipes are given in Table 7, and bacterial strains, plasmids, and primers are given in Tables 8, 9, and 10, respectively.

**Table 7:** Media Used in This Study

media	recipe (per liter)	water	pH
MS	20 g soy flour	tap	
	20 g mannitol		
	20 g agar		
MYM	4 g maltose	50:50 tap:deionized	7.3
	4 g yeast extract		
	10 g malt extract		
	± 18 g agar		
LB	10 g tryptone	deionized	7.5
	5 g yeast extract		
	10 g NaCl (omitted when selecting with hygromycin)		
	± 20 g agar		
2YT	16 g tryptone	deionized	7.0
	10 g yeast extract		
	5 g NaCl		

**Table 8:** Strains Used in This Study

strain	description/genotype	plasmid/resistance	
*E. coli* ET12567	dam^–^dcm^–^hsdS^–^	pUZ8002, Cml^R^/Tet^R^	MacNeil et al., 1992 ^28^
*S. formicae* KY5 Δ*forY*			Qin et al., 2020 ^17^
S. formicae KY5 Δ*forJ* Δ*forY*			this work

**Table 9:** Plasmids Used in This Study

plasmid	description	resistance	reference/source
pUZ8002	RK2 derivative with a mutation in oriT	Kan^R^	Keiser et al., 2000 ^29^
pCRISPomyces-2 *forJ* KO	pCRISPomyces-2 with gRNA specific for *forJ* and homology repair template designed to insert an in-frame delete of the *forJ* coding region	Apr^R^	Devine et al., 2021 ^19^

**Table 10:** Primers Used in This Study

primer	description	sequence (5′–3′)
ForJ KO Test 1F	Test *forJ* deletion in the genome	cctcttcggtgagcgcttcgagg
ForJ KO Test 2R	Test *forJ* deletion in the genome	cctgttggacttcgcgcaggc
ForJ KO Test 2F	Test *forJ* deletion in the genome	gtacgccaggaggacgtgcg
ForJ KO Test 1R	Test *forJ* deletion in the genome	gccgacgcggcacttctatcc

*S. formicae* KY5 ΔforJ ΔforY was generated by conjugating the pCRISPomyces-2 plasmid designed to delete forJ into spores of the existing *S. formicae* KY5 ΔforY mutant.^18^ To do this, single colonies of nonmethylating *Escherichia coli* ET12567/pUZ8002 containing the pCRISPomyces-2 forJ KO plasmid were selected from plates and grown in 10 mL of LB broth plus apramycin (50 μg/mL), kanamycin (50 μg/mL), and chloramphenicol (25 μg/mL) at 37 °C overnight at 220 rpm. Subcultures of OD600 between 0.4 and 0.6 were washed twice in LB to remove antibiotics. 200 μL of *S. formicae* KY5 ΔforY spores were heat shocked at 50 °C for 10 min in 500 μL of 2YT to encourage germination and added to the washed *E. coli* cells. The cell mixture was pelleted by centrifugation at 15,871 × *g* for 1 min, the supernatant was removed, and the cells were resuspended in the residual liquid. This was plated onto soya flour mannitol (SFM) agar containing 10 mM MgCl_2_ and incubated at 30 °C for 20 h. For the selection of desired ex-conjugants, 0.5 mg of nalidixic acid and 1 mg of apramycin were added in 1 mL of dH_2_O to each plate, giving final selection concentrations of 25 and 50 μg/mL, respectively, and cultures were returned to the 30 °C incubator for 5 days or until colonies appeared. Following purification by restreaking on antibiotic selective media, ex-conjugants were confirmed by colony PCR. Single colonies were picked and soaked in 100 μL of 50% DMSO at 50 °C for 1 h. This was then used as a template for PCR using PCRBIO Taq Mix Red (PCR Biosystems) at 10% of the final volume of the reaction (2.5 μL in 25 μL). To encourage loss of the pCRISPomyes-2 plasmid after mutagenesis had occurred, colonies were restreaked on nonselective media and incubated at 37 °C for multiple generations until apramycin resistance was lost. Confirmed, unmarked mutants were then grown on MYM for stock or SFM for the production of metabolites from the formicamycin BGC.

### Scale-up Fermentation of *S. formicae* KY5 Δ*forJ*Δ*forY*

Starter cultures were produced by growing spores of *S. formicae* KY5 Δ*forJ*Δ*forY* in TSB (10 mL) at 30 °C for 2 days. The starter culture was spread onto of MS agar (100 μL per 25 mL plate; 120 plates for 3 L in total) and incubated at 30 °C for 10 days. The agar was sliced into small pieces and extracted by steeping in EtOAc (4 L) over 4 days. The agar was removed by filtration through muslin cloth, and EtOAc was dried over MgSO_4_, filtered, and evaporated under reduced pressure to give the crude extract.

### Preparative HPLC Method to Isolate Lactone Intermediate F (**25**)

The crude extract from *S. formicae* KY5 Δ*forJ*Δ*forY* (3 L) was subject to preparative HPLC using an Agilent 1260 system fitted with a Kinetex 5 μm XB-C_18_ 100 Å (250 × 21.2 mm) column and gradient elution. The following conditions were used: flow rate 20 mL/min, sample injection volume 200 μL; mobile phase A: water with 0.1% formic acid; mobile phase B: acetonitrile with 0.1% formic acid; elution gradient: *T* = 0 min, 5% B; *T* = 4 min, 5% B; *T* = 4.5 min, 50% B; *T* = 19 min, 98% B; *T* = 20.5 min, 98% B; *T* = 21 min, 5% B; *T* = 22 min, 5% B; UV–vis absorbance monitoring at 254 nm. This gave the known lactone intermediates A (**11**) (87.1 mg), B (**12**) (75.4 mg), D (**22**) (109.7 mg) E (**24**) (6.2 mg), and the new lactone intermediate F (**25**, 22.2 mg).

Lactone intermediate F (**25**) an off-white amorphous powder; [α]_D_^20^ = +1.0 (*c* 1.0, MeOH); UV (DAD) λ_max_ 224, 250, and 317 nm; ^1^H and ^13^C NMR data in CD_3_OD, Table 6; HRESIMS *m*/*z* 557.0768 [M + H]^+^ (calcd for C_28_H_23_Cl_2_O_8_^+^ 557.0764, Δ = 0.7 ppm). For NMR, see Table 6 and Supporting Information, Figures S12–S17 and S32).

### Obtaining Crystal Structures

Crystals were obtained by slow evaporation of a dichloromethane/cyclohexane (2:1) solvent mixture at room temperature for 2 weeks, unless otherwise noted. Suitable crystals were transferred into either 100% ethylene glycol or glycerol to facilitate stable mounting into LithoLoops (Molecular Dimensions). These were then flash cooled in liquid nitrogen before being sent to the synchrotron. With the exception of formicamycin R (**5**), X-ray data were recorded on beamline I04 at the Diamond Light Source (Oxfordshire, UK) using an EIGER2 XE 16 M hybrid photon counting detector (Dectris), with crystals maintained at 100 K by a Cryojet cryocooler (Oxford Instruments). For each sample, the wavelength was set to 0.689 Å, and the detector distance was set such that the largest inscribed circle on the detector captured data to 0.88 Å resolution. To maximize data completeness beyond 0.88 Å resolution, four 360° passes (of 3600 × 0.1° images each) were recorded at chi values of 0, 15, 30, and 45° with an estimated total dose 0f 0.5 MGy per pass. The resultant data were integrated and scaled using DIALS.^30^ Data for **5** were collected on a Rigaku Synergy diffractometer equipped with a sealed tube copper source confocal mirrors and were processed using CrysAlisPro.^31^

In all cases, structures were solved using SHELXT-2018 ^32^ and refined on *F* ^2^ using SHELXL-2025 within Olex2.^33^ Most compounds used for crystallization had previously been dissolved in DMSO and acetonitrile for biological assays, so these solvents may be seen in some crystal lattices. Disordered solvent in **1**, **9**, and **14** could not be fully modeled and was handled using the BYPASS algorithm,^34^ as implemented in Olex2. Data were deposited with the Cambridge Crystallographic Data Centre with reference numbers 2498167–2498181.

#### Crystallographic Data for **1**

Empirical formula C_30_H_27_Cl_3_O_8_, C_2_H_6_SO, 2­(H_2_O); formula weight = 736.02, crystal system: orthorhombic, space group *P*2_1_2_1_2_1_, *a* = 7.99171(6) Å, *b* = 19.36026(11) Å, *c* = 20.86324(8) Å; α = β = γ = 90°, *V* = 3227.99(3) Å^3^; *T* = 100(2) K; *Z* = 4; density (calculated): 1.514 g/cm^3^; μ­(synchrotron) = 0.38 mm^–1^; *F*(000) = 1536; crystal size: 0.32 × 0.05 × 0.05 mm^3^; 7678 [*R*_int_ = 0.0406, *R*_sigma_ = 0.0121] independent reflections. The final *R* indexes were *R*_1_ = 0.0390, *wR*_2_ = 0.1207 (*I* > 2σ­(*I*)); goodness of fit on *F* ^2^ was 1.02; Flack parameter = 0.032(7); Hooft parameter = 0.198(6). A solvent mark was applied to account for two molecules of H_2_O and one molecule of DMSO in the asymmetric unit. Crystallographic data for **1** have been deposited at the Cambridge Crystallographic Data Centre with the number CCDC 2498172.

#### Crystallographic Data for 2

Empirical formula C_30_H_27_Cl_3_O_8_, C_2_H_6_OS; formula weight = 699.99, crystal system: orthorhombic, space group *P*2_1_2_1_2_1_, *a* = 13.48888(13) Å, *b* = 15.03340(14) Å, *c* = 15.87274(19) Å; α = β = γ = 90°, *V* = 3218.73(5) Å^3^; *T* = 100(2) K; *Z* = 4; density (calculated): 1.445 g/cm^3^; μ­(synchrotron) = 0.373 mm^–1^; *F*(000) = 1456; crystal size: 0.08 × 0.08 × 0.02 mm^3^; 7363 [*R*_int_ = 0.0571, *R*_sigma_ = 0.0173] independent reflections. The final *R* indexes were *R*_1_ = 0.0357, and *wR*_2_ = 0.1009 (*I* > 2σ­(*I*)); goodness of fit on *F* ^2^ was 1.055; Flack parameter = −0.003(8); Hooft parameter = −0.005(8). Crystallographic data for **2** have been deposited at the Cambridge Crystallographic Data Centre with the number CCDC 2498171.

#### Crystallographic Data for **3**

Empirical formula C_29_H_24_Cl_4_O_8_, C_2_H_6_OS; formula weight = 720.41, crystal system: orthorhombic, space group *P*2_1_2_1_2_1_, *a* = 12.85370(3) Å, *b* = 15.35654(3) Å, *c* = 15.78104(3) Å; α = β = γ = 90°, *V* = 3114.994(11) Å^3^; *T* = 100(2) K; *Z* = 4; density (calculated): 1.536 g/cm^3^; μ­(synchrotron) = 0.464 mm^–1^; *F*(000) = 1488; crystal size: 0.15 × 0.1 × 0.08 mm^3^; 7426 [*R*_int_ = 0.0405, *R*_sigma_ = 0.0123] independent reflections. The final *R* indexes were *R*_1_ = 0.0297, and *wR*_2_ = 0.0825 (*I* > 2σ­(*I*)); goodness of fit on *F* ^2^ was 1.153; Flack parameter = 0.002(5); Hooft parameter = 0.000(7). Crystallographic data for **3** have been deposited at the Cambridge Crystallographic Data Centre with the number CCDC 2498173.

#### Crystallographic Data for **4**

Empirical formula C_30_H_26_Cl_4_O_8_, C_2_H_6_OS; formula weight = 734.43, crystal system: orthorhombic, space group *P*2_1_2_1_2_1_, *a* = 13.25533(6) Å, *b* = 15.40111(5) Å, *c* = 15.73892(7) Å; α = β = γ = 90°, *V* = 3213.05(2) Å^3^; *T* = 100(2) K; *Z* = 4; density (calculated): 1.518 g/cm^3^; μ­(synchrotron) = 0.45 mm^–1^; *F*(000) = 1520; crystal size: 0.1 × 0.02 × 0.02 mm^3^; 7542 [*R*_int_ = 0.0409, *R*_sigma_ = 0.0127] independent reflections. The final *R* indexes were *R*_1_ = 0.0702, *wR*_2_ = 0.1654 (*I* > 2σ­(*I*)); goodness of fit on *F* ^2^ was 1.051; Flack parameter = 0.028(7); Hooft parameter = 0.024(8). Crystallographic data for **4** have been deposited at the Cambridge Crystallographic Data Centre with the number CCDC 2498174.

#### Crystallographic Data for **5**

Crystals of **5** were obtained by slow evaporation from methanol at 4 °C. Empirical formula C_29_H_23_Cl_5_O_8_, CH_4_O; formula weight = 708.76, crystal system: tetragonal, space group *P*4_1_2_1_2, *a* = *b* = 16.0671(3) Å, *c* = 23.0813(5) Å; α = β = γ = 90°, *V* = 5958.5(3) Å^3^; *T* = 116(20) K; *Z* = 8; density (calculated): 1.580 g/cm^3^; λ­(Cu Kα) = 4.921 mm^–1^; *F*(000) = 2912; crystal size: 0.084 × 0.014 × 0.010 mm^3^; 5873 [*R*_int_ = 0.0916, *R*_sigma_ = 0.0366] independent reflections. The final *R* indexes were *R*_1_ = 0.0420, and *wR*_2_ = 0.1146 (*I* > 2σ­(*I*)); goodness of fit on *F* ^2^ was 1.119; Flack parameter = 0.005(9); Hooft parameter = 0.007(5). Crystallographic data for **5** have been deposited at the Cambridge Crystallographic Data Centre with the number CCDC 2498175.

#### Crystallographic Data for **6**

Empirical formula C_30_H_25_Cl_5_O_8_, C_2_H_6_OS; formula weight = 768.88, crystal system: orthorhombic, space group *P*2_1_2_1_2_1_, *a* = 13.77708(3) Å, *b* = 15.39046(3) Å, *c* = 15.80963(4) Å; α = β = γ = 90°, *V* = 3352.204(13) Å^3^; *T* = 100(2) K; *Z* = 4; density (calculated): 1.523 g/cm^3^; μ­(synchrotron) = 0.507 mm^–1^; *F*(000) = 1584; crystal size: 0.1 × 0.1 × 0.1 mm^3^; 7966 [*R*_int_ = 0.0563, *R*_sigma_ = 0.0149] independent reflections. The final *R* indexes were *R*_1_ = 0.0460, and *wR*_2_ = 0.1267 (*I* > 2σ­(*I*)); goodness of fit on *F* ^2^ was 1.036; Flack parameter = 0.001(5); Hooft parameter = 0.001(5). Crystallographic data for **6** have been deposited at the Cambridge Crystallographic Data Centre with the number CCDC 2498176.

#### Crystallographic Data for **7**

Empirical formula C_28_H_22_Cl_2_O_7_, 2­(C_2_H_6_OS); formula weight = 697.61, crystal system: monoclinic, space group *P*2_1_, *a* = 13.18845(7) Å, *b* = 8.46032(4) Å, *c* = 14.81152(10) Å; α = 90°, β = 100.4417(6)°, γ = 90°, *V* = 1625.279(16) Å^3^; *T* = 100(2) K; *Z* = 2; density (calculated): 1.426 g/cm^3^; μ­(synchrotron) = 0.353 mm^–1^; *F*(000) = 728; crystal size: 0.03 × 0.02 × 0.005 mm^3^; 7608 [*R*_int_ = 0.0798, *R*_sigma_ = 0.0378] independent reflections. The final *R* indexes were *R*_1_ = 0.0471, *wR*_2_ = 0.1288 (*I* > 2σ­(*I*)); goodness of fit on *F* ^2^ was 1.104; Flack parameter = 0.01(2); Hooft parameter = 0.01(2). Crystallographic data for **7** have been deposited at the Cambridge Crystallographic Data Centre with the number CCDC 2498167.

#### Crystallographic Data for **8**

Empirical formula C_28_H_21_Cl_3_O_7_, 3­(C_2_H_6_OS); formula weight = 810.18, crystal system: orthorhombic, space group *P*2_1_2_1_2_1_, *a* = 8.506 Å, *b* = 14.09410(10) Å, *c* = 31.1056(2) Å; α = β = γ = 90°, *V* = 3729.08(4) Å^3^; *T* = 100(2) K; *Z* = 4; density (calculated): 1.443 g/cm^3^; μ­(synchrotron) = 0.433 mm^–1^; *F*(000) = 1688; crystal size: 0.1 × 0.09 × 0.03 mm^3^; 8656 [*R*_int_ = 0.0334, *R*_sigma_ = 0.0123] independent reflections. The final *R* indexes were *R*_1_ = 0.0393, *wR*_2_ = 0.1181 (*I* > 2σ­(*I*)); goodness of fit on *F* ^2^ was 1.051; Flack parameter = 0.000(5); Hooft parameter = −0.003(8). Crystallographic data for **8** have been deposited at the Cambridge Crystallographic Data Centre with the number CCDC 2498168.

#### Crystallographic Data for **9**

Empirical formula C_28_H_21_Cl_3_O_7_, C_2_H_6_OS, 3.5­(H_2_O); formula weight = 716.98, crystal system: monoclinic, space group *P*2_1_, *a* = 17.25293(10) Å, *b* = 8.94502(5) Å, *c* = 20.71028(13) Å; α = 90°, β = 95.8290(50)°, γ = 90°, *V* = 3179.63(3) Å^3^; *T* = 100(2) K; *Z* = 4; density (calculated): 1.498 g/cm^3^; μ­(synchrotron) = 0.384 mm^–1^; *F*(000) = 1492; crystal size: 0.04 × 0.02 × 0.02 mm^3^; 14835 [*R*_int_ = 0.0549, *R*_sigma_ = 0.0264] independent reflections. The final *R* indexes were *R*_1_ = 0.0578, and *wR*_2_ = 0.1698 (*I* > 2σ­(*I*)); goodness of fit on *F* ^2^ was 1.086; Flack parameter = 0.031(11); Hooft parameter = 0.035(12). A solvent mark was applied to account for six molecules of H_2_O in the asymmetric unit. Crystallographic data for **9** have been deposited at the Cambridge Crystallographic Data Centre as CCDC 2498169.

#### Crystallographic Data for **10**

Empirical formula C_28_H_20_Cl_4_O_7_, H_2_O; formula weight = 628.25, crystal system: triclinic, space group *P*1, *a* = 8.46930(2) Å, *b* = 12.13407(3) Å, *c* = 14.03789(4) Å; α = 106.9555(2)°, β = 97.8203(2)°, γ = 108.8169(2)°, *V* = 1263.275(6) Å^3^; *T* = 100(2) K; *Z* = 2; density (calculated): 1.652 g/cm^3^; μ­(synchrotron) = 0.483 mm^–1^; *F*(000) = 644; crystal size: 0.04 × 0.02 × 0.01 mm^3^; 10593 [*R*_int_ = 0.0168, *R*_sigma_ = 0.0098] independent reflections. The final *R* indexes were *R*_1_ = 0.0288, and *wR*_2_ = 0.0821 (*I* > 2σ­(*I*)); goodness of fit on *F* ^2^ was 1.065; Flack parameter = −0.003(6); Hooft parameter = −0.005(4). Crystallographic data for **10** have been deposited at the Cambridge Crystallographic Data Centre as CCDC 2498170.

#### Crystallographic Data for **11**

Empirical formula C_28_H_23_ClO_8_, 2­(CH_4_O); formula weight = 587, crystal system: monoclinic, space group *C*2/*c*, *a* = 16.46612(4) Å, *b* = 15.79752(3) Å, *c* = 20.38196(4) Å; α = 90°, β = 91.9490(2)°, γ = 90°, *V* = 5298.766(19) Å^3^; *T* = 100(2) K; *Z* = 8; density (calculated): 1.472 g/cm^3^; μ­(synchrotron) = 0.191 mm^–1^; *F*(000) = 2464; crystal size: 0.1 × 0.03 × 0.002 mm^3^; 5729 [*R*_int_ = 0.0212, *R*_sigma_ = 0.0060] independent reflections. The final *R* indexes were *R*_1_ = 0.0721, *wR*_2_ = 0.2165 (*I* > 2σ­(*I*)); goodness of fit on *F* ^2^ was 1.083. One ring, one methyl group, and one solvent molecule were disordered over two positions. Crystallographic data for **11** have been deposited at the Cambridge Crystallographic Data Centre with the number CCDC 2498177.

#### Crystallographic Data for **12**

Empirical formula C_28_H_22_Cl_2_O_8_; formula weight = 557.35, crystal system: monoclinic, space group *P*2_1_/*n*, *a* = 9.69605(4) Å, *b* = 8.6293(4) Å, *c* = 28.93986(12) Å; α = 90°, β = 90.2857(4)°, γ = 90°, *V* = 2421.277(8) Å^3^; *T* = 100(2) K; *Z* = 4; density (calculated): 1.529 g/cm^3^; μ­(synchrotron) = 0.298 mm^–1^; *F*(000) = 1152; crystal size: 0.05 × 0.02 × 0.02 mm^3^; 5690 [*R*_int_ = 0.0395, *R*_sigma_ = 0.0129] independent reflections. The final *R* indexes were *R*_1_ = 0.0377, *wR*_2_ = 0.1016 (*I* > 2σ­(*I*)); goodness of fit on *F* ^2^ was 1.042. Crystallographic data for **12** have been deposited at the Cambridge Crystallographic Data Centre with CCDC 2498178.

#### Crystallographic data for **13**

Empirical formula C_27_H_23_NO_6_, CH_4_O; formula weight = 489.5, crystal system: monoclinic, space group *P*2_1_/*n*, *a* = 10.33500(10) Å, *b* = 8.82460(10) Å, *c* = 26.7519(3) Å; α = 90°, β = 100.9280(10)°, γ = 90°, *V* = 2395.59(5) Å^3^; *T* = 100(2) K; *Z* = 4; density (calculated): 1.357 g/cm^3^; μ­(synchrotron) = 0.092 mm^–1^; *F*(000) = 1032; crystal size: 0.2 × 0.05 × 0.04 mm^3^; 5546 [*R*_int_ = 0.1414, *R*_sigma_ = 0.0443] independent reflections. The final *R* indexes were *R*_1_ = 0.0568, *wR*_2_ = 0.1650 (*I* > 2σ­(*I*)); goodness of fit on *F* ^2^ was 1.096. Crystallographic data for **13** have been deposited at the Cambridge Crystallographic Data Centre with CCDC 2498180.

#### Crystallographic Data for **14**

Crystals of **14** were obtained by slow evaporation from a benzene/methanol mixture (1:1) at room temperature. Empirical formula C_27_H_22_ClNO_6_, C_6_H_6_, CH_4_O; formula weight = 602.05, crystal system: monoclinic, space group *P*2_1_/*n*, *a* = 7.98735(5) Å, *b* = 15.13713(7) Å, *c* = 24.81571(11) Å; α = 90°, β = 98.4291(5)°, γ = 90°, *V* = 2967.95(3) Å^3^; *T* = 100(2) K; *Z* = 4; density (calculated): 1.347 g/cm^3^; μ­(synchrotron) = 0.167 mm^–1^; *F*(000) = 1264; crystal size: 0.25 × 0.015 × 0.015 mm^3^; 6948 [*R*_int_ = 0.0493, *R*_sigma_ = 0.0174] independent reflections. The final *R* indexes were *R*_1_ = 0.0523, *wR*_2_ = 0.1671 (*I* > 2σ­(*I*)); goodness of fit on *F* ^2^ was 1.089. A solvent mask was applied to account for one MeOH in the asymmetric unit. Crystallographic data for **14** have been deposited at the Cambridge Crystallographic Data Centre with the number CCDC 2498181.

#### Crystallographic Data for **25**

Empirical formula C_28_H_22_Cl_2_O_8_, 2­(H_2_O), C_2_H_3_N; formula weight = 634.44, crystal system: monoclinic, space group *C*2/*c*, *a* = 20.70500(10) Å, *b* = 13.144 Å, *c* = 21.31640(10) Å; α = 90°, β = 93.59°, γ = 90°, *V* = 5789.80(4) Å^3^; *T* = 100(2) K; *Z* = 8; density (calculated): 1.456 g/cm^3^; μ­(synchrotron) = 0.264 mm^–1^; *F*(000) = 2640; crystal size: 0.2 × 0.05 × 0.04 mm^3^; 6366 [*R*_int_ = 0.0597, *R*_sigma_ = 0.0169] independent reflections. The final *R* indexes were *R*_1_ = 0.0337, *wR*_2_ = 0.1028 (*I* > 2σ­(*I*)); goodness of fit on *F* ^2^ was 1.073. Crystallographic data for **25** have been deposited at the Cambridge Crystallographic Data Centre with the number CCDC 2498179.

### DFT Experiments

All calculations were performed using the Orca 6.0.1 computational package.^35^ Geometry optimizations were carried out using the all-electron 6-31G­(d) basis set and the B3LYP hybrid functional.^36,37^ The "tight" setting in Orca was used during self-consistent field convergence calculations. Initial geometries were taken from X-ray coordinates and were minimized prior to the energy calculation for the rotational minima. Maxima were located in three steps: a coarse sweep of the C-6/C-7 dihedral angle (10° steps) was used to identify the approximate locations, followed by a finer (1°) sweep to give a good approximation for the rotational transition states. These were then used to carry out transition state searches in Orca, which yielded the final geometries and energies for the maxima.

## Supplementary Material



## Data Availability

Crystallographic data have been deposited at the Cambridge Crystallographic Data Centre (CCDC; www.ccdc.cam.ac.uk), and relevant deposition codes are provided in the Experimental Section. The raw NMR data have been deposited in the Natural Products Magnetic Resonance Database (NP-MRD; www.np-mrd.org) and can be found at NP0016596 (formicamycin H (**1**)); NP0352156 (fasamycin C/napthacemycin B_2_ (**17**)), NP0352157 (fasamycin E/napthacemycin B_7_ (**7**)), NP0352158 (fasamycin L/napthacemycin B_11_ (**8**)), NP0352159 (fasamycin Q/napthacemycin B_12_ (**21**)), NP0352160 (lactone intermediate F (**25**)), NP0352161 (fasamycin M (**9**)), NP0352162 (fasamycin N (**20**)), NP0352163 (formicamycin B (**15**)), NP0352164 (formicamycin D (**16**)), NP0352165 (formicamycin I (**3**)), NP0352166 (formicamycin R (**5**)), NP0352167 (lactone intermediate A (**11**)), NP0352168 (lactone intermediate B (**12**)), and NP0352169 (lactone intermediate D (**22**)). All other data are provided within the body of the manuscript or associated electronic Supporting Information. Requests for materials should be addressed to the corresponding authors.

## Notes

Comparison of ^1^H and ^13^C NMR spectra with published data, 1D and 2D NMR spectra for lactone intermediate F (**25**), 1,*n*-ADEQUATE NMR spectra, and DFT coordinates (PDF)
