# Supplementary material for: Using X‑ray Crystallography and 1,n‑ADEQUATE NMR to Revise the Structures of Highly Substituted Aromatic Natural Products: The Absolute Configuration of Formicamycin Congeners
Source: J Nat Prod. 2026 Apr 16;89(8):2238–52. doi: 10.1021/acs.jnatprod.6c00145 (PMC13532170; doi:10.1021/acs.jnatprod.6c00145)
Supplement: Supplementary file 1 [file np6c00145_si_001.pdf]

Supporting Information

Using X-ray Crystallography and 1,*n*-ADEQUATE NMR to Revise the Structures of Highly Substituted Aromatic Natural Products: The Absolute Configuration of Formicamycin Congeners

Edward S. Hems[1,2*], Joseph A. Wright[3], Sergey A. Nepogodiev[4], Rebecca Devine[1,2], Corinne J. Arnold[1], David L. Hughes,[3] Julia E. A. Mundy[5], Sandra Eltschkner[5], David M. Lawson[5], Matthew I. Hutchings[1,2*] and Barrie Wilkinson[1,2*]

1. Department of Molecular Microbiology, John Innes Centre, Norwich Research Park, Norwich, NR4 7UH, United Kingdom

2. Centre for Microbial Interactions, Norwich Research Park, Norwich, NR4 7UG, United Kingdom

3. School of Chemistry, University of East Anglia, Norwich NR4 7TJ, United Kingdom

4. NMR Platform, John Innes Centre, Norwich Research Park, Norwich, NR4 7UH, United Kingdom

5. Biochemistry and Metabolism, John Innes Centre, Norwich Research Park, Norwich, NR4 7UH, United Kingdom

*Corresponding Authors: Edward S. Hems (edward.hems@jic.ac.uk), Matthew I. Hutchings (matt.hutchings@jic.ac.uk) and Barrie Wilkinson (barrie.wilkinson@jic.ac.uk)

Table of Contents

1. Comparison of NMR spectra for natural and synthetic formicamycin H (1) 3

2. Comparison of published NMR data for napthacemycin B_2 with NMR of fasamycin C (17)...... 7

3. Comparison of published NMR data for napthacemycin B_4 with NMR of fasamycin D (18).... 13

4. Comparison of published NMR for napthacemycin B_7 with NMR of fasamycin E (7) 15

5. Comparison of published NMR data for napthacemycin B_{11} with NMR of fasamycin L (8)..... 19

6. Comparison of published NMR data for napthacemycin B_{12} with NMR of fasamycin Q (21) .. 23

7. High-resolution electrospray ionisation mass spectrometry (HRESIMS) of lactone intermediate F (25) 27

8. NMR spectra for lactone intermediate F (25) 28

9. Re-examining the published NOESY NMR of fasamycin C (17) 34

10. 1,*n*-ADEQUATE NMR experiments 35

11. DFT Coordinates... 49

12. REFERENCES 73

1. Comparison of NMR spectra for natural and synthetic formicamycin H (1)

Table S1. Comparison of ^{1}H NMR data for synthetic and natural formicamycin H (**1**) in $CDCl_3$

	**Synthetic** Hu *et al.*[1]	**Natural (1)** This study	
Position	δ ^{1}H ($CDCl_3$, 500 MHz)	δ ^{1}H ($CDCl_3$, 600 MHz)	Δδ ^{1}H (ppm)
1	-	-	-
2	-	-	-
3	-	-	-
4	6.52 (s)	6.52 (s)	0.00
5	-	-	-
6	-	-	-
7	-	-	-
8	-	-	-
9	-	-	-
10	-	-	-
10-OH	5.15 (s)	5.15 (s)	0.00
11	-	-	-
12	-	-	-
13	-	-	-
13-OH	13.26 (s)	13.26 (s)	0.00
14	-	-	-
15	-	-	-
15-OH	6.80 (brs)	6.80 (brs)	0.00
16	-	-	-
17	-	-	-
18	-	-	-
19	2.55 (dd, *J* = 12.0, 5.5 Hz)	2.55 (dd, *J* = 12.0, 5.6 Hz)	0.00
20'	2.96 (dd, *J* = 17.9, 12.0 Hz)	2.96 (dd, *J* = 18.0, 12.2 Hz)	0.00
20''	3.43 (dd, *J* = 18.2, 5.8 Hz)	3.42 (dd, *J* = 18.1, 5.6 Hz)	0.01
21	-	-	-
22	6.68 (d, *J* = 2.5 Hz)	6.68 (d, *J* = 2.3 Hz)	0.00
23	-	-	-
24	6.59 (d, *J* = 2.5 Hz)	6.59 (d, *J* = 2.5 Hz)	0.00
25	2.05 (s)	2.05 (s)	0.00
26	1.91 (s)	1.91 (s)	0.00
27	1.75 (s)	1.75 (s)	0.00
28	3.85 (s)	3.85 (s)	0.00
29	3.97 (s)	3.97 (s)	0.00
30	3.72 (s)	3.72 (s)	0.00

Table S2. Comparison of ^{13}C NMR data for synthetic and natural formicamycin H (**1**) in $CDCl_3$

	**Synthetic** Huo *et al.*[1]	**Natural (1)** This study	
Position	δ ^{13}C ($CDCl_3$, 151 MHz)	δ ^{13}C ($CDCl_3$, 151 MHz)	Δδ ^{13}C (ppm)
1	135.4	135.4	0.0
2	114.8	114.8	0.0
3	155.0	155.0	0.0
4	94.2	94.2	0.0
5	155.7	155.7	0.0
6	123.7	123.7	0.0
7	142.7	142.7	0.0
8	121.7	121.7	0.0
9	192.9	192.9	0.0
10	79.2	79.2	0.0
10-OH	-	-	-
11	198.7	198.6	0.0
12	107.4	107.4	0.0
13	155.9	155.9	0.0
13-OH	-	-	-
14	112.5	112.5	0.0
15	160.2	160.2	0.0
15-OH	-	-	-
16	110.1	110.1	0.0
17	145.8	145.8	0.0
18	39.9	39.9	0.0
19	51.1	51.1	0.0
20'	30.9	30.9	0.0
20''	-	-	-
21	145.3	145.3	0.0
22	112.8	112.8	0.0
23	163.8	163.8	0.0
24	117.8	117.8	0.0
25	17.8	17.8	0.0
26	28.9	28.9	0.0
27	28.0	27.9	0.1
28	55.7	55.7	0.0
29	56.3	56.3	0.0
30	56.3	56.3	0.0

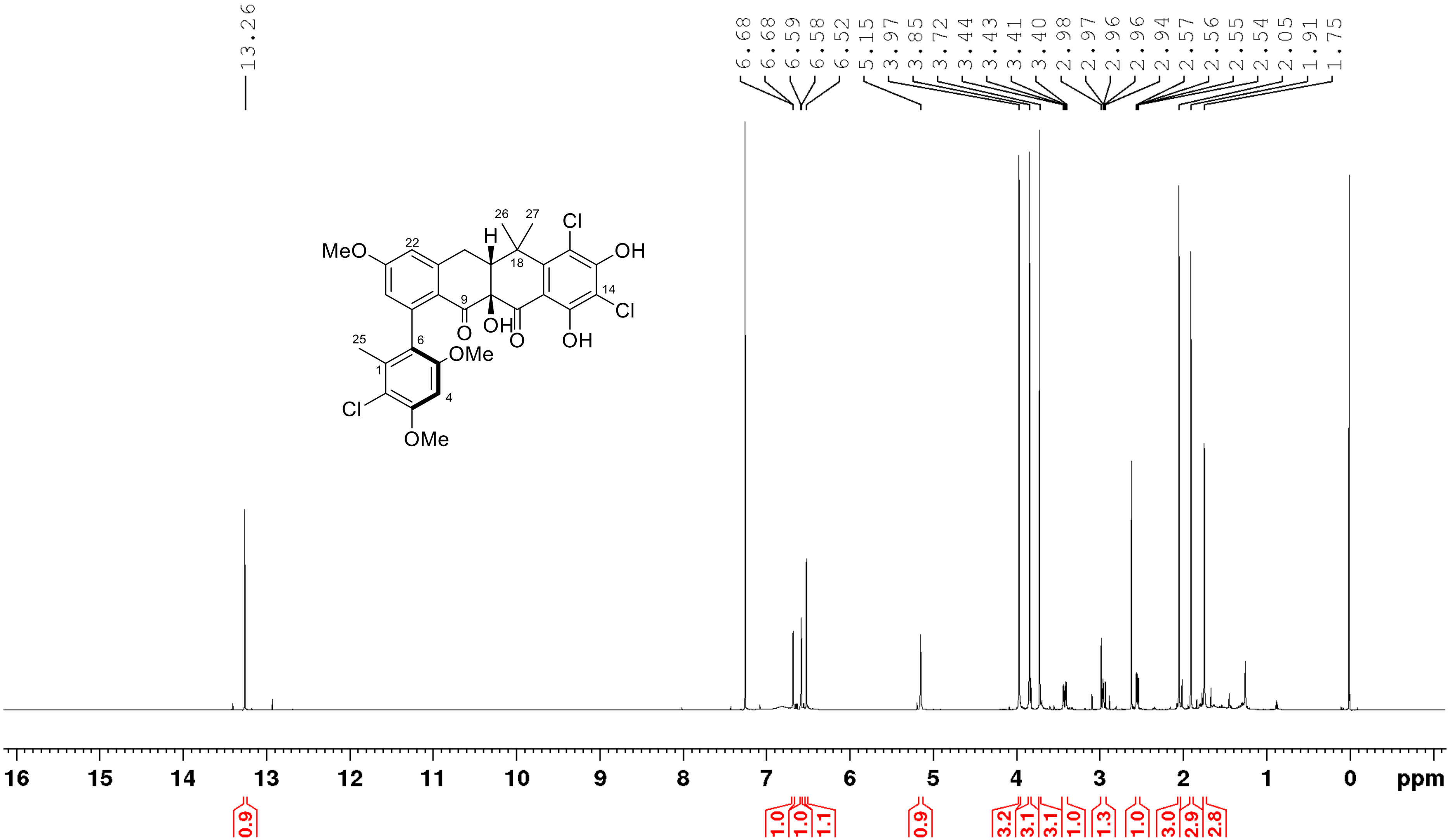

Figure S1. ^{1}H NMR of natural formicamycin H (**1**) (this study) in $CDCl_3$ at 298 K.

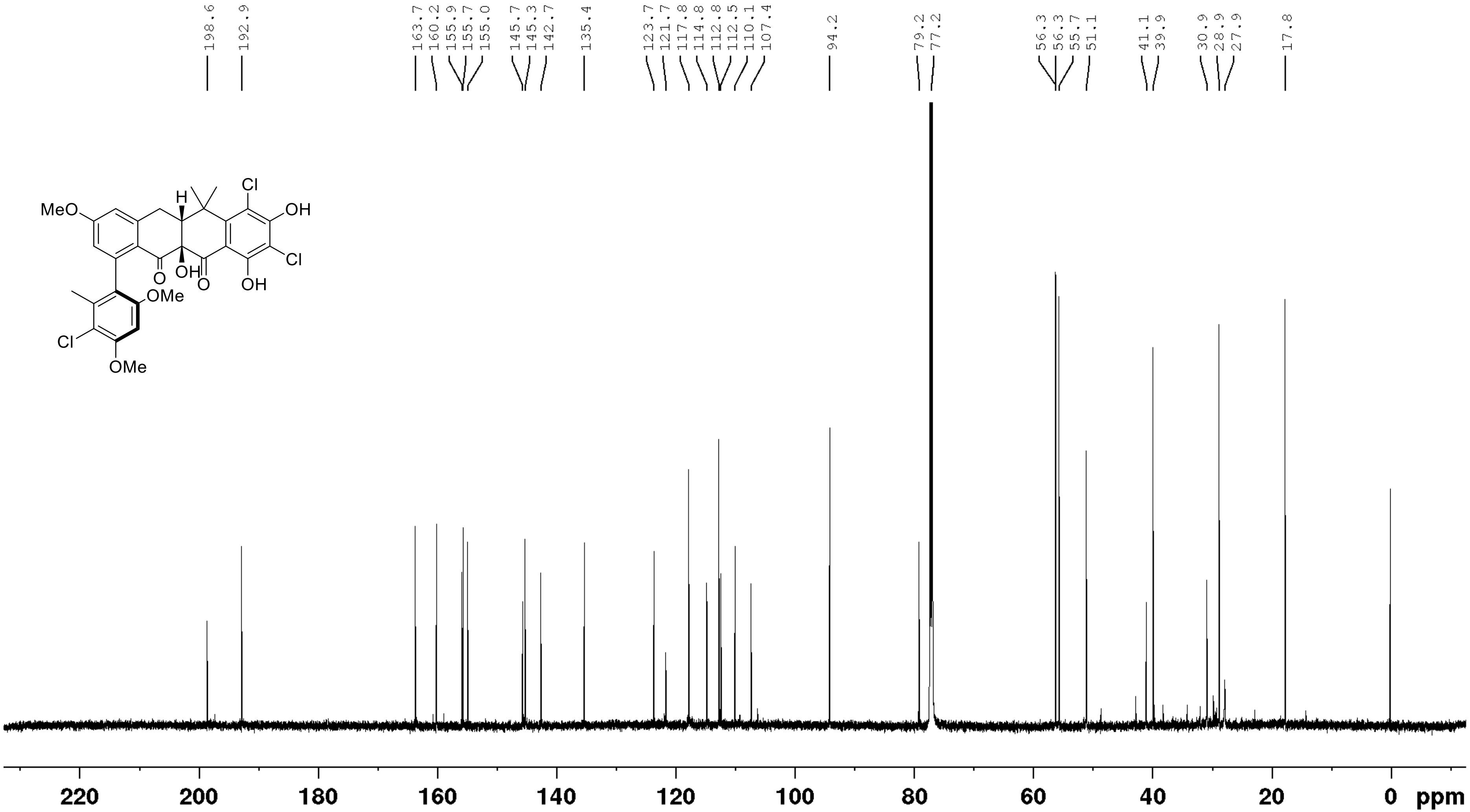

Figure S2. ^{13}C NMR of natural formicamycin H **(1)** (this study) in $CDCl_3$ at 298 K.

2. Comparison of published NMR data for napthacemycin B_2 with NMR of fasamycin C (17)

Table S3. Comparison of ^1H NMR data for napthacemycin B_2 (Fukumoto *et al., 2017*)[2] and fasamycin C (**17**) in acetone-d_6

	**Naphthacemycin B_2** Fukumoto *et al.*[2]	**Fasamycin C (17)** This study	
Position	δ ^1H (acetone-d_6, 300 MHz)	δ ^1H (acetone-d_6, 600 MHz)	Δδ ^1H (ppm)
1	-	-	-
2	6.51	6.39	-0.12
3	-	-	-
4	6.50	6.38	-0.12
5	-	-	-
5-OMe	3.49	3.56	0.07
6	-	-	-
7	-	-	-
8	-	-	-
9	-	-	-
9-OH	14.50	14.51	0.01
10	-	-	-
11	-	-	-
12	-	-	-
13	-	-	-
13-OH	12.87	12.89	0.02
14	6.33	6.29	-0.04
15	-	-	-
16	6.68	6.75	0.07
17	-	-	-
18	-	-	-
19	-	-	-
20	-	-	-
21	7.34	7.47	0.13
22	7.18	7.16	-0.02
23	-	-	-
24	6.88	6.76	-0.12
25	1.91	1.89	-0.02
26	1.57	1.76	0.19
27	1.53	1.72	0.19

Table S4. Comparison of ^{13}C NMR data for napthacemycin B_2 (Fukumoto *et al., 2017*)[2] and fasamycin C (**17**) in acetone-d_6

	**Naphthacemycin B_2** Fukumoto *et al.*[2]	**Fasamycin C (17)** This study	
Position	δ ^{13}C (acetone-d_6, 75 MHz)	δ ^{13}C (acetone-d_6, 151 MHz)	Δδ ^{13}C (ppm)
1	137.9	137.3	-0.6
2	109.4	108.9	-0.5
3	158.4	157.8	-0.6
4	97.6	97.1	-0.5
5	158.9	158.5	-0.4
5-OMe	55.9	55.7	-0.2
6	125.6	125	-0.6
7	141.2	140.8	-0.4
8	118	117.7	-0.3
8-OH	-	-	-
9	160.4*	166.4	6.0
10	107.6	107.2	-0.4
11	191.2	191.1	-0.1
12	108.4	108.2	-0.2
13	166.6	166.3	-0.3
13-OH	-	-	-
14	102.2	102	-0.2
15	166.9	166.3	-0.6
16	107.3	107.1	-0.2
17	155.6	155.5	-0.1
18	39.5	39.4	-0.1
19	146.3	146	-0.3
20	116	116	
21	142.8	142.4	-0.4
22	110.1	109.8	-0.3
23	166.7*	159.7	-7.0
24	122.6	122	-0.6
25	20.9	20.6	-0.3
26	34.5	34.7	0.2
27	34.7	34.3	-0.4

* note that we have assigned the signals corresponding to C-9 and C-23 the opposite way around to Fukumoto *et al*. and allowing for this brings the Δδ ^{13}C to -0.7 ppm and -0.3 ppm for C-9 and C-23 respectively.

Table S5. Comparison of ^1H NMR data for napthacemycin B_2 (Huo *et al.* 2020)[3] and fasamycin C (**17**) in acetone-d_6

	**Naphthacemycin B_2** Huo *et al.*[3]	**Fasamycin C (17)** This study	
Position	δ ^1H (acetone-d_6, 500 MHz)	δ ^1H (acetone-d_6, 600 MHz)	Δδ ^1H (ppm)
1	-	-	-
2	6.39	6.39	0.00
3	-	-	-
4	6.39	6.38	-0.01
5	-	-	-
5-OMe	3.57	3.56	-0.01
6	-	-	-
7	-	-	-
8	-	-	-
9	-	-	-
9-OH	14.46	14.51	0.05
10	-	-	-
11	-	-	-
12	-	-	-
13	-	-	-
13-OH	12.84	12.89	0.05
14	6.29	6.29	0.00
15	-	-	-
16	6.78	6.75	-0.03
17	-	-	-
18	-	-	-
19	-	-	-
20	7.48	7.47	-0.01
21	-	-	-
22	7.17	7.16	-0.01
23	-	-	-
24	6.76	6.76	0.00
25	1.9	1.89	-0.01
26	1.74	1.76	0.02
27	1.73	1.72	-0.01

Table S6. Comparison of ^{13}C NMR data for napthacemycin B_2 (Huo *et al.* 2020)[3] and fasamycin C (**17**) in acetone-d_6

	**Naphthacemycin B_2** Huo *et al.*[3]	**Fasamycin C (17)** This study	
Position	δ ^{13}C (acetone-d_6, 125 MHz)	δ ^{13}C (acetone-d_6, 151 MHz)	Δδ ^{13}C (ppm)
1	137.3	137.3	0.0
2	109	108.9	-0.1
3	157.8	157.8	0.0
4	97.2	97.1	-0.1
5	158.5	158.5	0.0
5-OMe	55.7	55.7	0.0
6	125.1	125	-0.1
7	141	140.8	-0.2
8	117.8	117.7	-0.1
8-OH	-	-	-
9	166.9	166.4	-0.5
10	107.3	107.2	-0.1
11	191.2	191.1	-0.1
12	108.4	108.2	-0.2
13	166.6	166.3	-0.3
13-OH	-	-	-
14	102	102	0.0
15	166.6	166.3	-0.3
16	106.9	107.1	0.2
17	155.6	155.5	-0.1
18	39.5	39.4	-0.1
19	146.1	146	-0.1
20	116	116	0.0
21	142.5	142.4	-0.1
22	109.9	109.8	-0.1
23	159.6	159.7	0.1
24	122	122	0.0
25	20.6	20.6	0.0
26	34.7	34.7	0.0
27	34.3	34.3	0.0

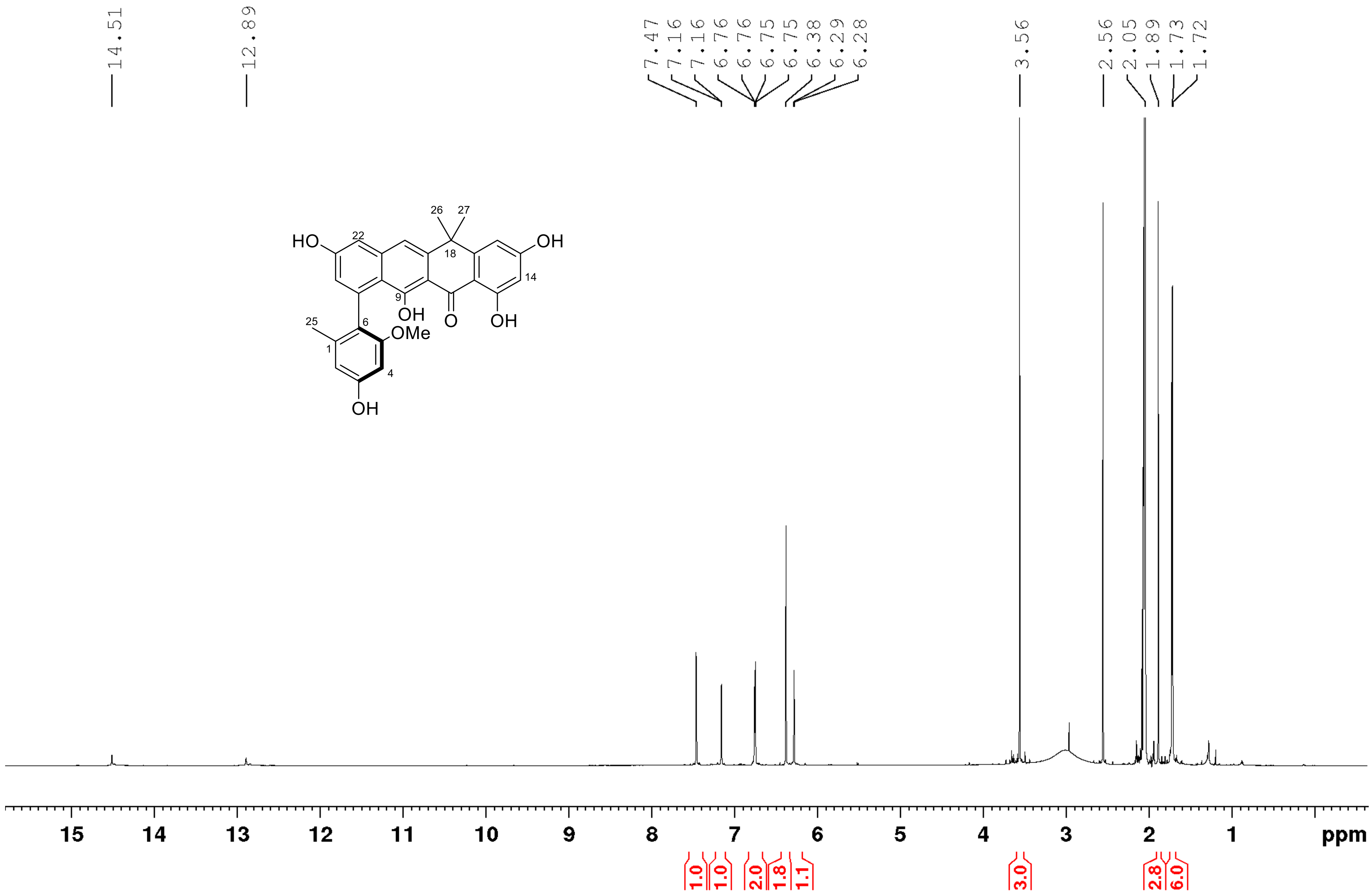

Figure S3. ^{1}H NMR (600 MHz) of fasamycin C (**17**) in acetone-d_6 at 298 K.

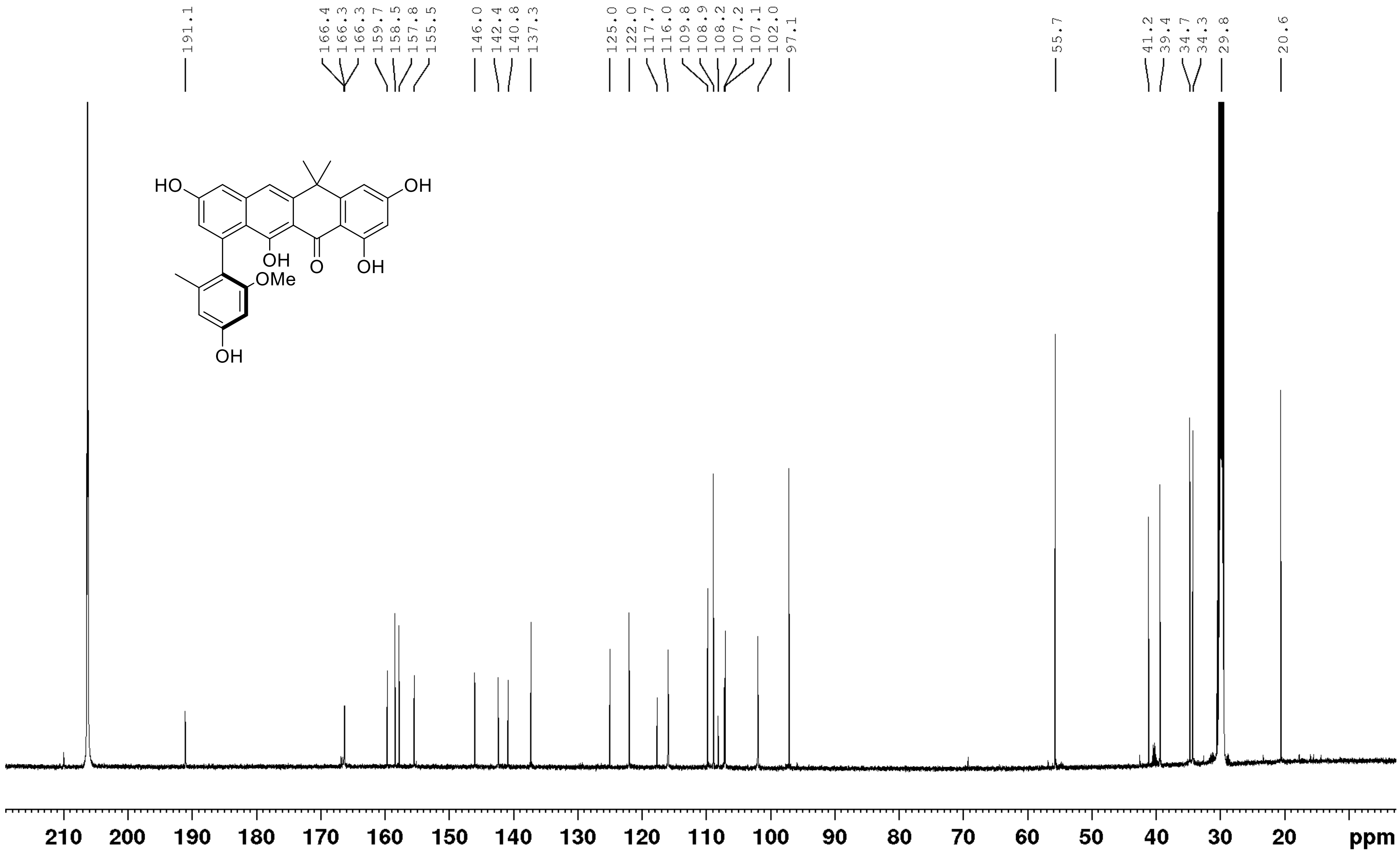

Figure S4. ^{13}C NMR (151 MHz) of fasamycin C (**17**) in acetone-d_6 at 298 K.

3. Comparison of published NMR data for napthacemycin B_4 with NMR of fasamycin D (18)

Table S7. Comparison of ^1H NMR data for napthacemycin B_4 (Yuan *et al*. 2020)[4]* and fasamycin D (**18**) (Qin *et al*. 2017)[5] in methanol-d_4

	**Naphthacemycin B_4** Yuan *et al.*[4]	**Fasamycin D (18)** Qin *et al.*[5]	
Position	δ ^1H (methanol-d_4, 400 MHz)	δ ^1H (methanol-d_4, 400 MHz)	Δδ ^1H (ppm)
1	-	-	-
2	6.35	6.33	0.02
3	-	-	-
4	6.35	6.33	0.02
5	-	-	-
6	-	-	-
7	-	-	-
8	-	-	-
9	-	-	-
10	-	-	-
11	-	-	-
12	-	-	-
13	-	-	-
14	6.22	6.21	0.01
15	-	-	-
16	6.68	6.68	-
17	-	-	-
18	-	-	-
19	-	-	-
20	7.88	7.87	0.01
21	-	-	-
22	-	-	-
23	-	-	-
24	6.79	6.77	0.02
25	1.9	1.89	0.01
26	1.75	1.74	0.01
27	1.75	1.74	0.01
28	3.59	3.58	0.01

* note that napththacemycin B_4 was first reported by Fukumoto *et al*.[2] However, the original NMR was recorded in acetone-d_6. As we no longer have any fasamycin D, comparison was made between our published NMR[5] for fasamycin D in methanol-d_4 to the later NMR for napthacemycin B_4 reported by Yuan *et al*.[4] in the same solvent.

Table S8. Comparison of ^{13}C NMR data for napthacemycin B_4 (Yuan *et al*)[4] and fasamycin D (**18**) (Qin *et al*)[5] in methanol-d_4

	**Naphthacemycin B_4** Yuan *et al.*[4]	**Fasamycin D (18)** Qin *et al.*[5]	
Position	δ ^{13}C (methanol-d_4, 100 MHz)	δ ^{13}C (methanol-d_4, 100 MHz)	Δδ ^{13}C (ppm)
1	136.3	138	-1.7
2	107.8	109.5	-1.7
3	156.6	158.3	-1.7
4	95.9	97.6	-1.7
5	157.3	156	1.3
6	123.9	125.5	-1.6
7	137.9	138.7	-0.8
8	117.4	119.1	-1.7
9	165.1	167	-1.9
10	106.5	108.1	-1.6
11	190.2	191.9	-1.7
12	107.1	108.8	-1.7
13	165.4	166.7	-1.3
14	100.7	102.4	-1.7
15	165.4	167.1	-1.7
16	105.9	107.4	-1.5
17	154.3	155.7	-1.4
18	38.7	40.3	-1.6
19	146.6	148.3	-1.7
20	110.7	112.3	-1.6
21	137.1	139.6	-2.5
22	112.3	114	-1.7
23	154.2	159	-4.8
24	120.4	122	-1.6
25	19.2	20.7	-1.5
26	33.5	35.1	-1.6
27	33.5	35.1	-1.6
28	54.5	56.2	-1.7

1. Note that C-5/C-23 have been assigned the opposite way around. Allowing for this gives Δδ ^{13}C -1.8 ppm (C-5) and -1.7 ppm (C-23).
2. Note that C-7/C-21 have been assigned the opposite way around. Allowing for this gives Δδ ^{13}C -1.6 ppm (C-7) and -1.7 ppm (C-21).
3. Note that after allowances all shifts have Δδ ^{13}C of about -1.6 ppm. This is likely due to a difference in calibrating the spectra.

4. Comparison of published NMR for napthacemycin B_7 with NMR of fasamycin E (7)

Table S9. Comparison of ^1H NMR data for napthacemycin B_7 (Huo *et al.*)[3] and fasamycin E in acetone-d_6

	**Naphthacemycin B_7** Huo *et al.*[3]	**Fasamycin E* (7)** This study	
Position	δ ^1H (acetone-d_6, 500 MHz)	δ ^1H (acetone-d_6, 600 MHz)	Δδ ^1H (ppm)
1	-	-	-
2	-	-	-
3	-	-	-
3-OH	not seen	not seen	-
4	6.63	6.62	0.01
5	-	-	-
6	-	-	-
7	-	-	-
8	-	-	-
9	-	-	-
9-OH	14.54	14.55	-0.01
10	6.32	6.31	0.01
11	-	-	-
12	-	-	-
13	-	-	-
13-OH	12.69	12.71	-0.02
14	-	-	-
15	-	-	-
15-OH	not seen	not seen	-
16	6.82	6.81	0.01
17	-	-	-
18	-	-	-
19	-	-	-
20	7.93	7.93	0.00
21	-	-	-
22	-	-	-
23	-	-	-
23-OH	not seen	not seen	-
24	6.95	6.95	0.00
25	2.02	2.00	0.02
26	1.79	1.78	0.01
27	1.80	1.79	0.01
28	3.59	3.58	0.01

* note a doubling of several signals (Figure S5)

Table S10. Comparison of ^{13}C NMR data for napthacemycin B_7 (Huo *et al.* 2020)[3] and fasamycin E (**7**) in acetone-d_6

	**Naphthacemycin B_7** Huo *et al.*[3]	**Fasamycin E* (7)** This study	
Position	δ ^{13}C (acetone-d_6, 151 MHz)	δ ^{13}C (acetone-d_6, 151 MHz)	Δδ ^{13}C (ppm)
1	134.1	134.1	0.0
2	112.0	112.0	0.0
3	152.5	152.5	0.0
4	97.5	97.5	0.0
5	155.8	155.7	0.1
6	125.1	125.0	0.1
7	137.6	137.5	0.1
8	117.6	117.4	0.2
9	165.4	165.4	0.0
10	107.0	106.9	0.1
11	190.3	190.3	0.0
12	107.4	107.3	0.1
13	165.9	165.9	0.0
14	101.3	101.2	0.1
15	165.5	165.6	-0.1
16	106.4	106.4	0.0
17	154.7	154.7	0.0
18	39.0	39.0	0.0
19	147.2	147.1	0.1
20	110.9	110.9	0.0
21	137.2	137.2	0.0
22	112.9	112.8	0.1
23	154.1	154.2	-0.1
24	120.7	120.7	0.0
25	17.4	17.4	0.0
26	33.6	33.6	0.0
27	34.0	34.1	-0.1
28	55.1	55.1	0.0

* note a splitting of several signals (Figure S6)

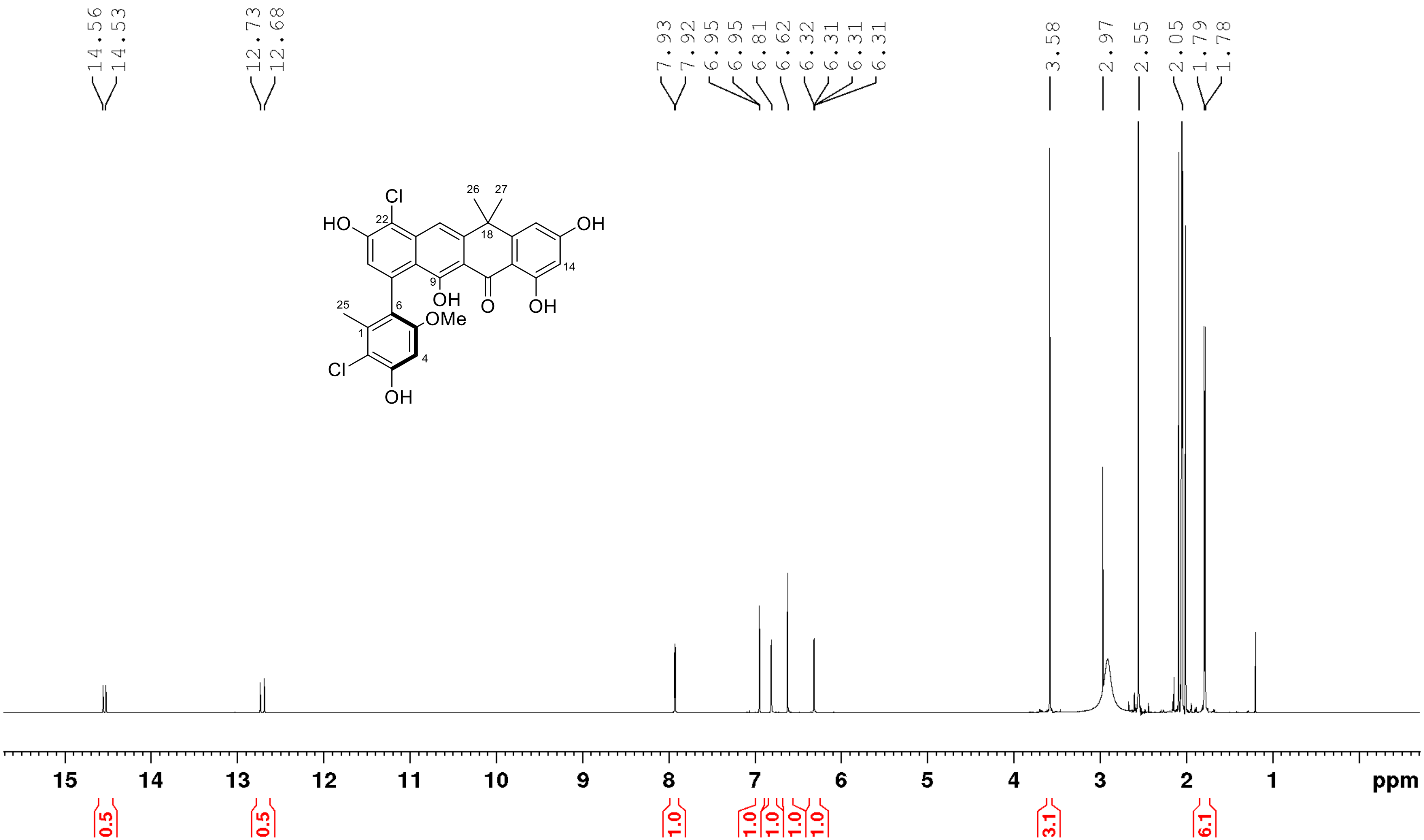

Figure S5. ^{1}H NMR (600 MHz) of fasamycin E (**7**) in acetone-d_6 at 298 K.

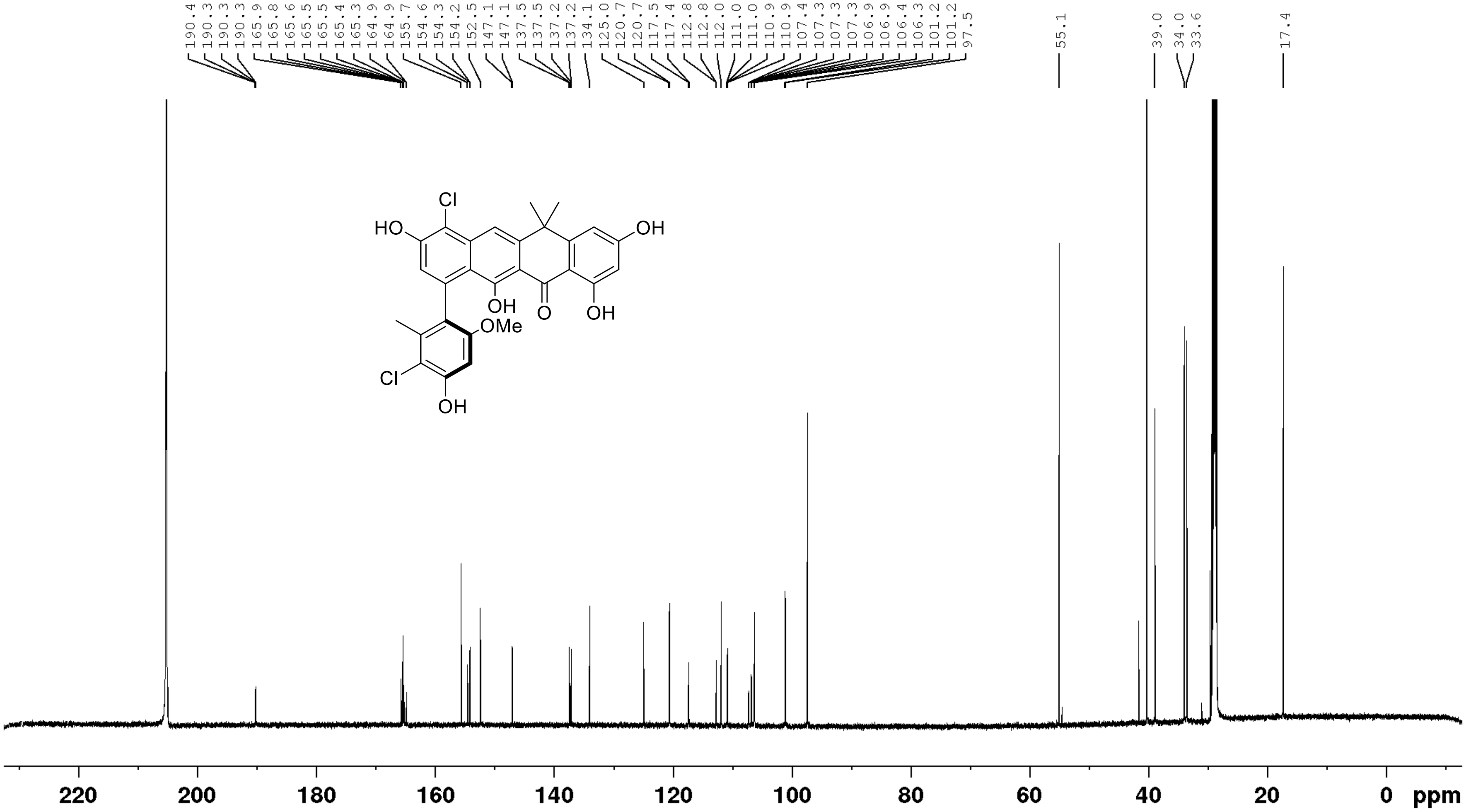

Figure S6. ^{13}C NMR (151 MHz) of fasamycin E (**7**) in acetone-d_6 at 298 K.

5. Comparison of published NMR data for napthacemycin B_{11} with NMR of fasamycin L (8)

Table S11. Comparison of ^1H NMR data for napthacemycin B_{11} (Huo *et al.*)[3] and fasamycin L (**8**) in $CDCl_3$

	**Naphthacemycin B_{11}** Huo *et al.*[3]	**Fasamycin L (8)** This study	
Position	δ ^1H ($CDCl_3$, 500 MHz)	δ ^1H ($CDCl_3$, 600 MHz)	Δδ ^1H (ppm)
1	-	-	-
2	-	-	-
3	-	-	-
3-OH	not seen	5.99	not applicable
4	6.57	6.56	0.01
5	-	-	-
6	-	-	-
7	-	-	-
8	-	-	-
9			-
9-OH	14.2	14.22	-0.02
10	-	-	-
11	-	-	-
12	-	-	-
13	-	-	-
13-OH	13.6	13.6	0.00
14	-	-	-
15	-	-	-
15-OH	not seen	not seen	-
16	6.87	6.86	0.01
17	-	-	-
18	-	-	-
19	-	-	-
20	7.75	7.77	-0.02
21	-	-	-
22	-	-	-
23	-	-	-
23-OH	not seen	7.66	not applicable
24	6.92	6.90	0.02
25	2.02	2.01	0.01
26	1.76	1.75	0.01
27	1.78	1.76	0.02
28	3.63	3.62	0.01

Table S12. Comparison of ^{13}C NMR data for napthacemycin B_{11} (Huo *et al.*)[3] and fasamycin L (**8**) in $CDCl_3$

	**Naphthacemycin B_{11}** Huo *et al.*[3]	**Fasamycin L (8)** This study	
Position	δ ^{13}C ($CDCl_3$, 151 MHz)	δ ^{13}C ($CDCl_3$, 151 MHz)	Δδ ^{13}C (ppm)
1	134.2	134.1	0.1
2	112.0	111.9	0.1
3	151.2	151.1	0.1
4	96.9	96.8	0.1
5	156.1	155.9	0.2
6	125.5	125.5	0.0
7	138.0	137.5	0.5
8	118.2	117.8	0.4
9	165.9	165.7	0.2
10	107.5	107.2	0.3
11	190.4	190.1	0.3
12	105.9	105.9	0.0
13	160.2	160.2	0.0
14	109.1	108.6	0.5
15	158.1	158.8	-0.7
16	105.5	105.7	-0.2
17	152.3	151.9	0.4
18	39.3	39.0	0.3
19	147.0	146.7	0.3
20'	111.4	111.2	0.2
21	136.7	136.8	-0.1
22	113.0	113.2	-0.2
23	152.8	153.1	-0.3
24	120.1	120.2	-0.1
25	18.2	18.0	0.2
26	34.5	34.3	0.2
27	34.7	34.5	0.2
28	56.2	56.0	0.2

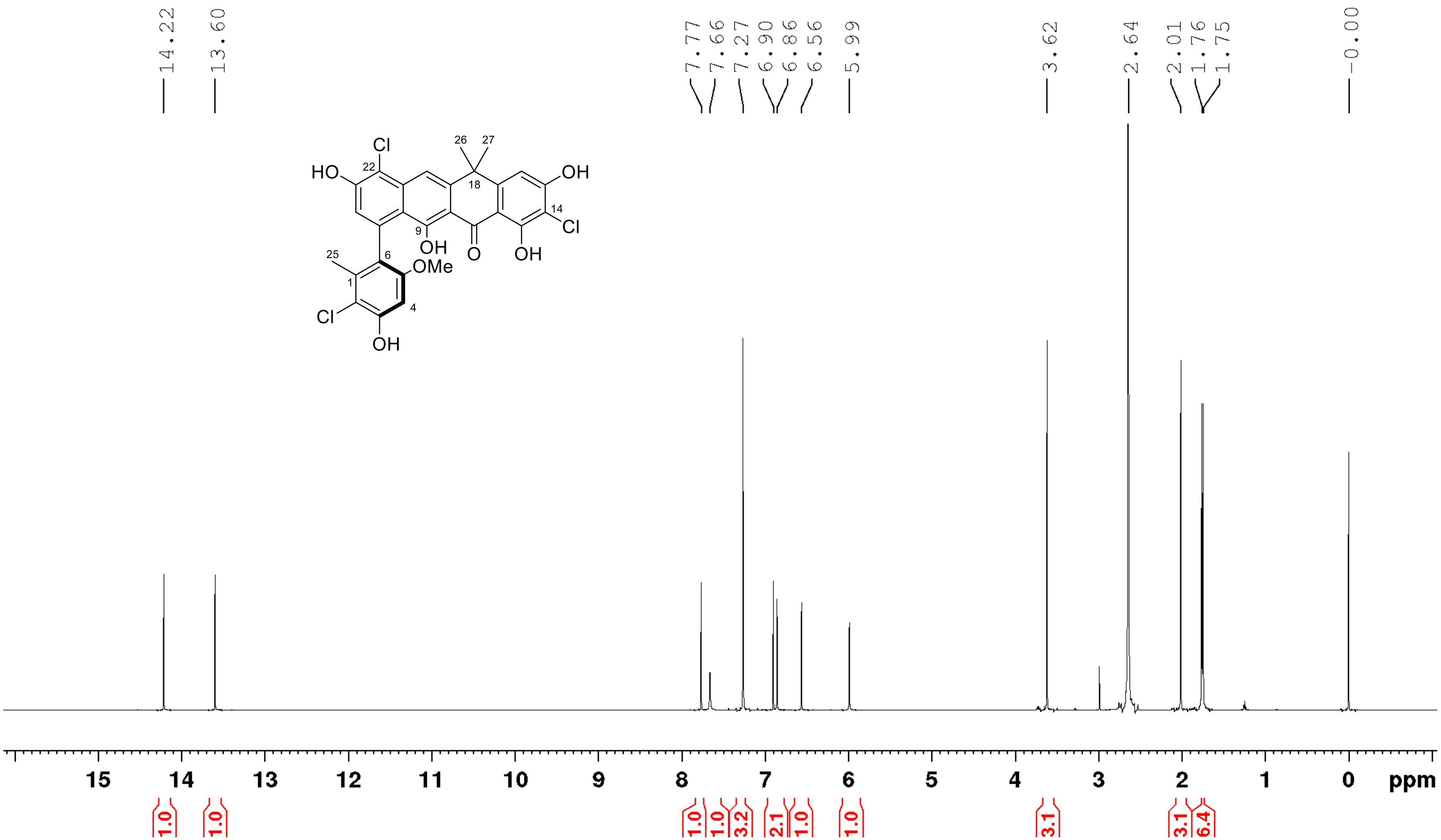

Figure S7. ^{1}H NMR (600 MHz) of fasamycin L (**8**) in $CDCl_3$ at 298 K.

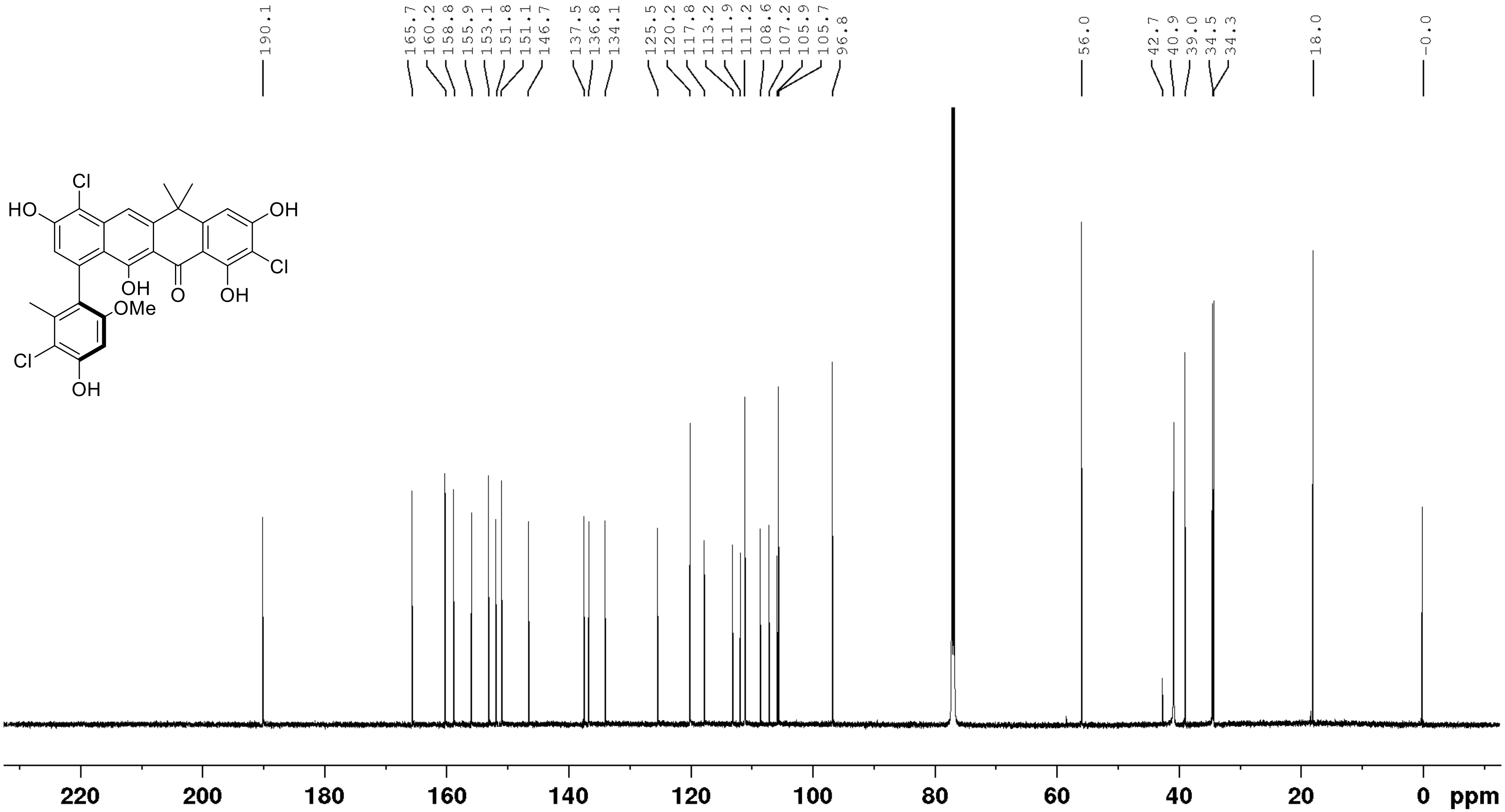

Figure 8. ^{13}C NMR (151 MHz) of fasamycin L (**8**) in $CDCl_3$ at 298 K.

6. Comparison of published NMR data for napthacemycin B_{12} with NMR of fasamycin Q (21)

Table S13. Comparison of ^1H NMR data for napthacemycin B_{12} (Huo *et al.*)[3] and fasamycin Q (**21**) in $CDCl_3$

	**Naphthacemycin B_{12}** Huo *et al.*[3]	**Fasamycin Q (21)** This study	
Position	δ ^1H ($CDCl_3$, 500 MHz)	δ ^1H ($CDCl_3$, 600 MHz)	Δδ ^1H (ppm)
1	-	-	-
2	-	-	-
3	-	-	-
4	6.57	6.57	0.00
4-OH	14.28	14.28	0.00
5	-	-	-
5-OMe	3.63	3.63	0.00
6	-	-	-
6-OH	14.17	14.17	0.00
7	-	-	-
8	-	-	-
9	-	-	-
10	-	-	-
11	-	-	-
12	-	-	-
13	-	-	-
14	-	-	-
14	6.92	6.91	-0.01
15	-	-	-
16	-	-	-
17	-	-	-
18	-	-	-
19	-	-	-
20	7.72	7.72	0.00
21	-	-	-
22	-	-	-
23	-	-	-
25	2.02	2.02	0.00
26	2.07	2.07	0.00
27	2.09	2.09	0.00

Table S14. Comparison of ^{13}C NMR data for napthacemycin B_{12} (Huo *et al*)[3] and fasamycin Q (**21**) in $CDCl_3$

	**Naphthacemycin B_{12}** Huo *et al.*[3]	**Fasamycin Q (21)** This study	
	δ ^{13}C ($CDCl_3$, 125 MHz)	δ ^{1}H ($CDCl_3$, 125 MHz)	Δδ 1H (ppm)
1	134.1	134.0	-0.1
2	112.0	111.9	-0.1
3	151.2	151.1	-0.1
4	96.9	96.7	-0.2
5	156.1	155.9	-0.2
5-OMe	56.1	56.0	-0.1
6	125.4	125.2	-0.2
7	138.1	137.9	-0.2
8	117.7	117.6	-0.1
9	165.6	165.4	-0.2
10	106.2	106.0	-0.2
11	190.4	190.2	-0.2
12	107.9	107.8	-0.1
13	159.9	159.8	-0.1
14	109.4	109.2	-0.2
14	120.1	120.0	-0.1
15	155.1	155.0	-0.1
16	111.4	111.2	-0.2
17	146.6	146.4	-0.2
18	40.0	39.9	-0.1
19	149.5	149.3	-0.2
20	112.2	112.1	-0.1
21	137.2	137.1	-0.1
22	113.1	112.9	-0.2
23	152.9	152.8	-0.1
25	18.2	18.0	-0.2
26	30.3	30.1	-0.2
27	30.3	30.2	-0.1

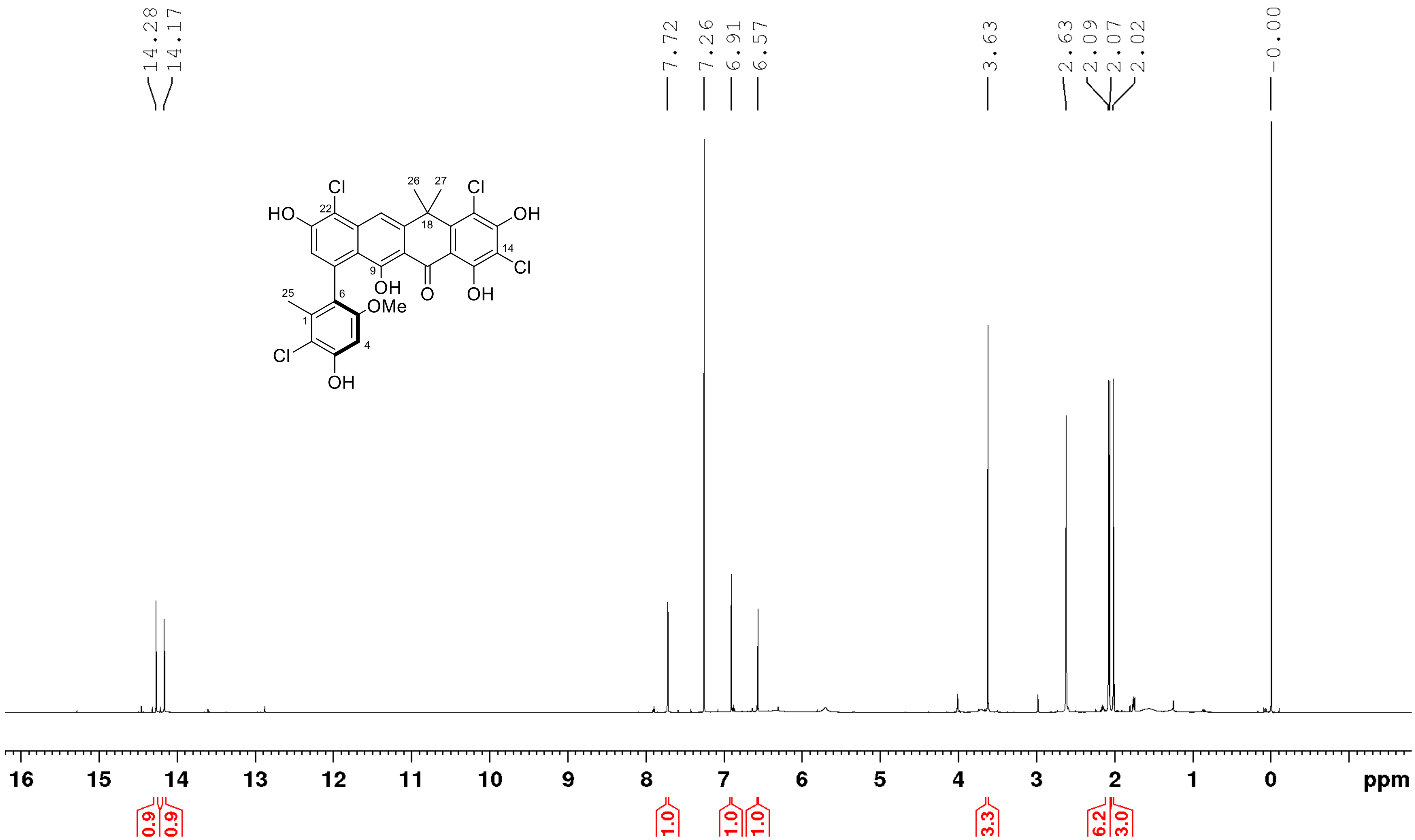

Figure S9. ^{1}H NMR (600 MHz) of fasamycin Q (**21**) in $CDCl_3$ at 298 K.

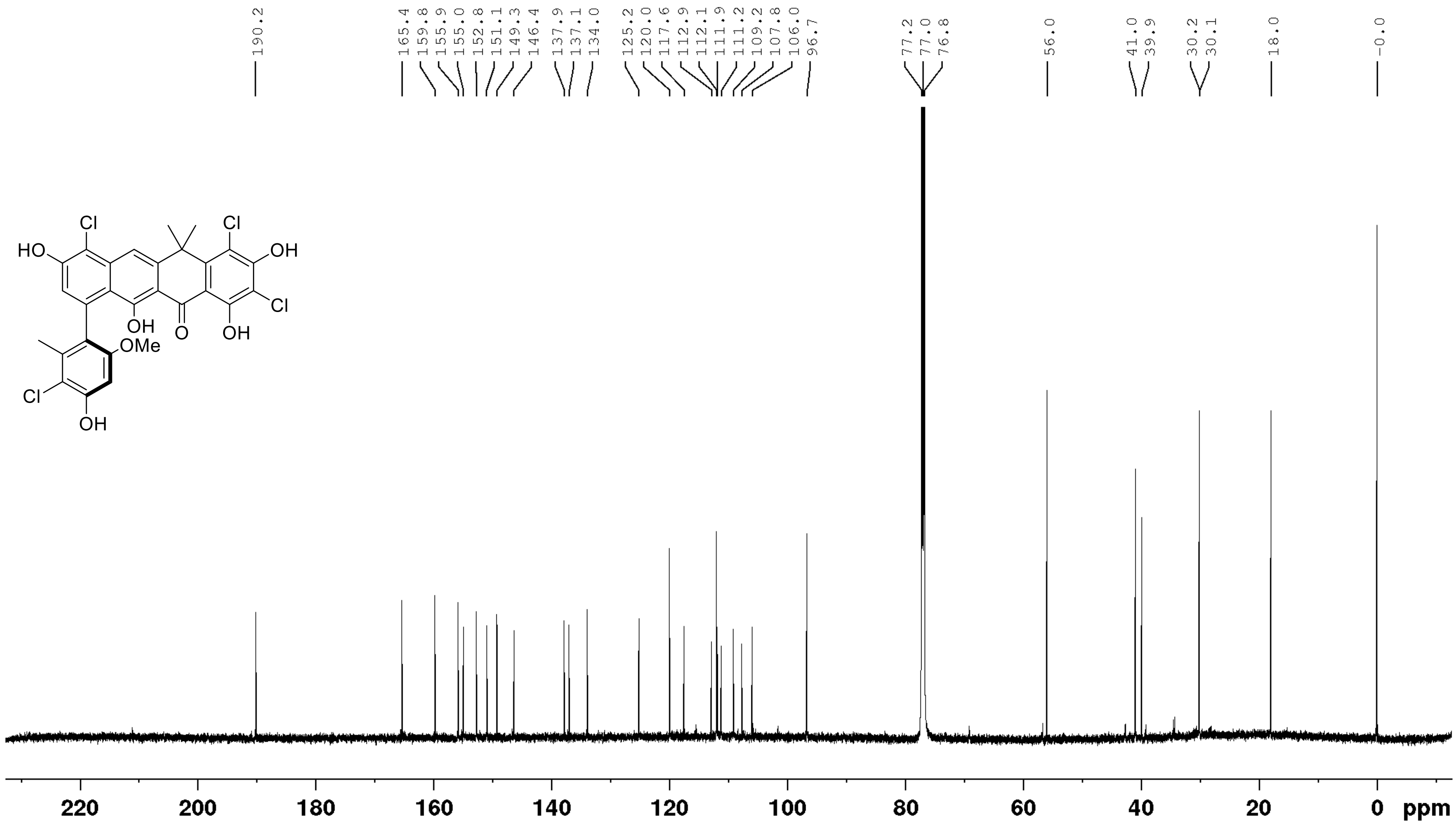

Figure S10. ^{13}C NMR (151 MHz) of fasamycin Q (**21**) in $CDCl_3$ at 298 K.

7. High-resolution electrospray ionisation mass spectrometry (HRESIMS) of lactone intermediate F (25)

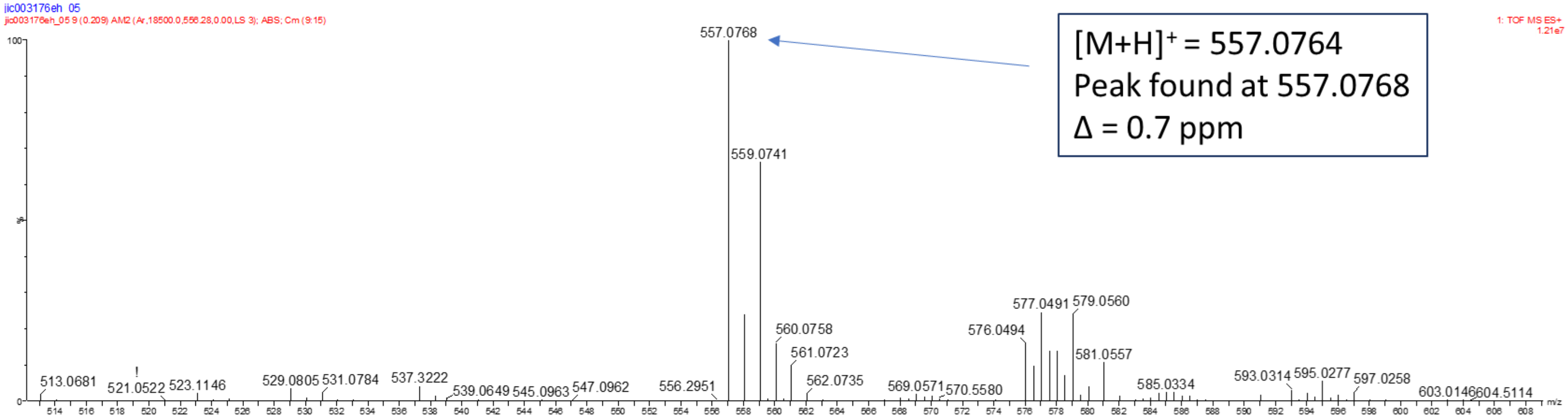

Figure S11. HRESIMS of lactone intermediate F (**25**) showing an isotope pattern consistent with 2 chlorine atoms.

8. NMR spectra for lactone intermediate F (25)

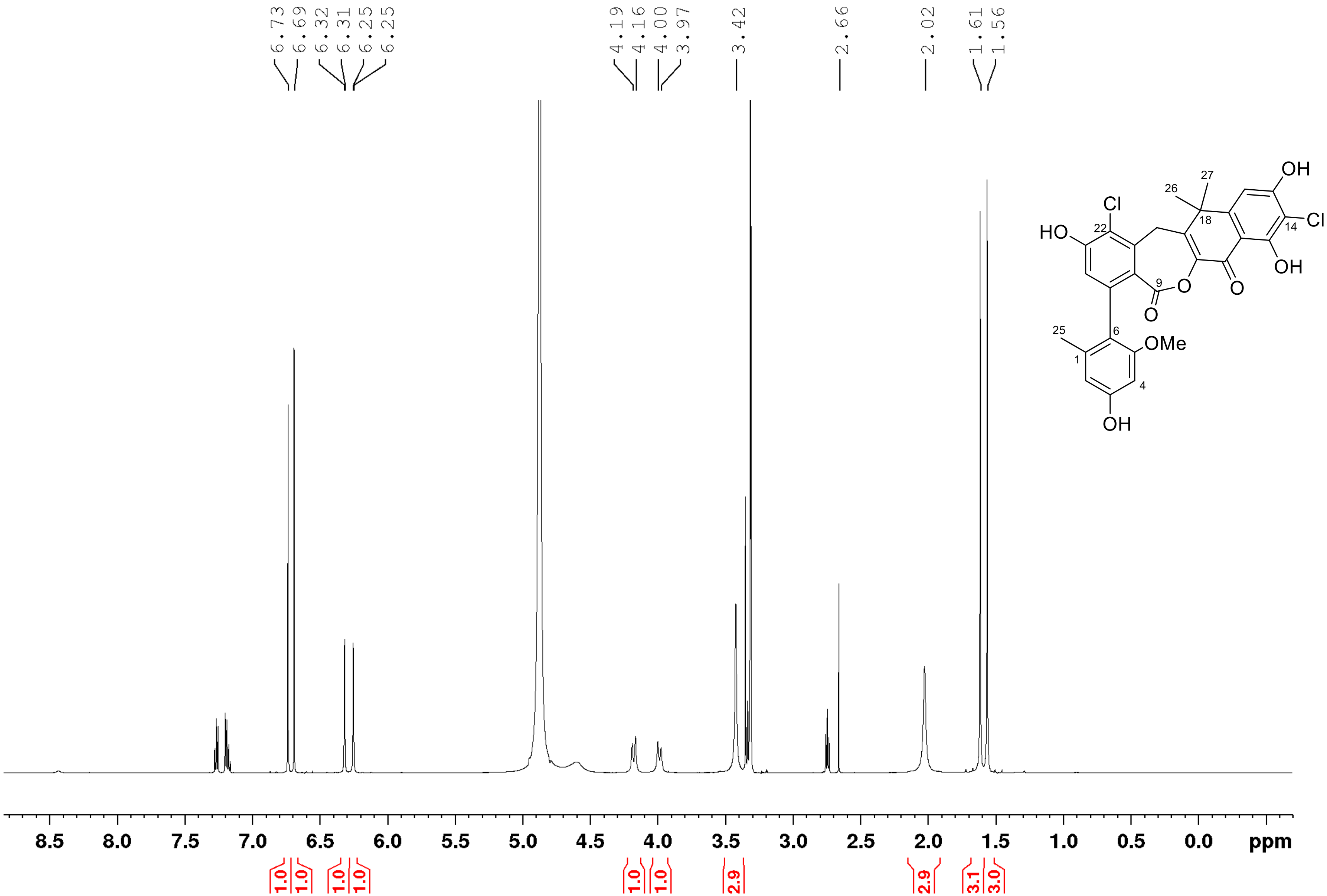

Figure S12. ^{1}H NMR for lactone intermediate F (**25**) (CD_3OD, 600 MHz, 298K).

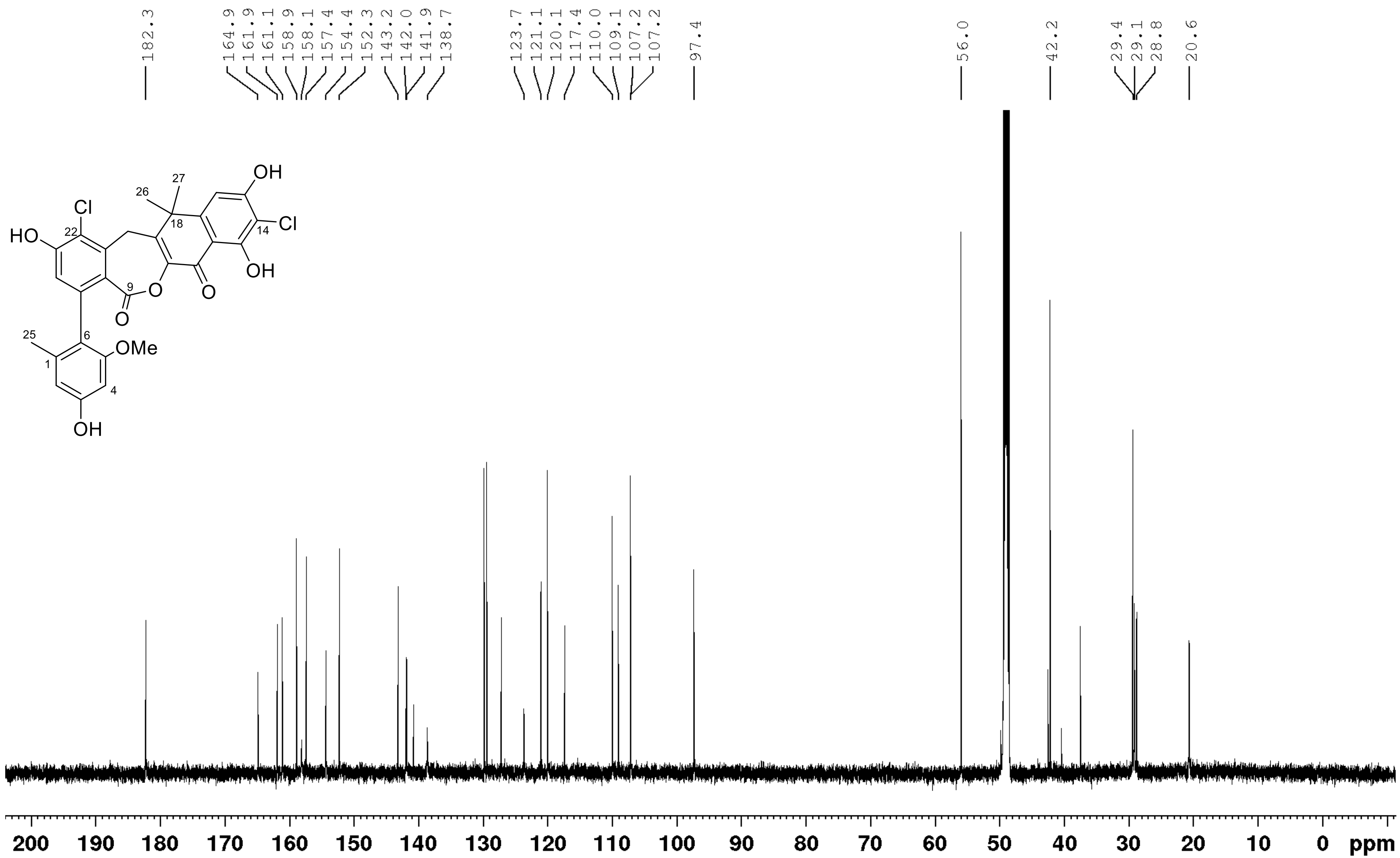

Figure S13. ^{13}C NMR for lactone intermediate F (**25**) (CD_3OD, 151 MHz, 298K).

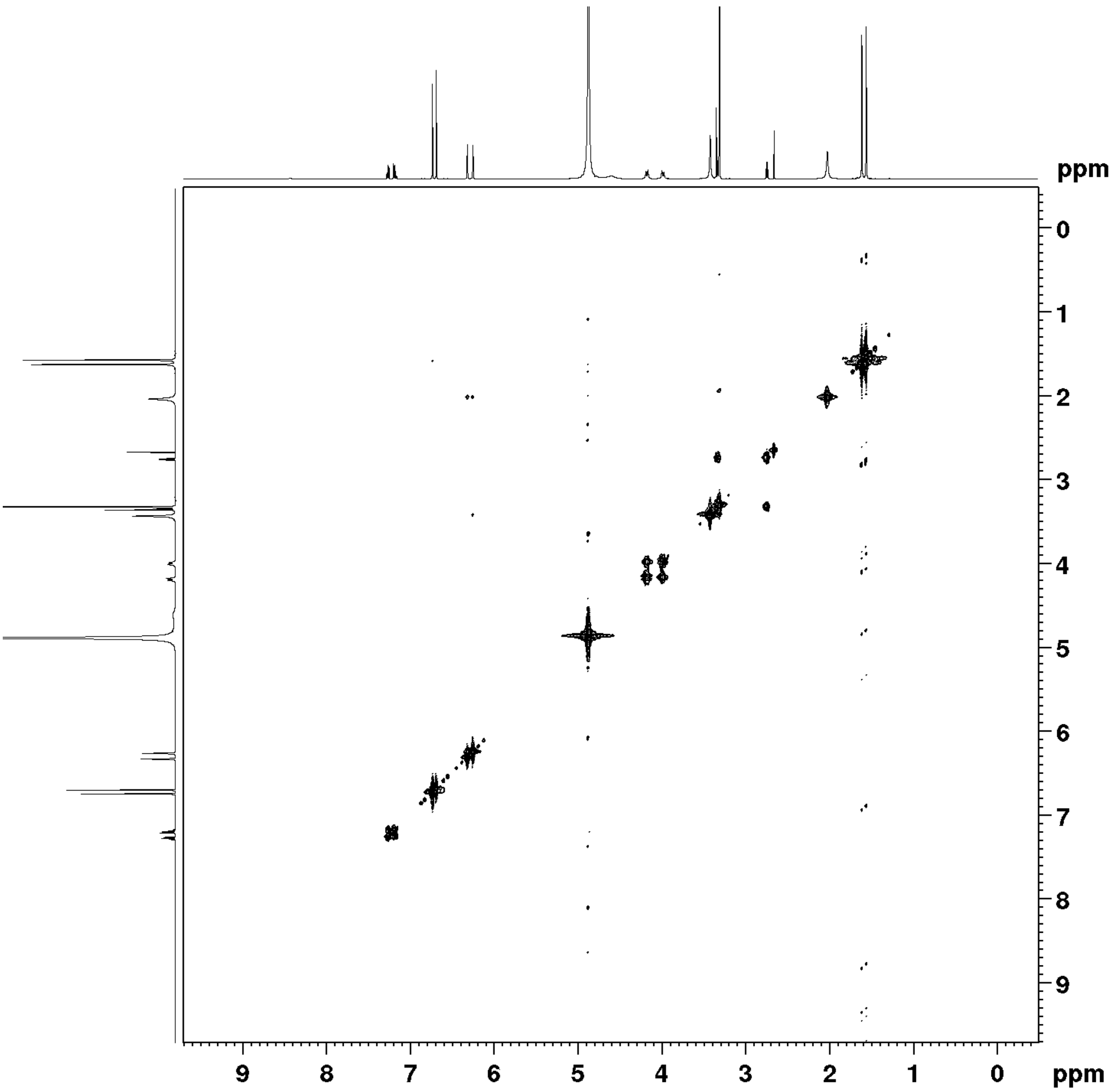

Figure S14. ^{1}H-^{1}H COSY NMR for lactone intermediate F (**25**) (CD_3OD, 298K).

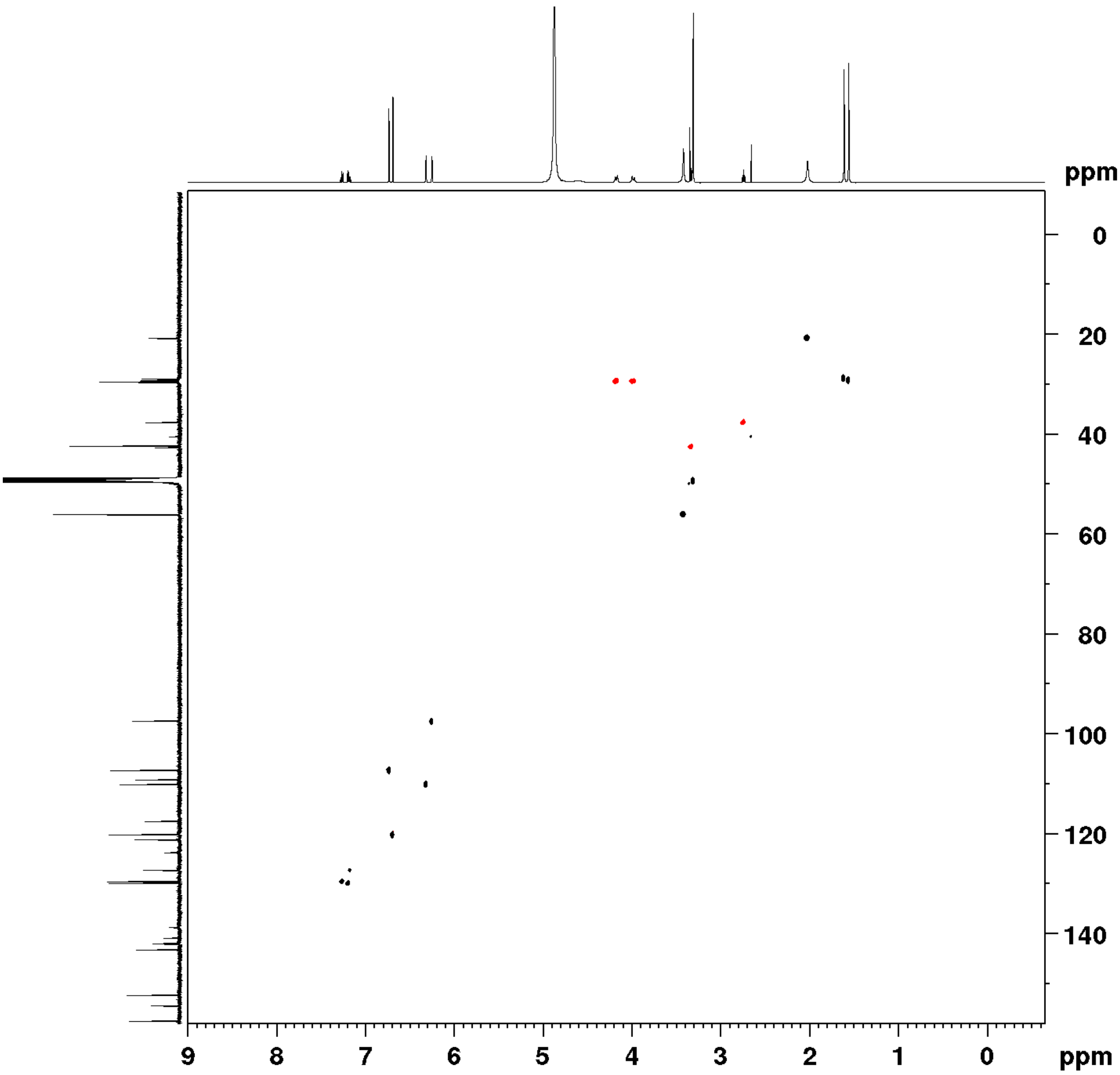

Figure S15. ^{1}H-^{13}C HSQC-edited NMR for lactone intermediate F (**25**) (CD_3OD, 298K).

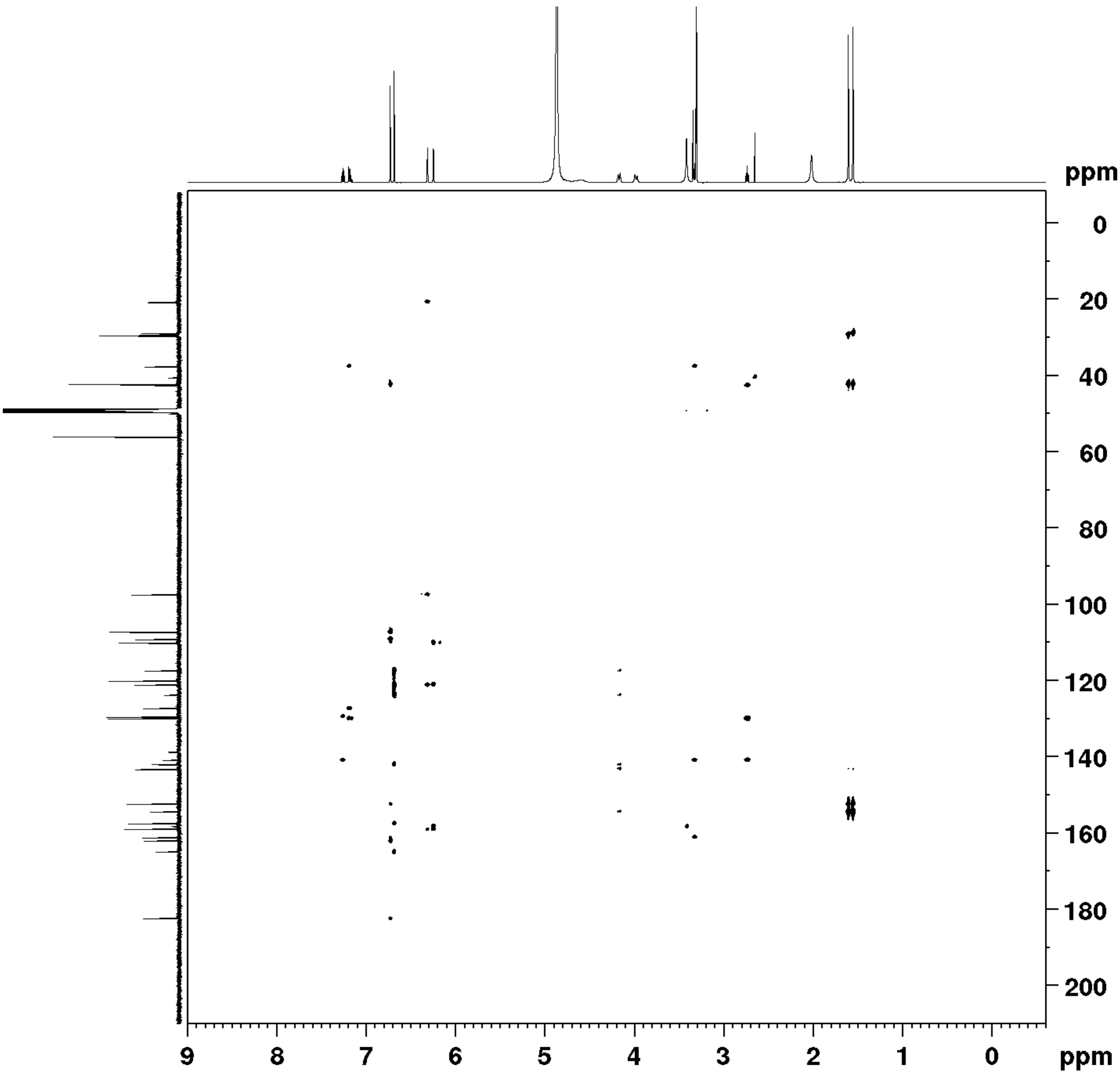

Figure S16. ^{1}H-^{13}C HMBC NMR for lactone intermediate F (**25**) (CD_3OD, 298K).

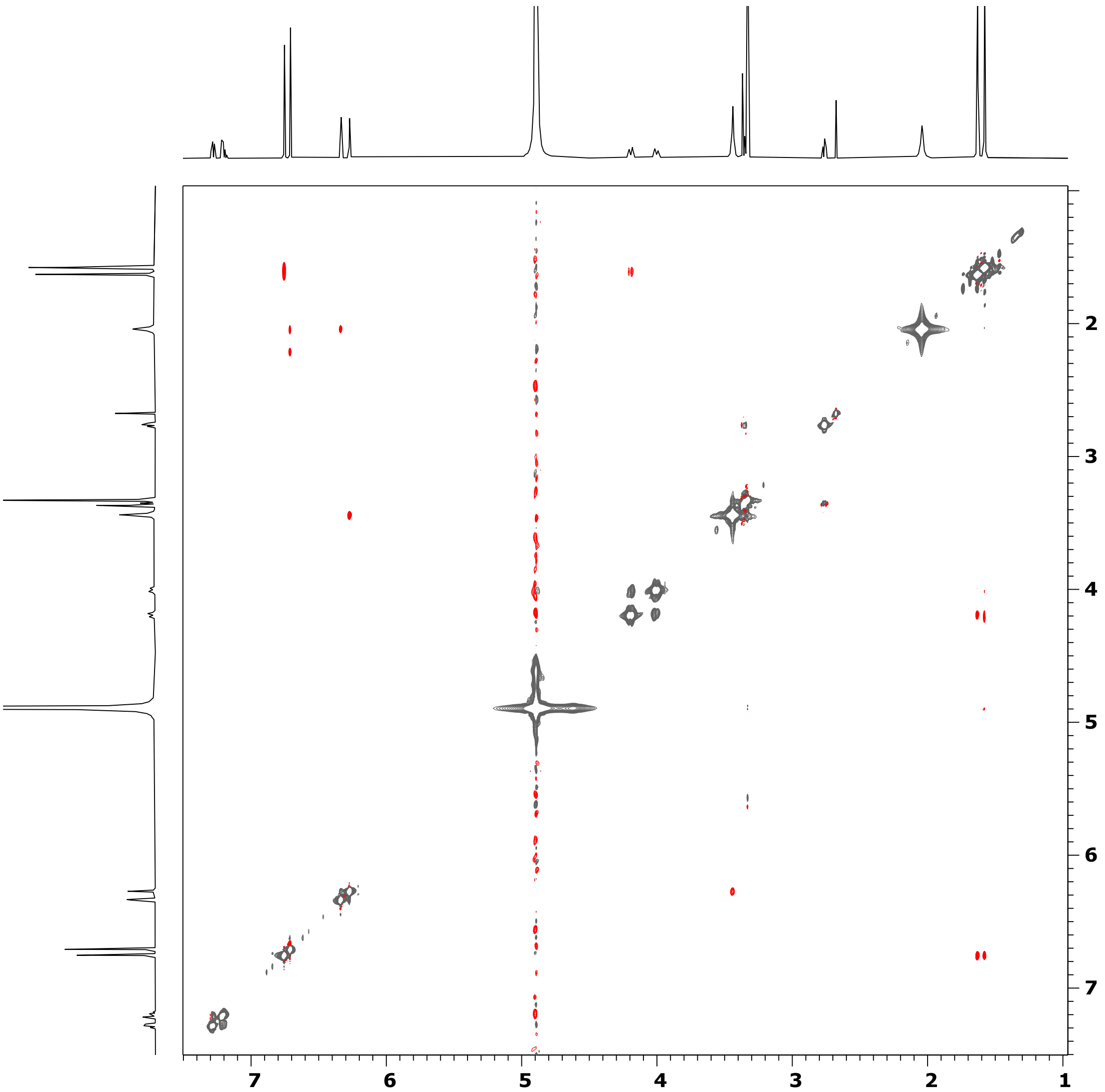

Figure S17. ¹H-¹H NOESY NMR for lactone intermediate F (**25**) (CD_3OD, 298K).

9. Re-examining the published NOESY NMR of fasamycin C (17)

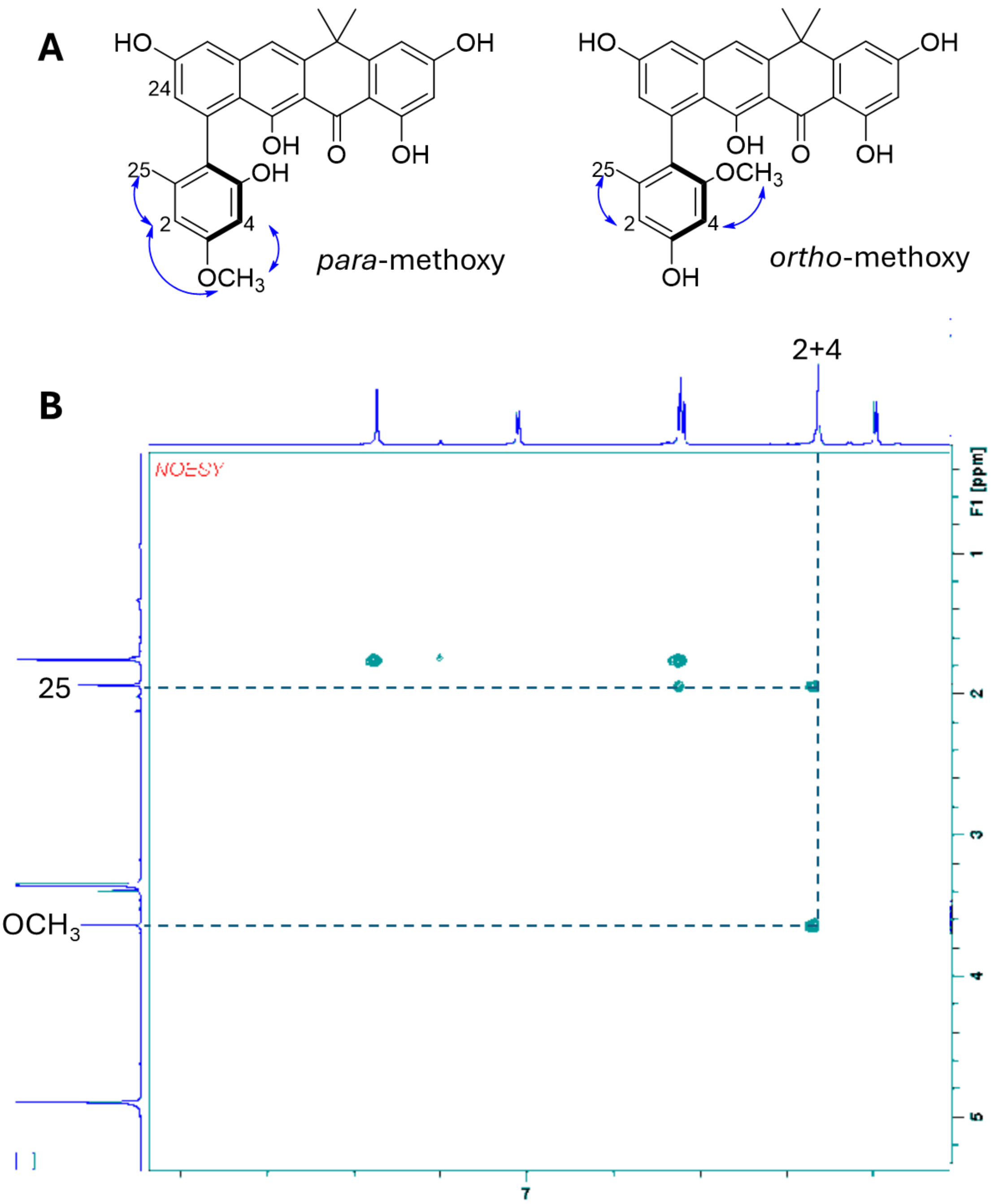

Figure S18. Re-examining the original published NOESY NMR shows that of an assignment on the regiochemistry of the methoxy group on the hanging E ring of fasamycin C should not have been made based on this spectrum.[5] **A** Key expected NOESY crosspeaks for the *para*-methoxy and *ortho*-methoxy isomers of fasamycin C; **B** Published NOESY NMR for fasamycin C shows that H-2 and H-4 have the same chemical shift. As such the cross-peaks with H-25 and OCH_3 *cannot* be used to determine the regioisomers. Note the spectrum has been cropped and annotated for clarity. For the original please see Qin *et al.,* Chemical Science, 2017, **8**, 3218-3227; Supporting Information, Page 8.

https://www.rsc.org/suppdata/c6/sc/c6sc04265a/c6sc04265a1.pdf

10. 1,*n*-ADEQUATE NMR experiments

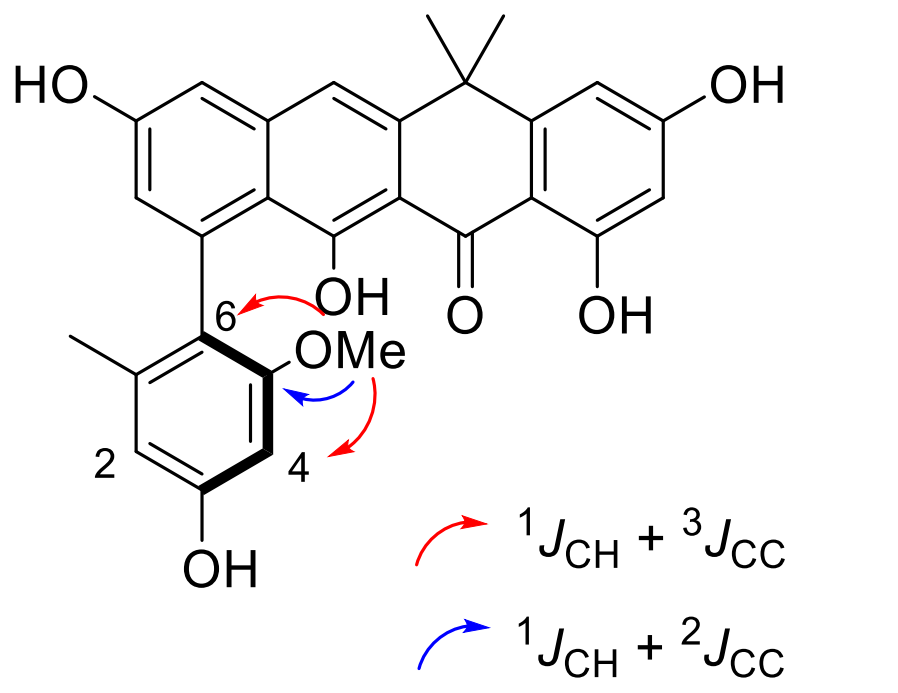

fasamycin C (17) / naphthacemycin B$_2$

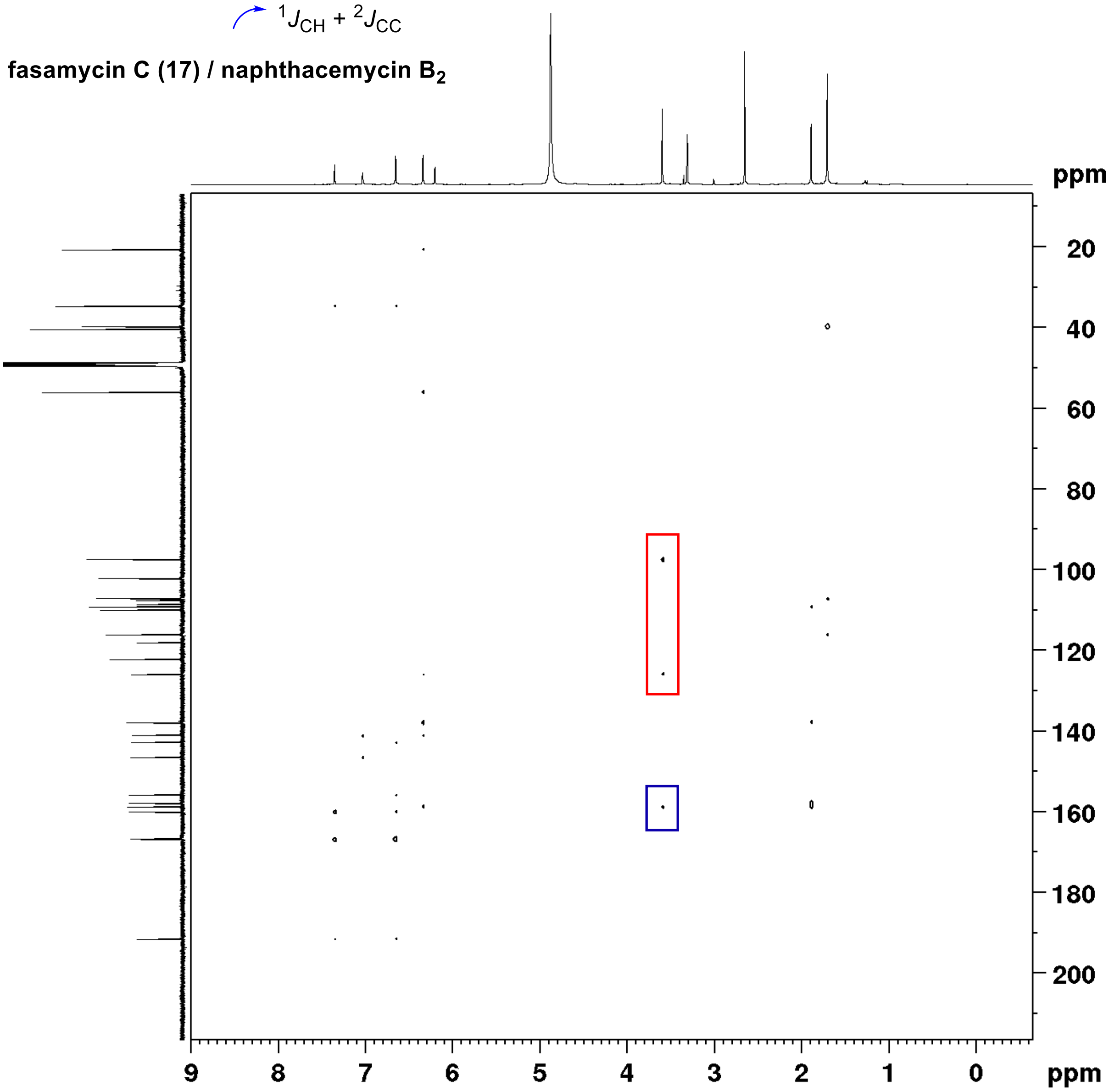

Figure S19. 1,*n*-ADEQUATE NMR in methanol-d_4 for fasamycin C (**17**) / naphthacemycin B$_2$ with a delay for the $^{n}J_{CC}$ coupling of 7 Hz. Pseudo $^{4}J_{CH}$ correlations are visible from the methoxy protons (δ_H 3.60) to C-4 (δ_C 97.4) and C-6 (δ_C 125.9) indicative of the *ortho*-methoxy regioisomer.

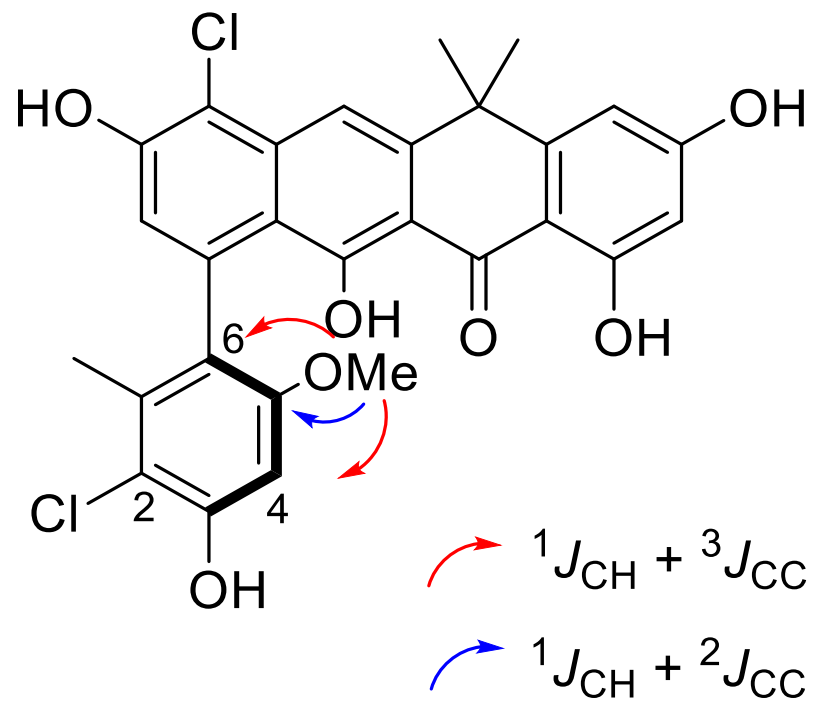

fasamycin E (7) / naphthacemycin B$_7$

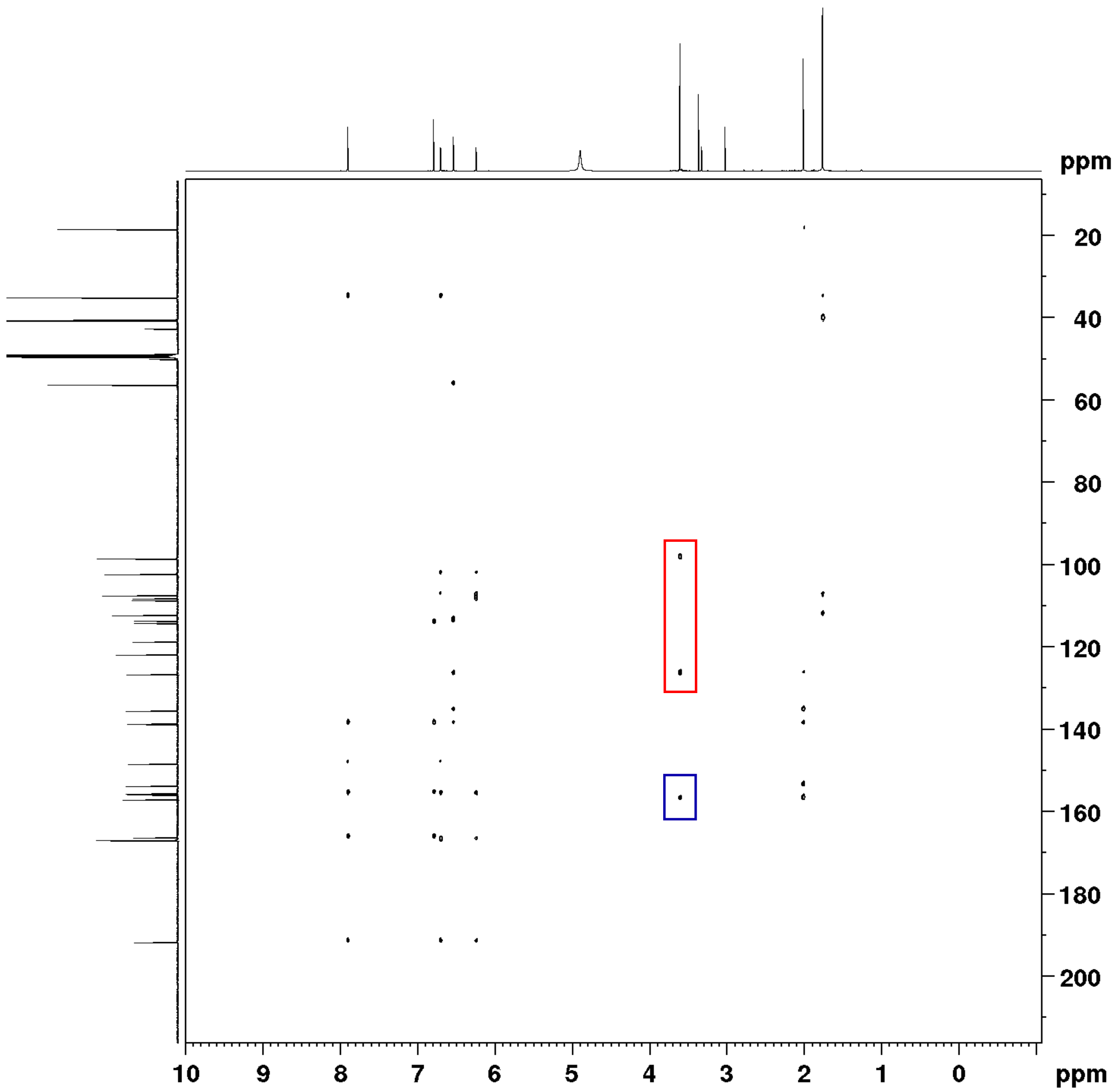

Figure S20. 1,*n*-ADEQUATE NMR in methanol-d_4 for fasamycin E (**7**) / naphthacemycin B$_7$ with a delay for the $^{n}J_{CC}$ coupling of 7 Hz. Pseudo $^{4}J_{CH}$ correlations are visible from the methoxy protons (δ_H 3.61) to C-4 (δ_C 98.4) and C-6 (δ_C 126.5) indicative of the *ortho*-methoxy regioisomer.

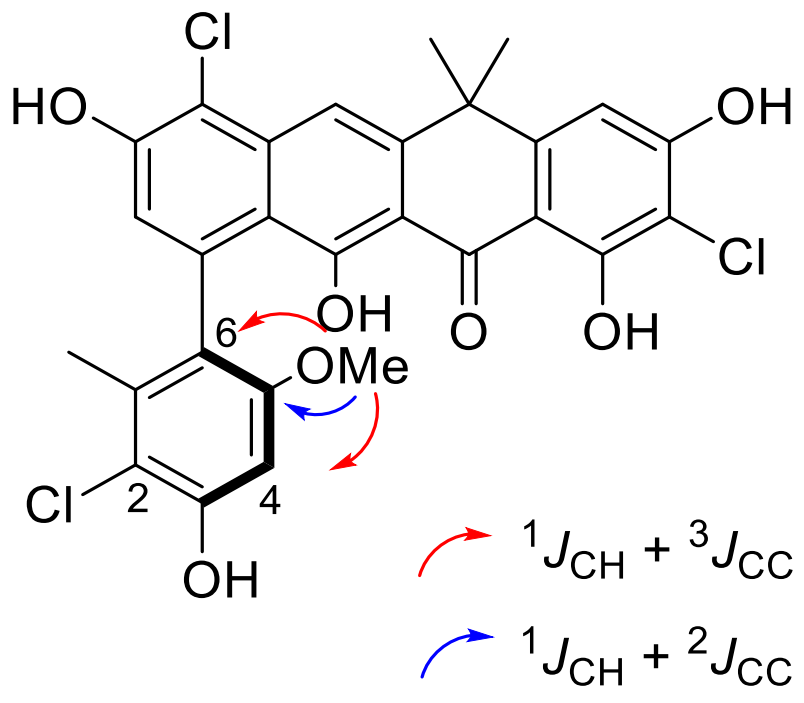

fasamycin L (8)

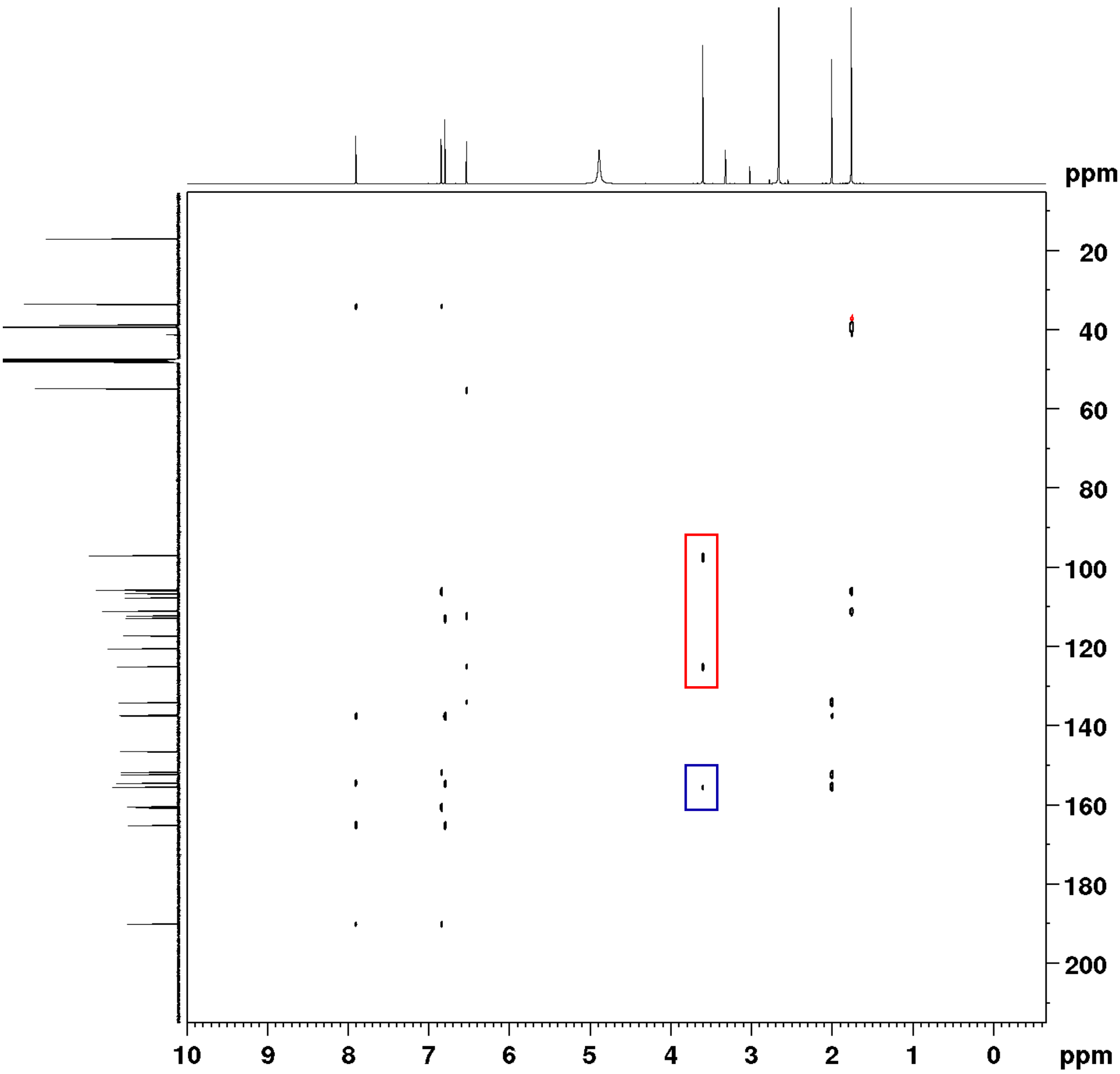

Figure S21. 1,*n*-ADEQUATE NMR in methanol-d_4 for fasamycin L (**8**) with a delay for the $^nJ_{CC}$ coupling of 7 Hz. Pseudo $^4J_{CH}$ correlations are visible from the methoxy protons (δ_H 3.61) to C-4 (δ_C 98.4) and C-6 (δ_C 126.3) indicative of the *ortho*-methoxy regioisomer.

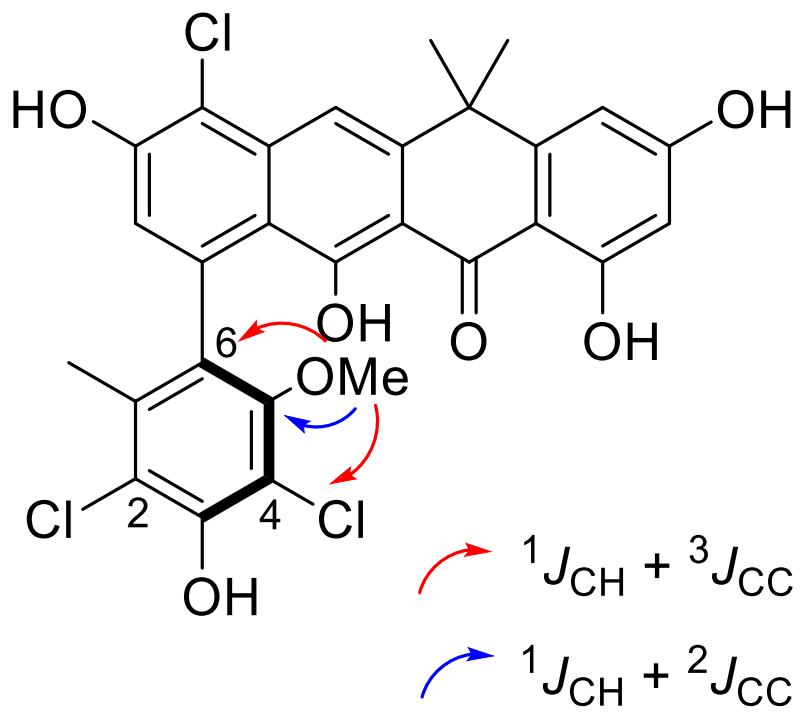

fasamycin M (9)

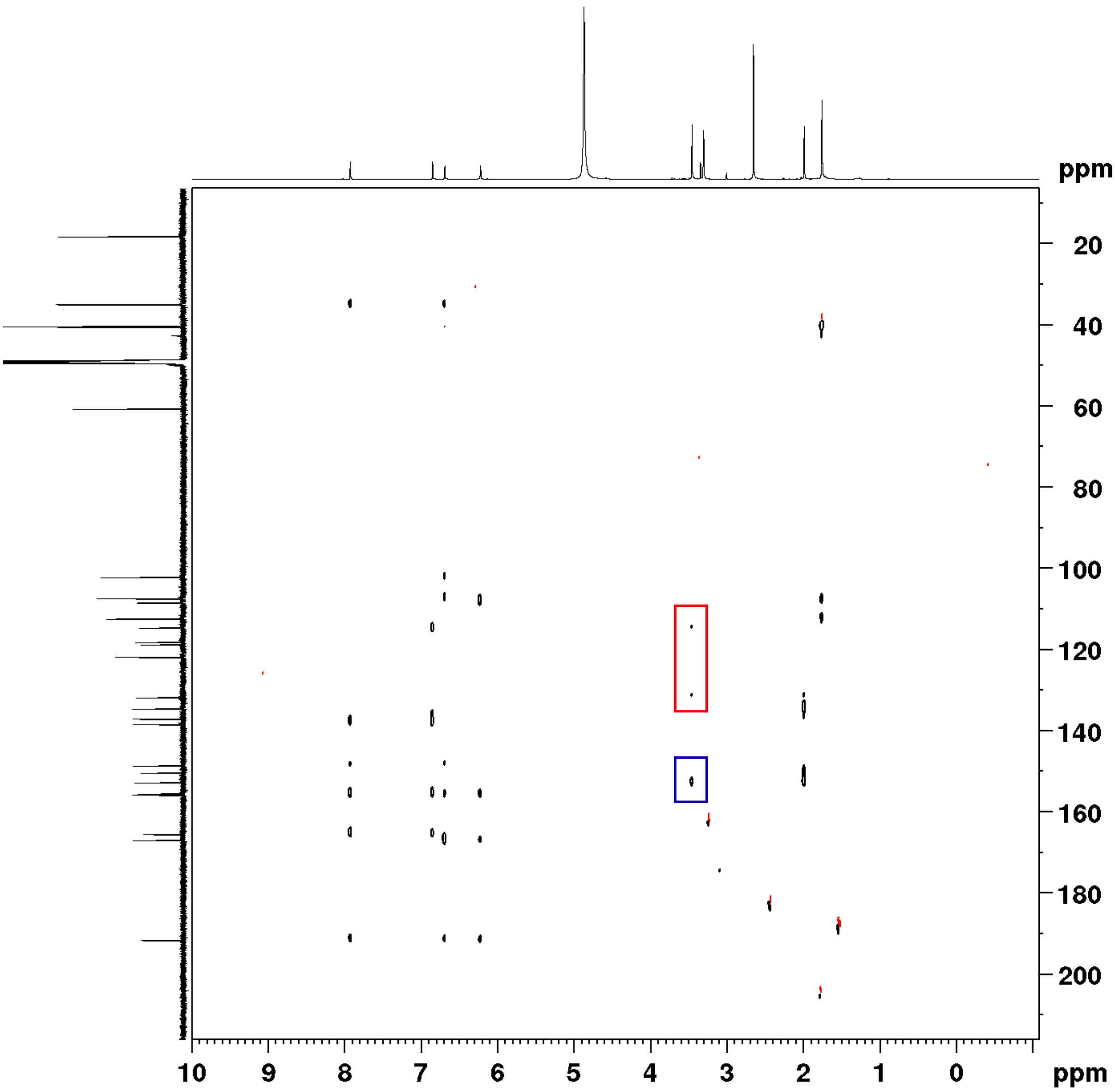

Figure S22. 1,*n*-ADEQUATE NMR in methanol-d_4 for fasamycin M (**9**) with a delay for the $^nJ_{CC}$ coupling of 7 Hz. Pseudo $^4J_{CH}$ correlations are visible from the methoxy protons (δ_H 3.46) to C-4 (δ_C 114.6) and C-6 (δ_C 131.8) indicative of the *ortho*-methoxy regioisomer.

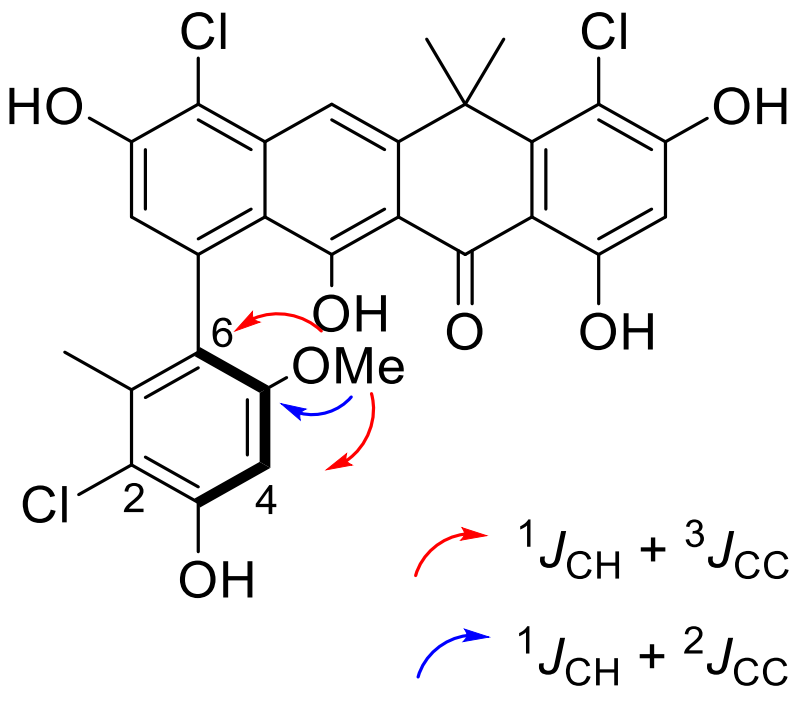

fasamycin N (20)

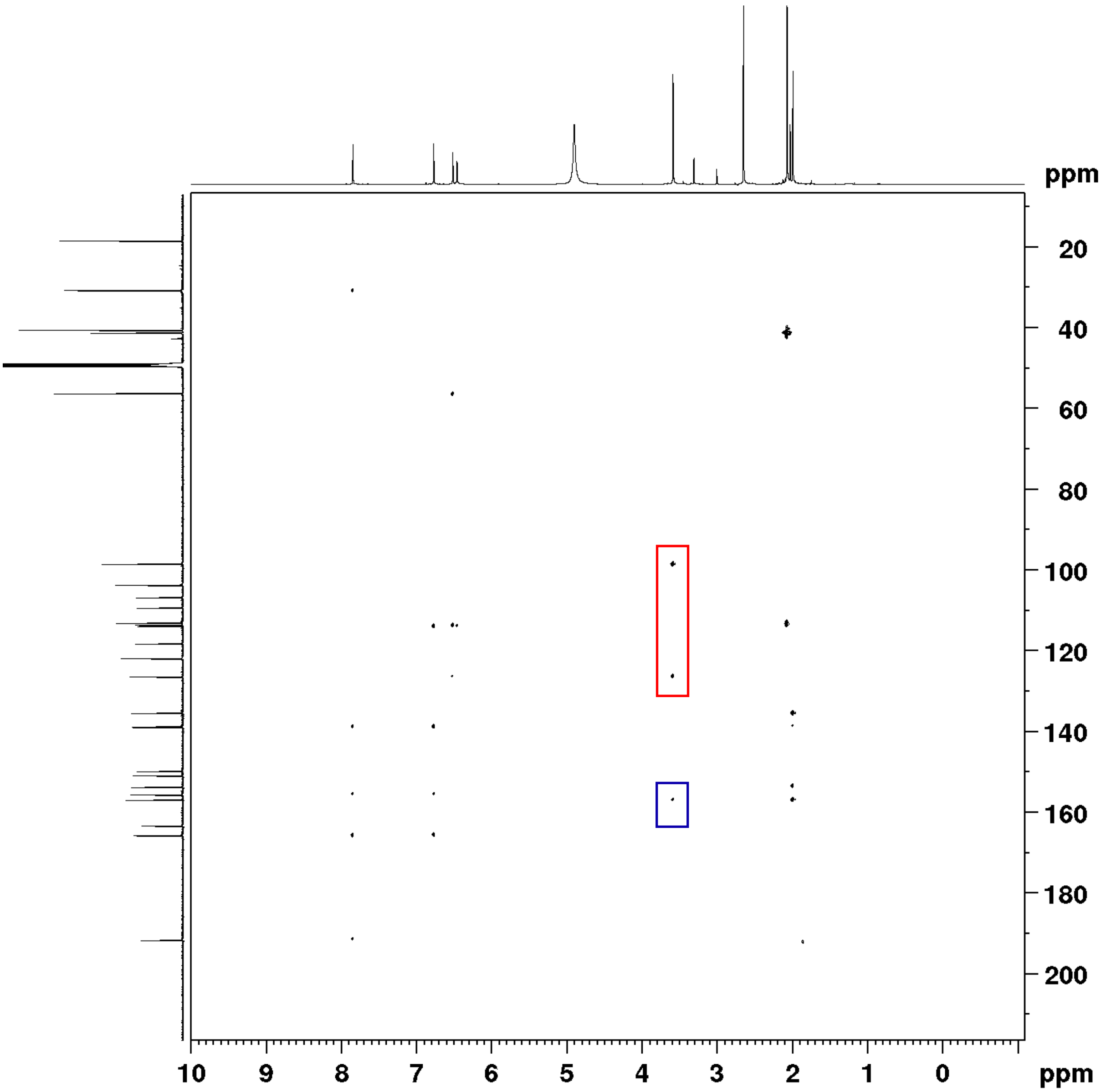

Figure S23. 1,*n*-ADEQUATE NMR in methanol-d_4 for fasamycin N (**20**) with a delay for the $^{n}J_{CC}$ coupling of 7 Hz. Pseudo $^{4}J_{CH}$ correlations are visible from the methoxy protons (δ_H 3.46) to C-4 (δ_C 114.6) and C-6 (δ_C 131.8) indicative of the *ortho*-methoxy regioisomer.

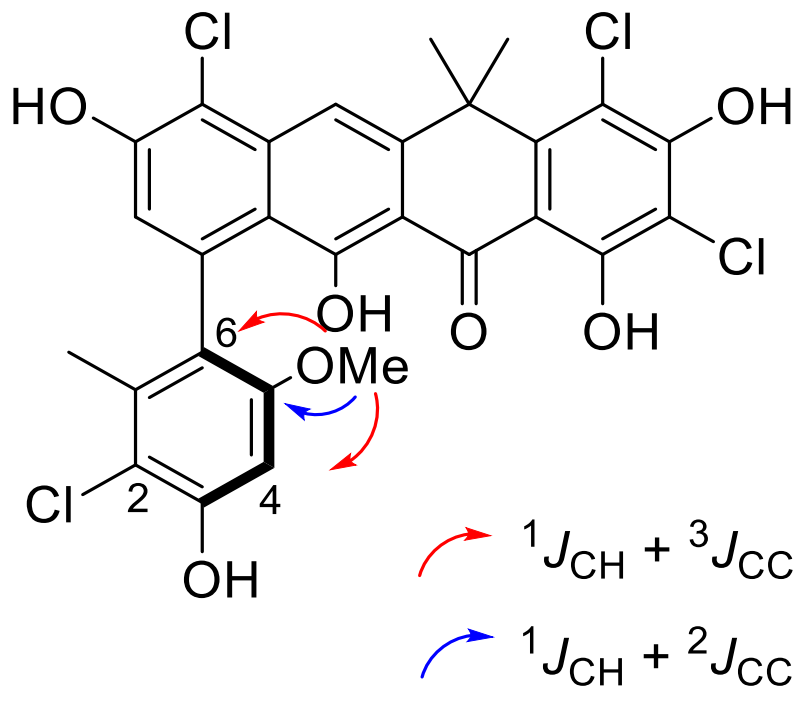

fasamycin Q (21)

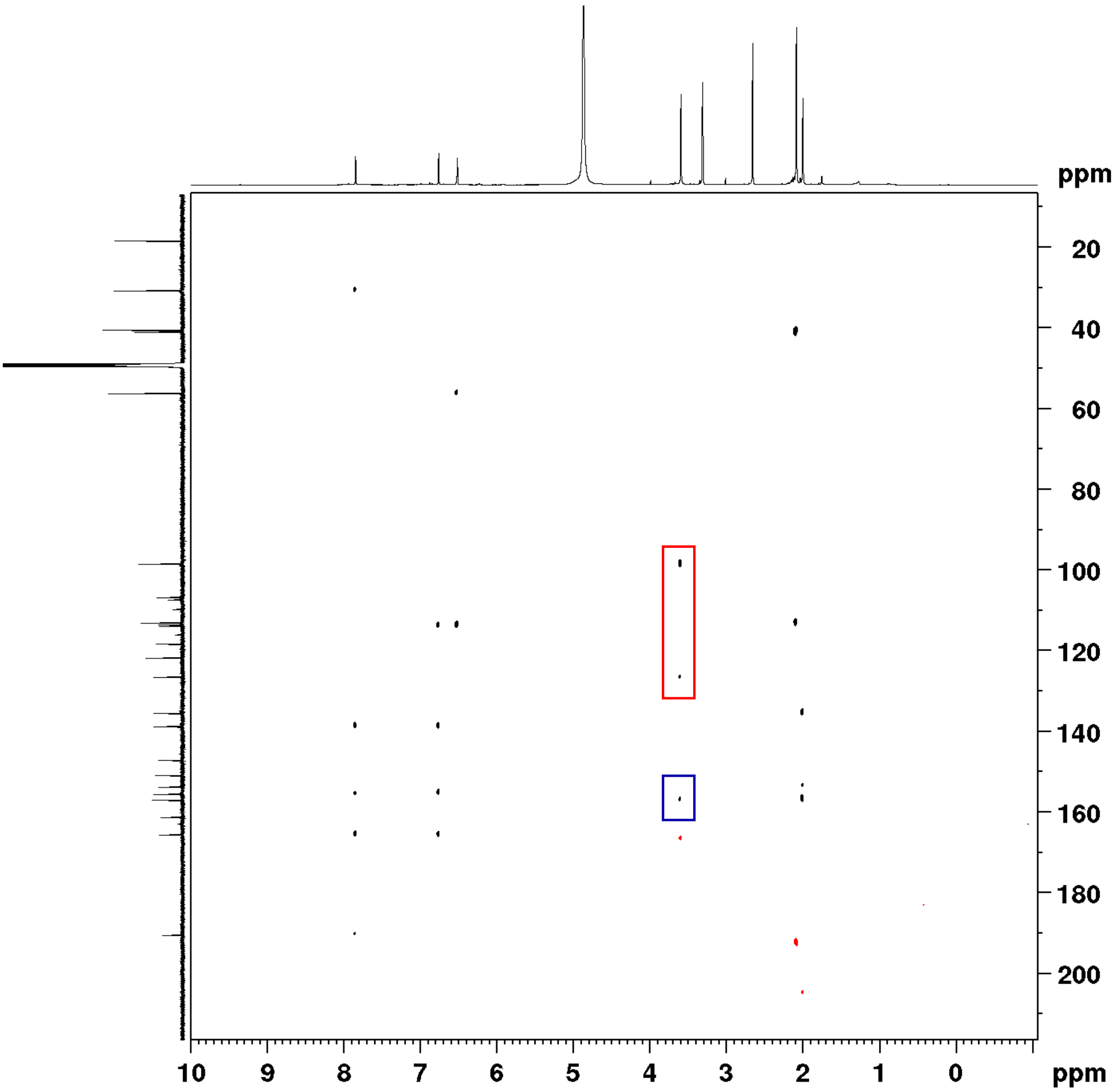

Figure S24. 1,*n*-ADEQUATE NMR in methanol-d_4 for fasamycin Q (**21**) with a delay for the $^nJ_{CC}$ coupling of 7 Hz. Pseudo $^4J_{CH}$ correlations are visible from the methoxy protons (δ_H 3.59) to C-4 (δ_C 98.4) and C-6 (δ_C 126.4) indicative of the *ortho*-methoxy regioisomer.

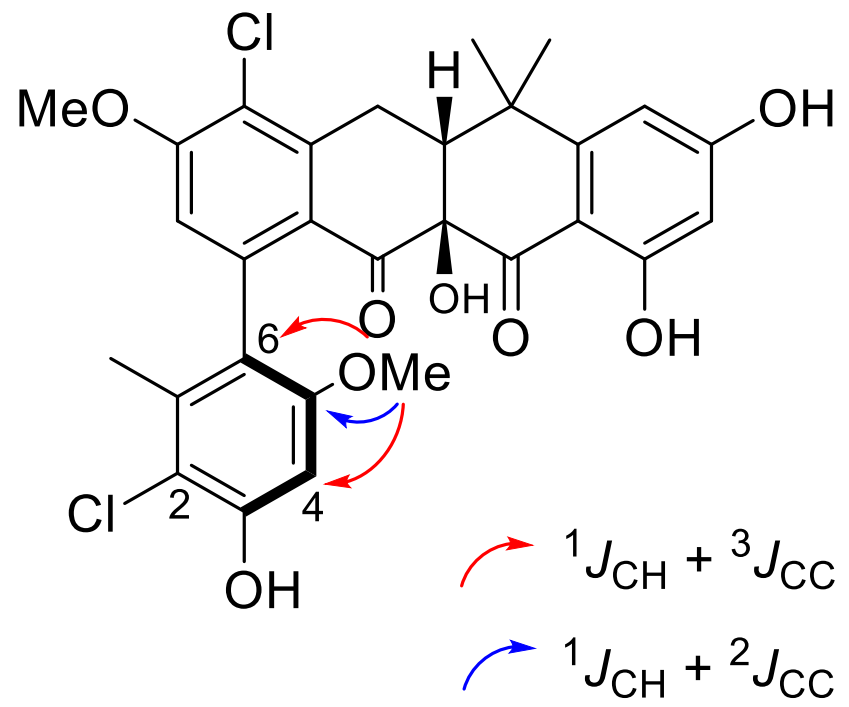

formicamycin B (15)

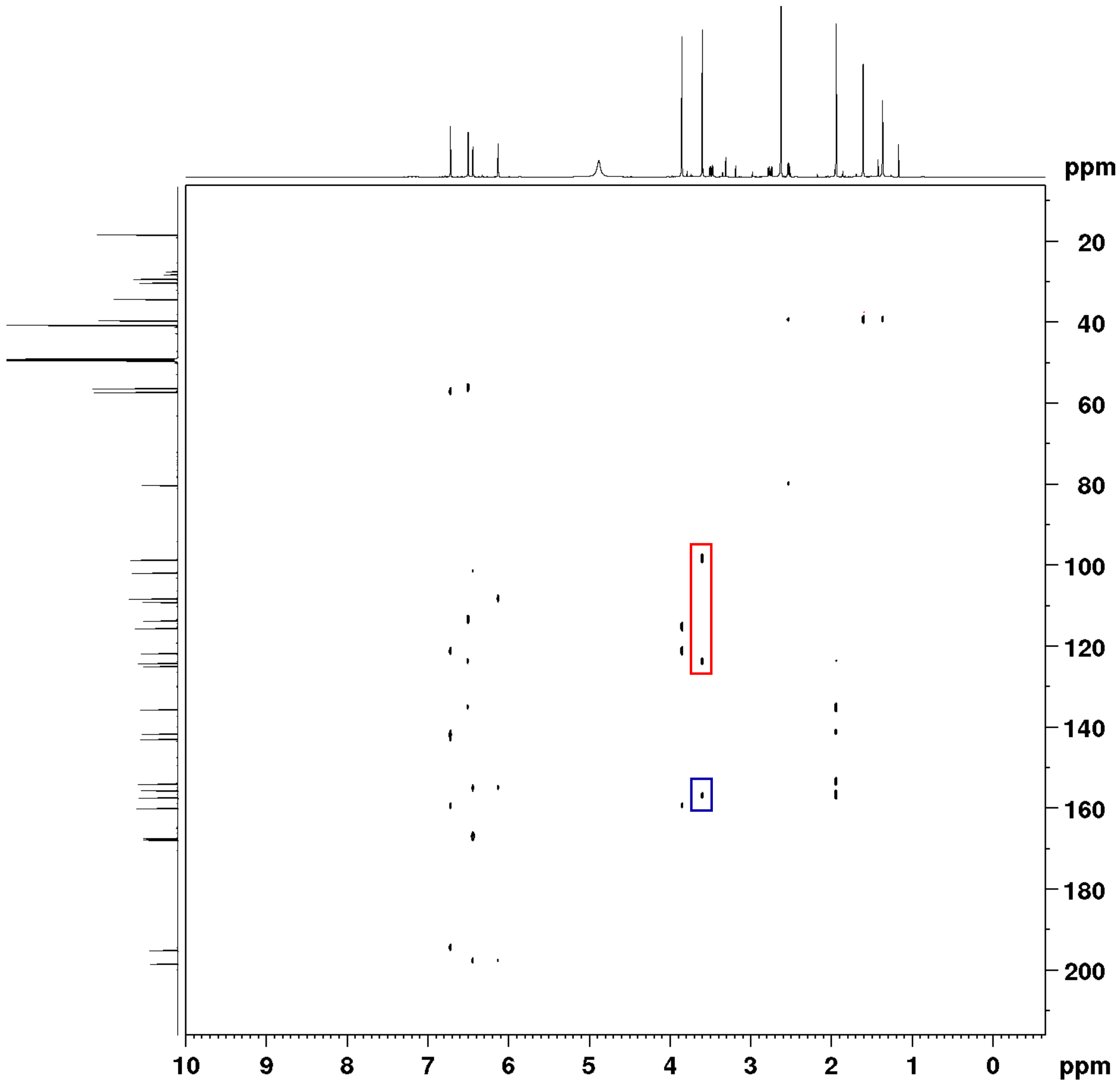

Figure S25. 1,*n*-ADEQUATE NMR in methanol-d_4 for formicamycin B (**15**) with a delay for the $^{n}J_{CC}$ coupling of 7 Hz. Pseudo $^{4}J_{CH}$ correlations are visible from the methoxy protons (δ_H 3.60) to C-4 (δ_C 98.5) and C-6 (δ_C 124.1) indicative of the *ortho*-methoxy regioisomer.

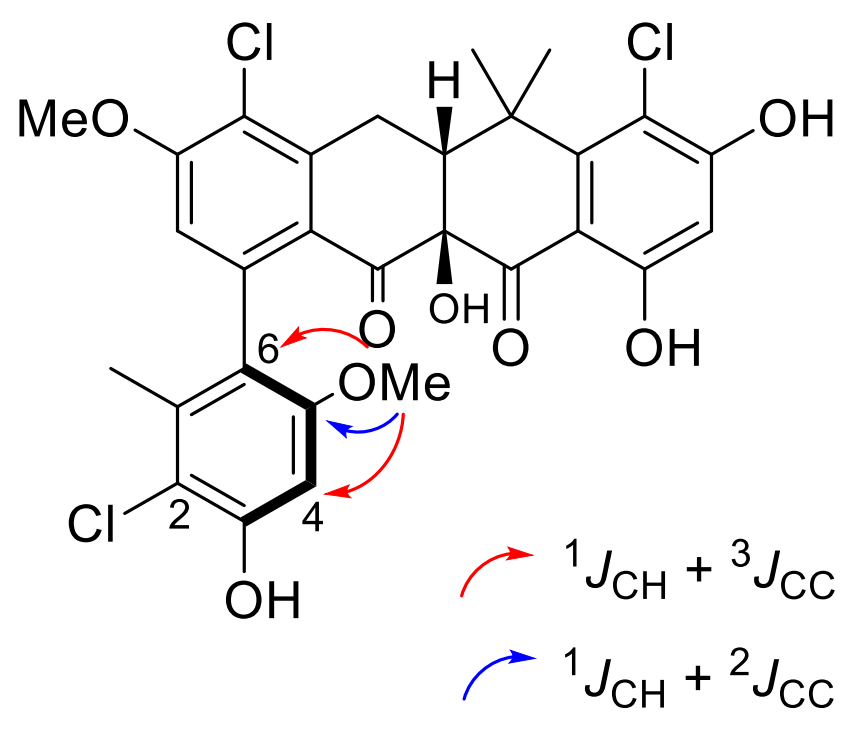

formicamycin D (16)

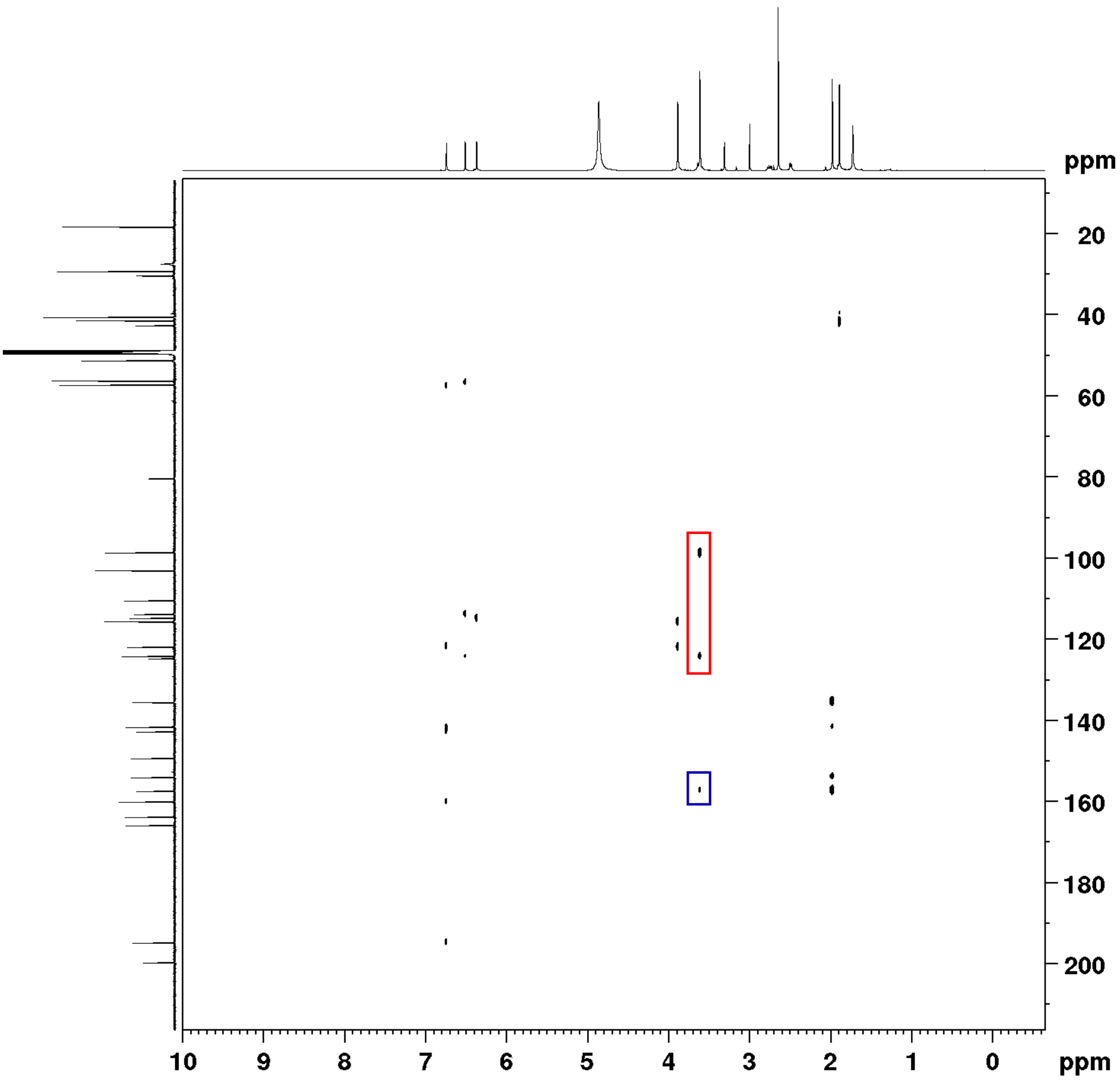

Figure S26. 1,*n*-ADEQUATE NMR in methanol-d_4 for formicamycin D (**16**) with a delay for the $^nJ_{CC}$ coupling of 7 Hz. Pseudo $^4J_{CH}$ correlations are visible from the methoxy protons (δ_H 3.61) to C-4 (δ_C 98.5) and C-6 (δ_C 124.1) indicative of the *ortho*-methoxy regioisomer.

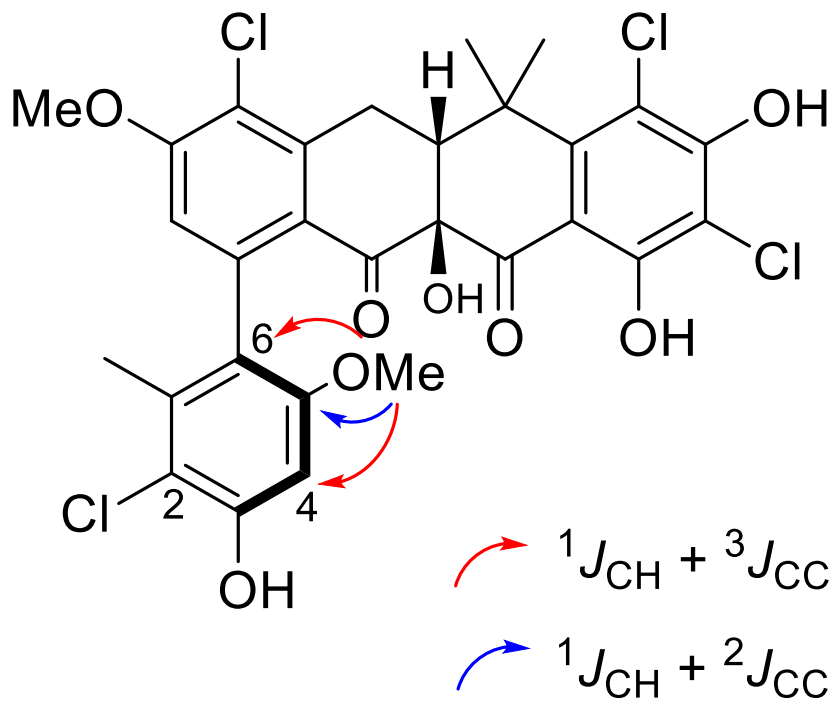

formicamycin I (3)

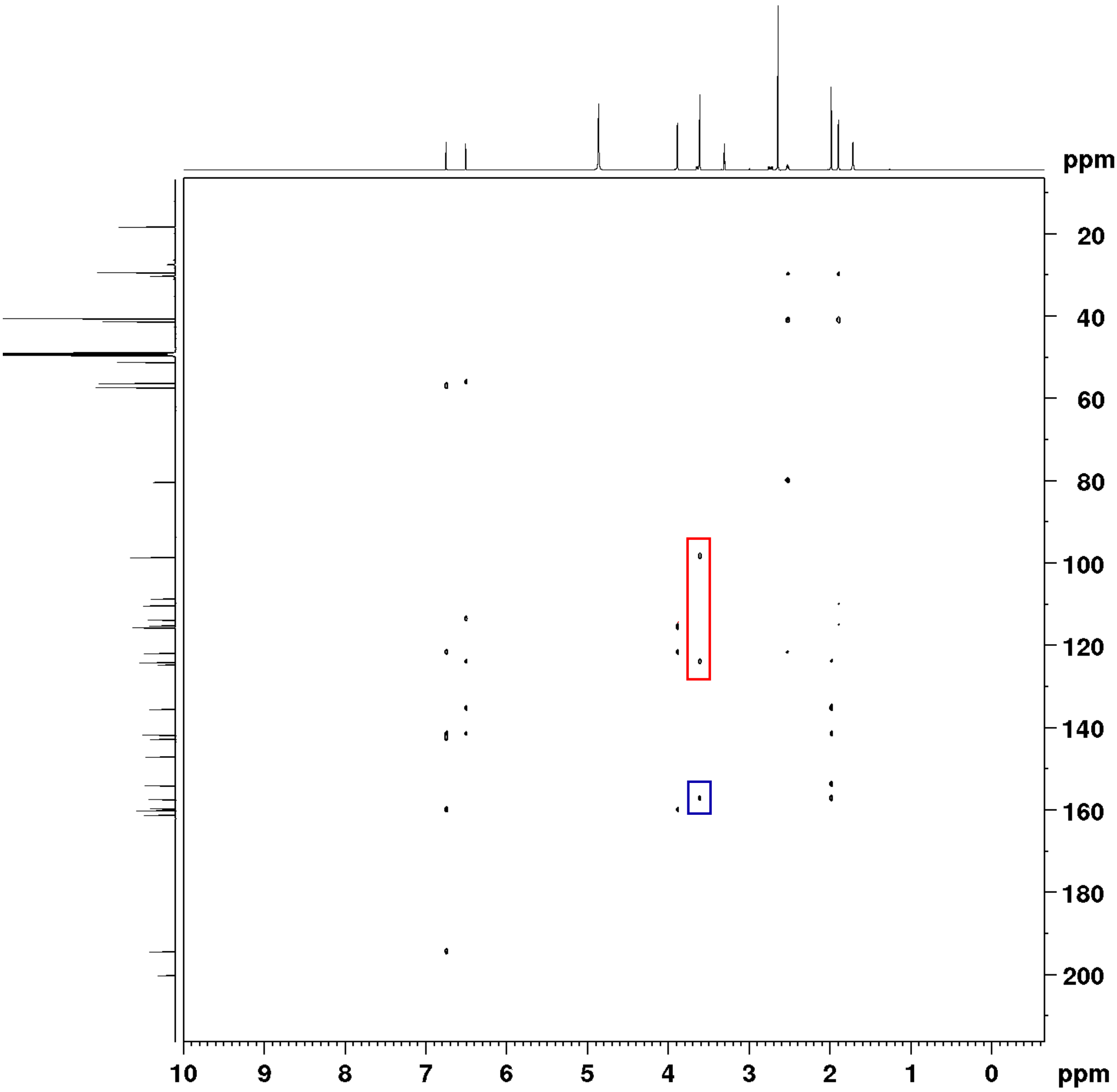

Figure S27. 1,*n*-ADEQUATE NMR in methanol-d_4 for formicamycin I (**3**) with a delay for the $^{n}J_{CC}$ coupling of 7 Hz. Pseudo $^{4}J_{CH}$ correlations are visible from the methoxy protons (δ_H 3.62) to C-4 (δ_C 98.5) and C-6 (δ_C 124.1) indicative of the *ortho*-methoxy regioisomer.

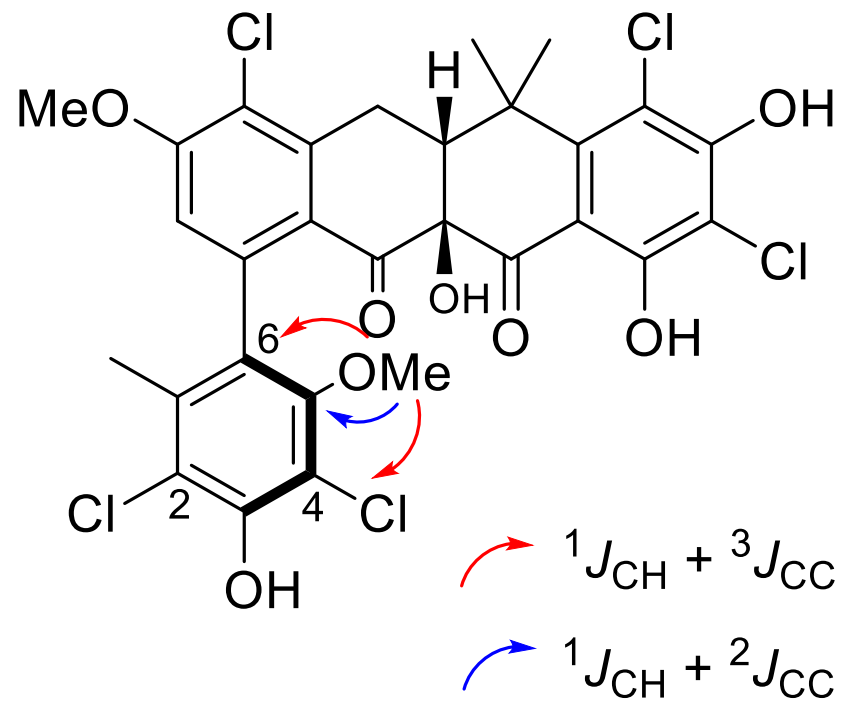

formicamycin R (5)

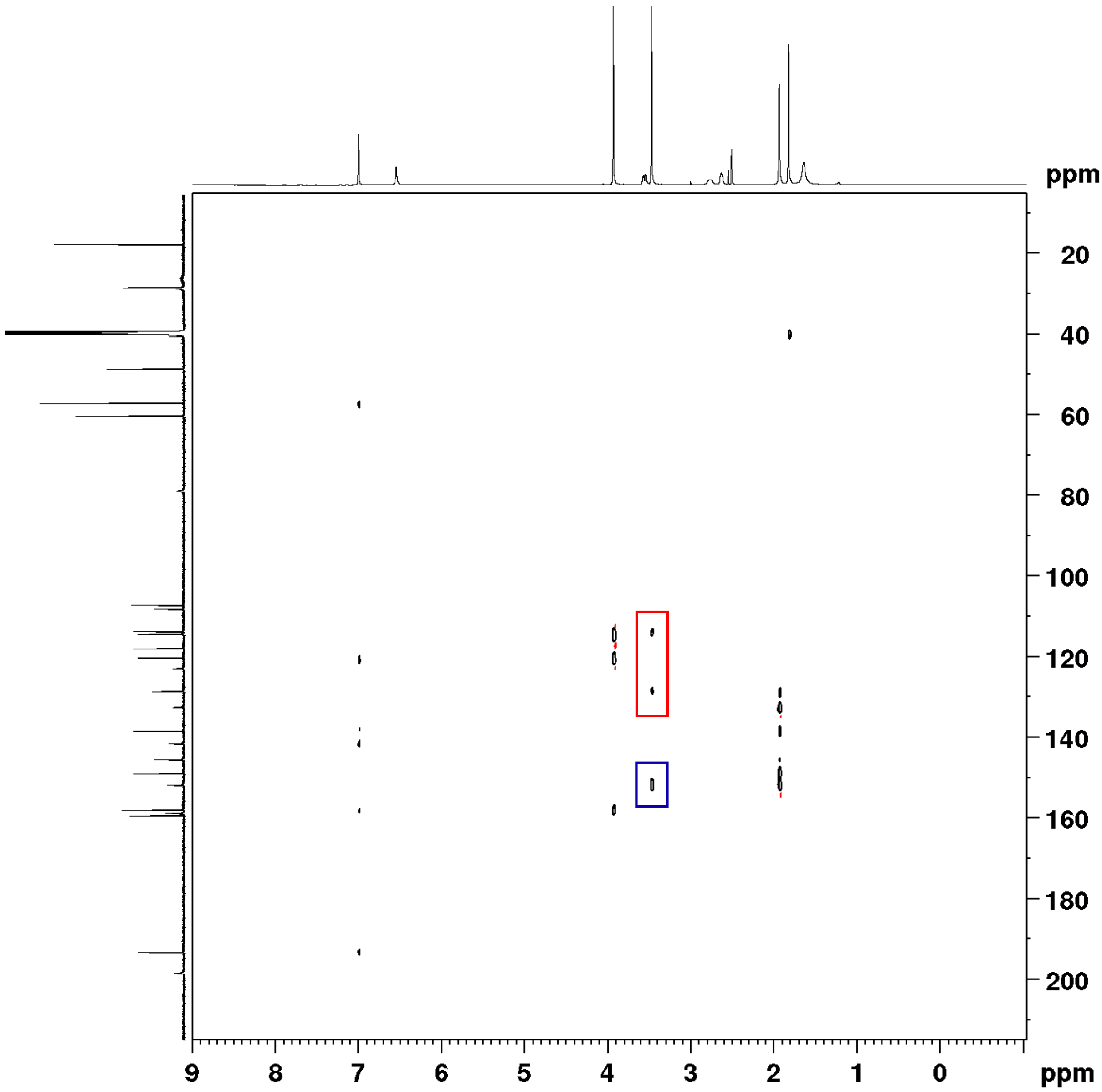

Figure S28. 1,*n*-ADEQUATE NMR in DMSO-d_6 for formicamycin R (**5**) with a delay for the $^nJ_{CC}$ coupling of 7 Hz. Pseudo $^4J_{CH}$ correlations are visible from the methoxy protons (δ_H 3.48) to C-4 (δ_C 113.6) and C-6 (δ_C 151.7) indicative of the *ortho*-methoxy regioisomer.

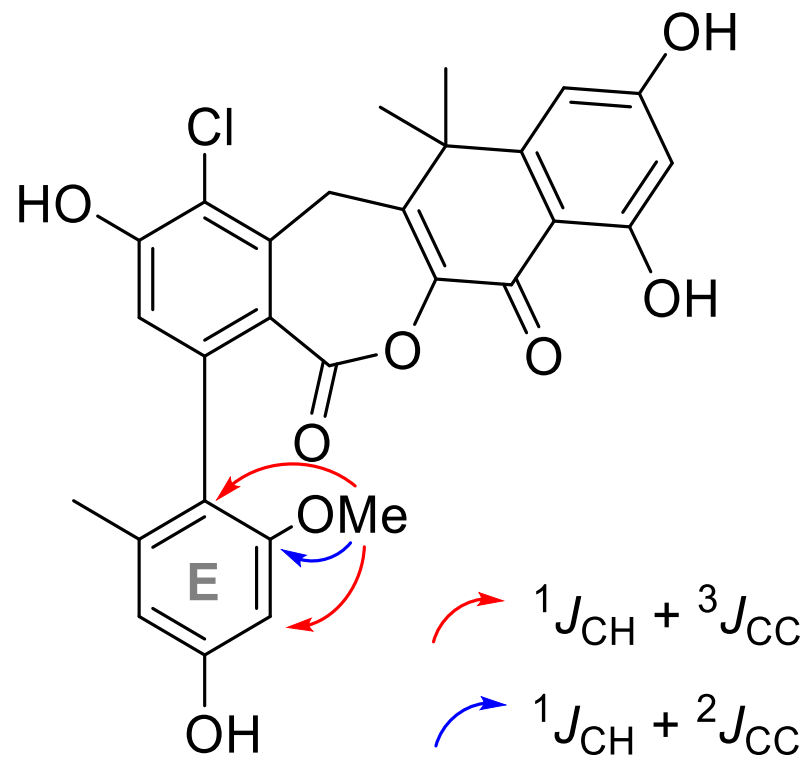

Lactone intermediate A (11)

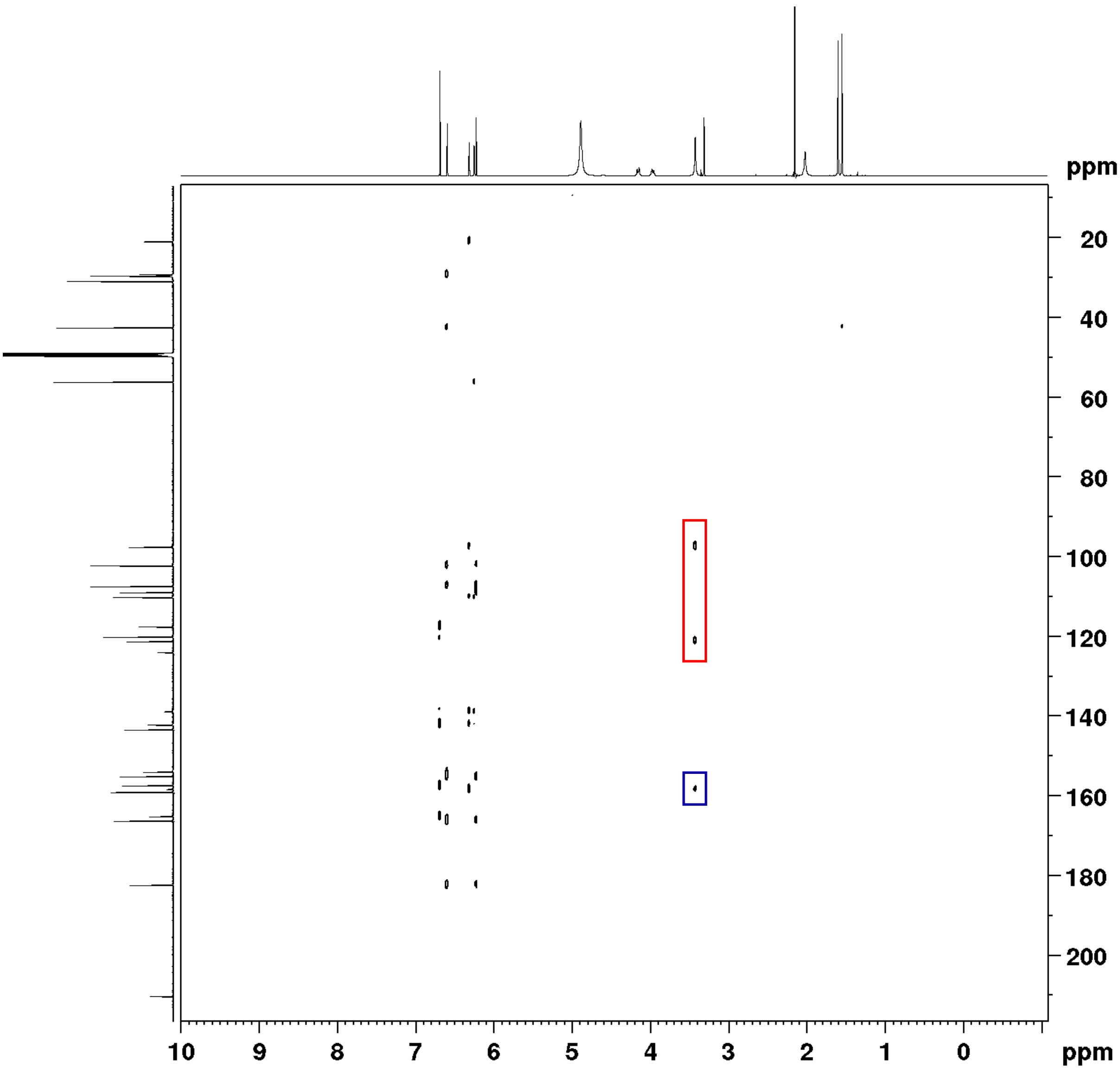

Figure S29. 1,*n*-ADEQUATE NMR in methanol-d_4 for lactone intermediate A (**11**) with a delay for the $^nJ_{CC}$ coupling of 7 Hz. Pseudo $^4J_{CH}$ correlations are visible from the methoxy protons (δ_H 3.42) to C-4 (δ_C 97.4) and C-6 (δ_C 121.1) indicative of the *ortho*-methoxy regioisomer.

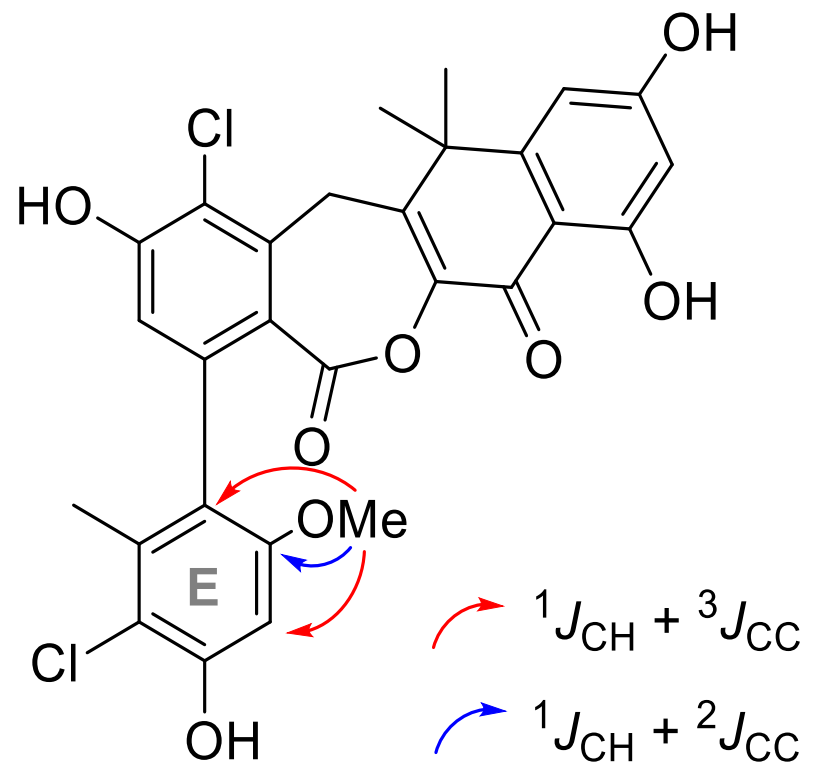

Lactone intermediate B (12)

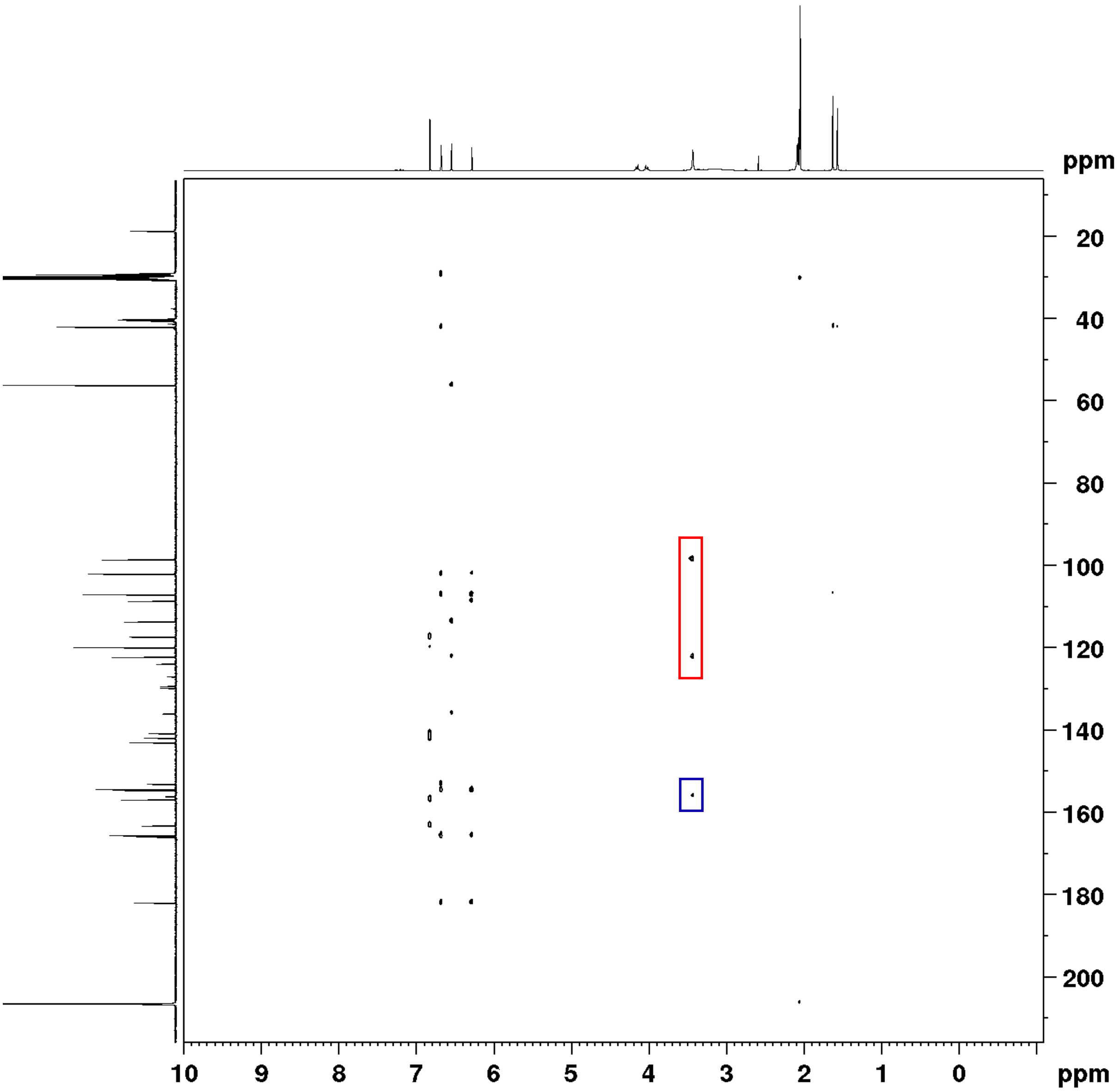

Figure S30. 1,*n*-ADEQUATE NMR in acetone-d_6 for lactone intermediate B (**12**) with a delay for the $^nJ_{CC}$ coupling of 7 Hz. Pseudo $^4J_{CH}$ correlations are visible from the methoxy protons (δ_H 3.43) to C-4 (δ_C 98.4) and C-6 (δ_C 122.1) indicative of the *ortho*-methoxy regioisomer.

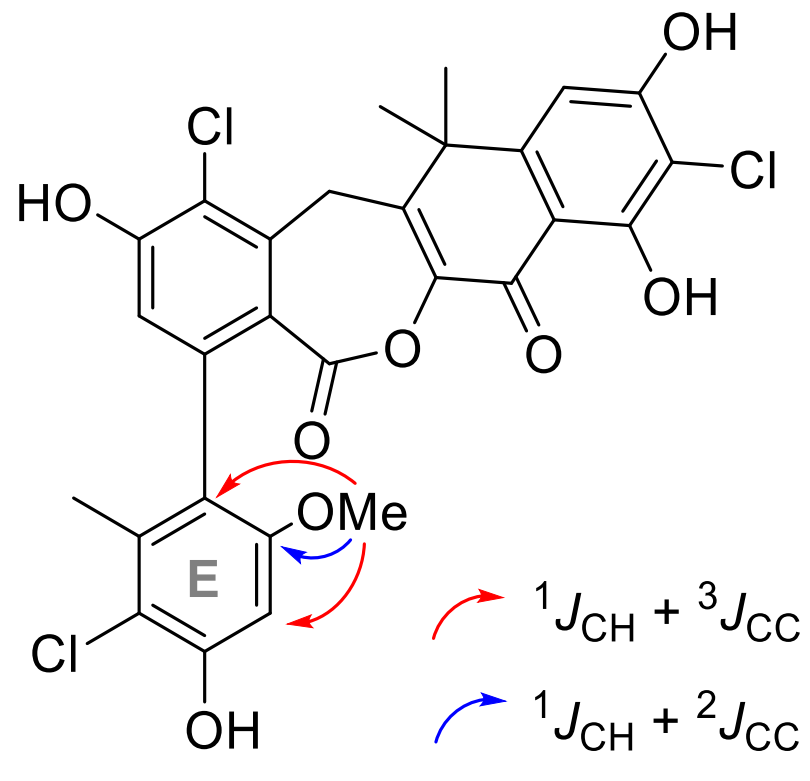

Lactone intermediate D (22)

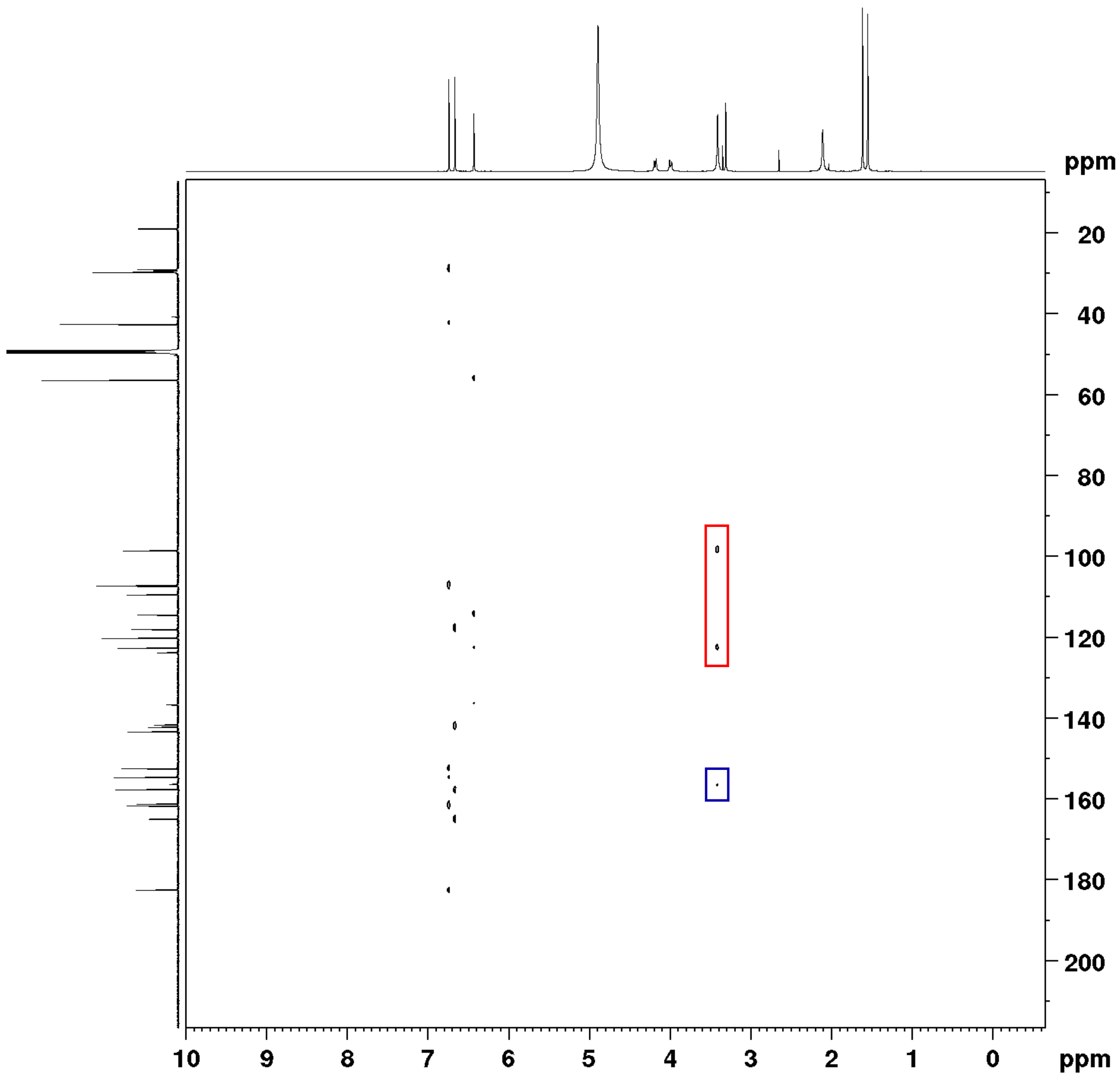

Figure S31. 1,*n*-ADEQUATE NMR in methanol-d_4 for lactone intermediate D (**22**) with a delay for the $^nJ_{CC}$ coupling of 7 Hz. Pseudo $^4J_{CH}$ correlations are visible from the methoxy protons (δ_H 3.41) to C-4 (δ_C 98.4) and C-6 (δ_C 122.4) indicative of the *ortho*-methoxy regioisomer.

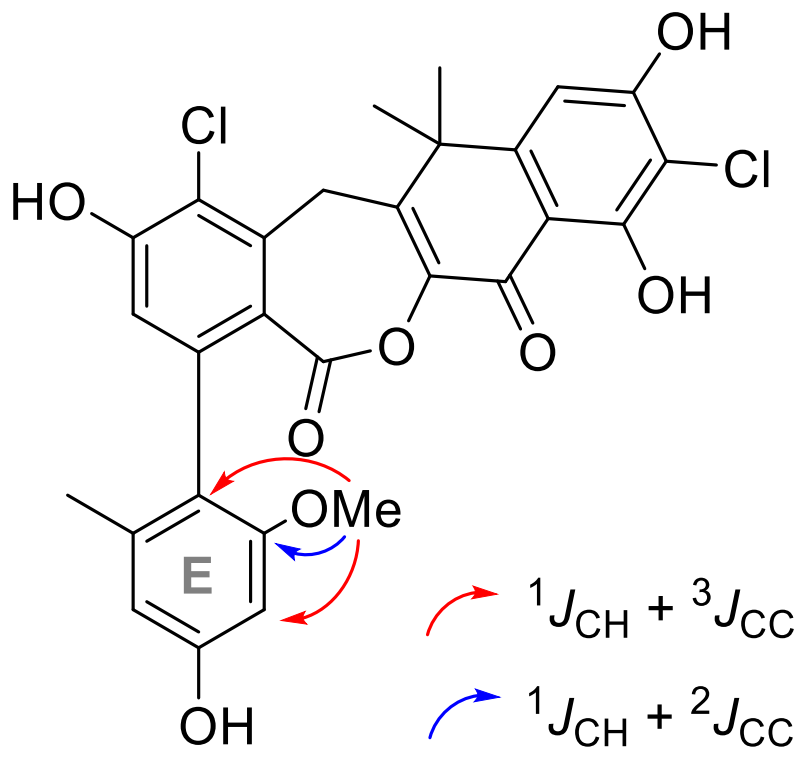

Lactone intermediate F (25)

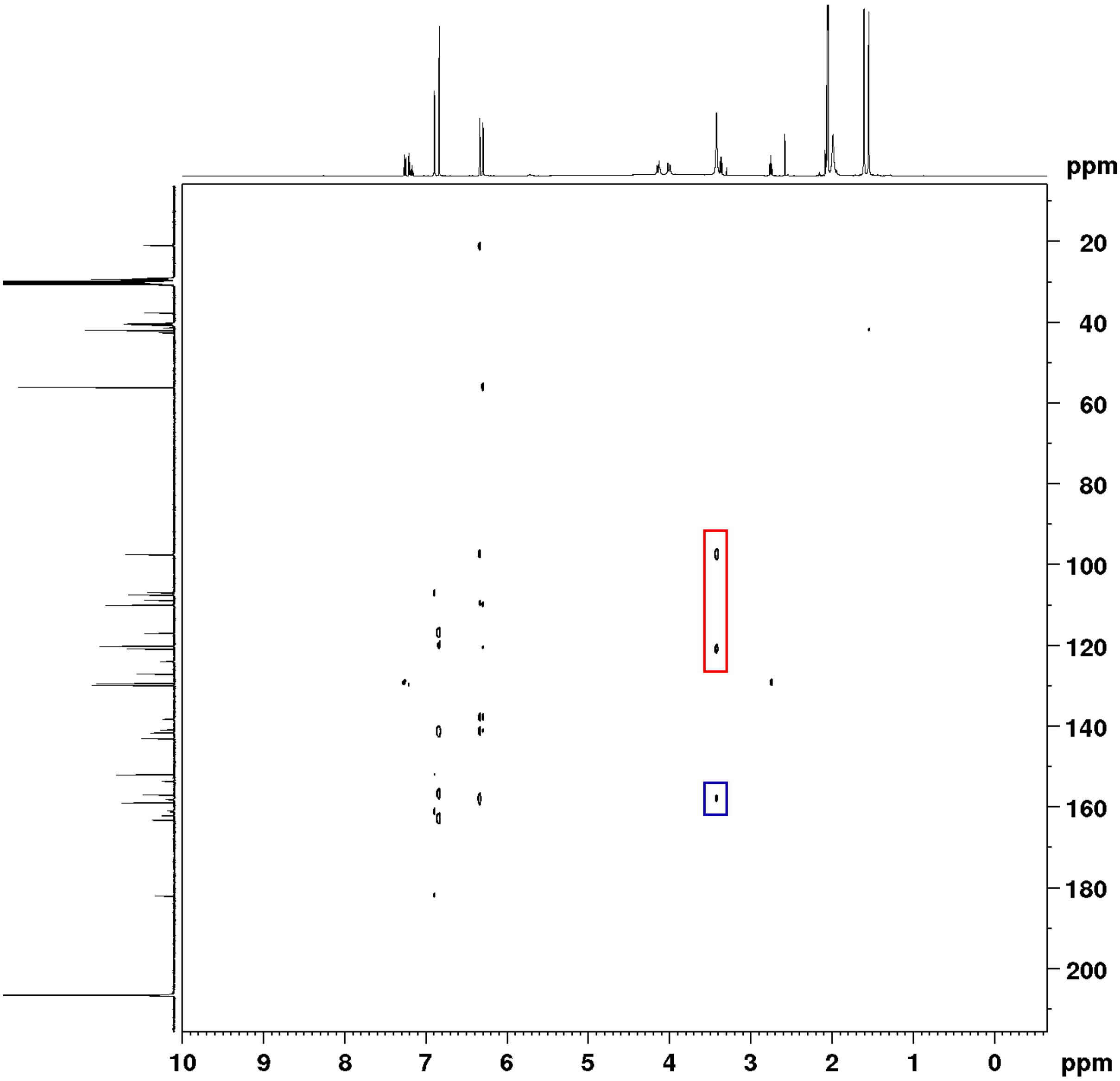

Figure S32. 1,*n*-ADEQUATE NMR in acetone-d_6 for lactone intermediate F (**25**) with a delay for the $^nJ_{CC}$ coupling of 7 Hz. Pseudo $^4J_{CH}$ correlations are visible from the methoxy protons (δ_H 3.42) to C-4 (δ_C 97.4) and C-6 (δ_C 121.1) indicative of the *ortho*-methoxy regioisomer.

11. DFT Coordinates

Formicamycin I (3)

Lowest energy state

Cl	-3.00452316455973	12.79415027849682	8.80604062838196
Cl	4.74825049382162	5.63638255387558	8.04195173742411
Cl	7.74087543857670	7.70497797923787	11.96542633342731
Cl	6.38693718158605	14.26028809615149	8.47331981397296
O	-3.48821584585661	14.71221488029451	11.03543543586270
H	-4.02748599697286	14.13058935877765	10.47074493718704
O	1.09729895920346	15.85912059885054	11.74903498281415
O	1.35286538780688	12.32193831874402	11.82280897680037
O	2.76118817556356	10.35939612054727	12.60054524192791
H	1.86557868176310	10.75792132917125	12.69564173638231
O	2.24332751164345	10.06461973296189	9.56351647072917
O	3.01594075900248	7.94154273043953	8.45934743832115
H	2.46568281700851	8.75206918090034	8.66730146238420
O	6.98946275449818	5.89396592408239	9.99123879323187
H	6.70184401677921	5.25627991082391	9.31098844779190
O	4.39687370317624	16.12054109282498	7.61794505034792
C	-0.39518160432891	13.58395886670529	9.32417052691122
C	-1.75220717797922	13.71370643156374	9.66169000564136
C	-2.18459997816659	14.56103563055158	10.68933465576282
C	-1.24211066334763	15.30283928199068	11.40701329296516
H	-1.60376251925930	15.94603426270694	12.19910183533560
C	0.10925537040346	15.18278676116295	11.09499478352405
C	0.54168877911636	14.33172893669824	10.05672270466478
C	2.00549290198674	14.26929465834890	9.73967844849380
C	2.88208738796648	13.28587377061275	10.25969338048043
C	2.41029483504041	12.26354134634561	11.20599628978274
C	3.30692799957876	11.03116760255870	11.48261638346625

C	3.23357143586937	10.03943908048242	10.31869853824319
C	4.28963490498977	9.03464730096784	10.21367578532688
C	4.07017292642156	7.98259315631908	9.26961159305741
C	4.99325611731476	6.93720680411298	9.18904879886350
C	6.10320469756447	6.90180578367877	10.02996781372085
C	6.32197853025429	7.95563665290270	10.95447816664072
C	5.46084213350927	9.05404971656063	11.04872283599588
C	5.71446750944982	10.24586162228173	12.01285763558757
C	4.76317643388213	11.45023839324796	11.67542691128155
H	4.80571136769375	12.13006995646156	12.53700351932101
C	5.21550037658820	12.23028549726109	10.41633506617242
H	6.16226803041099	12.73032998146026	10.62549910483290
H	5.43903564860272	11.52642981582338	9.60195714820244
C	4.24850850045511	13.26963013092836	9.88407401788008
C	4.71045543110159	14.23854561531784	8.99061923264750
C	3.85263002235698	15.22654692305591	8.46842640753208
C	2.50601405745093	15.22098148801825	8.85288393452208
H	1.82550359994276	15.96558951420260	8.45782874409193
C	0.06327162413746	12.63935149118300	8.23735593546831
H	0.38368396376984	11.68355964829956	8.67040751687471
H	0.92080184499556	13.05090257173856	7.69784760781635
H	-0.73071884564250	12.43469632404307	7.51656208168826
C	7.16759161816731	10.79645638382104	11.92566789466794
H	7.87410081510824	10.16808805719290	12.46308890963014
H	7.19549634730852	11.78707493251633	12.39562582151419
H	7.52382561407396	10.89700705226783	10.89608913771409
C	5.47885592465877	9.78644421063610	13.47913589858566
H	4.46934601116712	9.40708464812646	13.62852501403225
H	5.62416649099906	10.64083547020691	14.15182793214220
H	6.20025620991479	9.01487341317078	13.75752742147162
C	3.56972760680411	17.14192617437661	7.06787576362512

H	4.22550074402813	17.73340406731805	6.42751969267217
H	3.14967977787710	17.78100843820381	7.85401121355812
H	2.75723636611251	16.71577735452490	6.46684513028130
C	0.73927004063007	16.67624461998390	12.85318456213227
H	0.08231867609759	17.50196568924911	12.54847809475294
H	1.67608302784108	17.08406470292145	13.23813346727587
H	0.24483821404175	16.09217168170986	13.64003585616033

Transition state 1

Cl	-3.030503000	12.644904000	11.819081000
Cl	4.240762000	6.032737000	7.076656000
Cl	7.920141000	7.392888000	10.716023000
Cl	6.459516000	14.896570000	9.701985000
O	-3.623353000	15.266335000	10.558858000
H	-4.117115000	14.604819000	11.076821000
O	0.685643000	15.990171000	8.749227000
O	2.072001000	12.166414000	12.745720000
O	3.183234000	9.891109000	12.640161000
H	2.548425000	10.433176000	13.163193000
O	2.180122000	10.143776000	9.720317000
O	2.685398000	8.244272000	8.156338000
H	2.205755000	9.011464000	8.584415000
O	6.782466000	5.940611000	8.632277000
H	6.363330000	5.429320000	7.914645000
O	4.502713000	16.940012000	10.110964000
C	-0.485050000	13.303349000	10.928840000
C	-1.836217000	13.686284000	11.019803000
C	-2.336176000	14.864675000	10.465512000
C	-1.463670000	15.633961000	9.701588000
H	-1.874824000	16.480805000	9.168825000
C	-0.114802000	15.306130000	9.626230000

C	0.470741000	14.232756000	10.401321000
C	1.976462000	14.238150000	10.559354000
C	2.918966000	13.165884000	10.775657000
C	2.729755000	12.048324000	11.714023000
C	3.563250000	10.747454000	11.582795000
C	3.273659000	9.980548000	10.292963000
C	4.265915000	9.011316000	9.834420000
C	3.858297000	8.139504000	8.775097000
C	4.717997000	7.117138000	8.366865000
C	5.950694000	6.932222000	8.988906000
C	6.357406000	7.813124000	10.023931000
C	5.560445000	8.882147000	10.446655000
C	6.013657000	9.895463000	11.531657000
C	5.047414000	11.130835000	11.592370000
H	5.254338000	11.637961000	12.544293000
C	5.258058000	12.153603000	10.451662000
H	6.274242000	12.536899000	10.496014000
H	5.174048000	11.648065000	9.478526000
C	4.299984000	13.335158000	10.450518000
C	4.785067000	14.606156000	10.140312000
C	3.935834000	15.718473000	10.243754000
C	2.571722000	15.507943000	10.429800000
H	1.937943000	16.377512000	10.427567000
C	-0.197350000	11.857923000	11.250918000
H	-0.028043000	11.676118000	12.314841000
H	0.657834000	11.485712000	10.692286000
H	-1.059057000	11.258458000	10.944753000
C	7.438408000	10.460741000	11.265051000
H	8.215737000	9.752969000	11.544437000
H	7.581598000	11.357535000	11.879595000
H	7.595705000	10.736149000	10.217679000

C	6.036847000	9.196229000	12.920328000
H	5.056679000	8.805443000	13.189614000
H	6.334426000	9.924972000	13.684603000
H	6.767150000	8.384141000	12.930494000
C	3.685709000	18.098064000	10.233153000
H	4.361359000	18.945747000	10.108068000
H	3.211087000	18.151321000	11.220975000
H	2.913483000	18.126134000	9.454592000
C	0.084749000	16.799372000	7.746923000
H	-0.680122000	16.244631000	7.189676000
H	0.897947000	17.079043000	7.073825000
H	-0.362785000	17.709670000	8.167409000

Transition state 2

Cl	-2.71893217957772	15.87787767553824	13.31813972405182
Cl	8.73975078864626	9.12601629582002	5.92465169721900
Cl	8.36373244221453	8.30131308812490	11.20005298429240
Cl	5.66476574083390	15.52620995668509	10.66874876627814
O	-4.13647968074227	13.59558919752809	12.02889272065229
H	-4.38854879087006	14.36938954717673	12.56406805874504
O	-0.25052774259709	11.49230994510716	10.33451659136861
O	1.61494755661675	11.51358875507020	8.71038166706629
O	3.08154136903710	9.50595042977507	9.63306847127480
H	2.36224281096323	9.59780090387044	8.97473993866636
O	4.18261310743318	11.41089369545778	7.40502090602358
O	6.20582150648354	10.53651610916708	6.19462040132975
H	5.34156168340894	11.01217351023230	6.36768885043149
O	9.46889096252151	8.15163446859792	8.64670751457851
H	9.83688715031798	8.09972297247920	7.74463284508198
O	3.30076897615346	17.10532911761995	10.11914619058016
C	-0.58952549212143	14.68624626379921	12.00658488382468
C	-1.96226511215115	14.64882916975100	12.28585049297910

C	-2.80351468888111	13.63762195940335	11.80537301383310
C	-2.22133579010015	12.57961151417288	11.11669719815642
H	-2.85592662498990	11.74974999805707	10.83536889882357
C	-0.85306646065552	12.57997835476019	10.83917939445763
C	-0.02404891803115	13.71138706343426	11.11642972771604
C	1.35411364065427	13.96098741439364	10.56957784630778
C	2.45403525793405	13.04762875870853	10.35145224522304
C	2.44366227475065	11.79237747367085	9.56608677524554
C	3.64214240878467	10.81629815848672	9.68333810103880
C	4.58000976867011	10.90140375719930	8.46385752912094
C	5.89285791576097	10.25608459724954	8.58688681357784
C	6.62341828195543	10.07576958835427	7.37134287451018
C	7.83056850686357	9.37255495162097	7.40225667965546
C	8.32127290676960	8.84868226585294	8.59620027658652
C	7.60593605366585	9.05840642996212	9.80180508220088
C	6.40771287311958	9.78105237206297	9.83908732126393
C	5.64283537814403	10.07725317242691	11.15652550972566
C	4.47612648163479	11.09391602669143	10.91926651732359
H	3.82326765286622	11.03388442521178	11.80015050311664
C	4.92775575566018	12.56417706906329	10.75776259918669
H	5.42945916084998	12.90390569369958	11.66814752594130
H	5.68066549660158	12.65848690277685	9.96484939624942
C	3.78142794075474	13.53189847332731	10.52144186358866
C	4.03518084691108	14.90457835115066	10.51048867246840
C	2.98070689125556	15.80680075207263	10.30583819599532
C	1.67036908749801	15.31960482033619	10.39701065106421
H	0.85857012086153	16.03343007910350	10.39085645921151
C	0.23044409495665	15.68305200230047	12.81111049929458
H	1.29497003696984	15.45811825776907	12.77719888223242
H	-0.09327644448841	15.64162568842440	13.85639688354983
H	0.08918092596380	16.72067903599329	12.48475087937214

C	6.56272938703520	10.69742670738091	12.24500532673208
H	7.20428306903316	9.95264631673251	12.71129347738508
H	5.93499088118305	11.12598080993116	13.03616128769845
H	7.20372887088802	11.49218465876110	11.85041500215084
C	5.04143697949303	8.76219052276773	11.72593766703731
H	4.34567503909627	8.30402134044549	11.02331659764337
H	4.49523160054049	8.98639349075977	12.65081725552752
H	5.82845932571205	8.04552350617286	11.96927632021301
C	2.26210517412573	18.04418589382410	9.86042406883259
H	2.76766209581399	18.98985354741058	9.65943556094291
H	1.60351146357184	18.16300526775420	10.73007028982428
H	1.66793268195045	17.75146861695551	8.98636831651079
C	-1.01941777409777	10.40090058517032	9.85171330003629
H	-1.57161087824189	9.91064006794598	10.66372997564751
H	-0.29603485805299	9.70484645918761	9.42712533875783
H	-1.71839198733287	10.72111469925816	9.06982569457425

Fasamycin E (7)

Lowest energy state

Cl	3.12523762240096	-1.95891337654851	2.68809765276751
Cl	6.48865805900765	6.27282948029663	6.14462952574548
O	2.20095001427457	3.83680641574038	2.08874305649962
O	1.38669398030201	-0.73453956698731	0.71362572641849
H	0.79668244416454	-0.28338527765836	0.08992713067588
O	1.39862390364829	2.51730082074695	5.08590972556120
H	0.53380948271651	2.44500619654760	5.59034938979133
O	-0.40856542962772	2.90076010132604	6.76720217886533
O	-2.30165386668741	3.19204747223862	8.43565044716081
H	-1.78680557857610	2.84630439994152	7.65861079647792
O	-1.83236922815681	6.29954410626242	11.90402230332458
H	-1.20630145826074	6.94961666187341	12.26102774344004
O	7.24978233728825	4.75411977171825	3.80197995011227

H	7.45578118473686	4.22201744885118	3.01694375796892
C	3.49552198946489	0.68176588582790	3.36501946734201
C	2.85206326612666	-0.23203489888297	2.51082289499204
C	1.98009038925878	0.19891994662250	1.50847466482099
C	1.74257076932516	1.56882168848878	1.34440905009580
H	1.06467961303991	1.89582743011389	0.56232196935979
C	2.37535847160350	2.48678374772066	2.18081386644594
C	3.25631904963802	2.05500508351331	3.19326015434635
C	3.96388450373752	3.09459554051480	4.01278464582571
C	3.42134698972918	3.74486463478361	5.17025276151102
C	2.12177717514368	3.44866857261854	5.70406070246835
C	1.62296202086005	4.11554017475619	6.85297439251211
C	0.29867829000449	3.78313615186626	7.35747381500490
C	-0.22414054200098	4.45325065188461	8.53599528863603
C	-1.53042963623266	4.11393713086926	9.02680937293426
C	-2.04981139985995	4.74517769448533	10.15685894476717
H	-3.03704425967486	4.47969041856103	10.51643276176799
C	-1.28815351355709	5.70897308463378	10.80664402594581
C	-0.00801293253026	6.05352249405847	10.34151119958465
H	0.55375443450091	6.81185682058702	10.88057603471736
C	0.53296598857483	5.43930725883646	9.21624564520592
C	1.93423248369633	5.85653498679395	8.75065091789143
C	2.42263727140091	5.10437232522992	7.50352148626011
C	3.66793301932870	5.39168923379149	6.99937461367297
H	4.28923550408935	6.13601502808556	7.47831043529479
C	4.19861664820237	4.74353551158241	5.85041007707551
C	5.49344755575333	5.06151169994874	5.35742003991804
C	6.00880681248677	4.42420311218796	4.24161651374880
C	5.22970053779444	3.44670266252568	3.58556905613113
H	5.64146227262011	2.95888949449413	2.70412835370922
C	4.39883701600590	0.16730963367735	4.46113880280417

H	4.80063008964785	0.98163574784044	5.06545034964096
H	5.23815579157888	-0.40448349535574	4.04760089700548
H	3.85073761058961	-0.51441765310462	5.12142628275949
C	1.90751728495416	7.37771762679470	8.42470313744491
H	2.89538964484299	7.72682491765625	8.11087698088090
H	1.60621320081308	7.96259739046264	9.29933000760676
H	1.19977462162042	7.58716805380868	7.61572933740136
C	2.93651410081217	5.58566420473751	9.90932691922410
H	2.96382133045535	4.51896497407774	10.15538641110120
H	2.65360196480757	6.13524609205590	10.81251743818903
H	3.94758639949333	5.89601759354942	9.63097352197599
C	1.28487609520489	4.34125074883230	1.13183912185114
H	1.59096656227259	4.09106237015592	0.10661424594609
H	1.29293233140537	5.42610159275478	1.25360142925463
H	0.26884971574081	3.96388198120851	1.30903258011792

Transition state 1

Cl	0.05615756994789	-0.90003941353322	2.20533726705005
Cl	5.18517870418039	7.26198068683423	4.81952359943721
O	4.69062614945640	2.71194016659352	2.06275020106047
O	2.24617942571651	-1.04209916086549	0.35887319470578
H	3.02497465286989	-1.07939863965286	-0.21878015642519
O	0.01987363570635	3.87261736976309	4.91946066382165
H	-0.67983842256253	3.77717987026056	5.63254918889213
O	-1.09017119500416	3.81402273187013	7.18150936355239
O	-2.22987253416442	3.59935876226596	9.44939992919243
H	-2.09095771008977	3.55662962589973	8.46561689860981
O	0.05335302514651	4.97928735086665	13.29837409278719
H	0.89858528460290	5.36300784410605	13.58161611523636
O	4.51629174620381	6.60558432980328	2.05019374232917
H	4.30134717086333	6.30019034326162	1.15464104491669
C	1.42216594729338	1.36644197344681	3.01959389513099

C	1.37348229249222	0.26604503852853	2.13979466050453
C	2.36672271383487	0.02351879012595	1.19135329049602
C	3.48193928554179	0.85532676751332	1.18534448753768
H	4.31200108166131	0.61624202957779	0.52962515820521
C	3.53292829933059	1.97907413100907	2.00830965214032
C	2.42480041573934	2.37364139369877	2.84335283112348
C	2.52300112361516	3.78961146395518	3.39567584798285
C	2.32391988761430	4.33195840635587	4.74131133682001
C	1.15107503616396	4.20237847433145	5.54545068973074
C	1.14767764967195	4.53955440625090	6.92485230081173
C	-0.03148070679884	4.27259977901413	7.72698468686274
C	0.01041866540903	4.49616592939181	9.16330072045452
C	-1.11338988652733	4.12314915902520	9.97307923686662
C	-1.07712624571591	4.29107151393054	11.35750625156983
H	-1.93078124394137	4.00020306051366	11.95851306035416
C	0.05954613213884	4.83269465527709	11.94588705405079
C	1.16451747809429	5.21631450763273	11.16738954221899
H	2.02679761301503	5.65151245786091	11.66582264868590
C	1.15504865391100	5.05546118909223	9.78517542273720
C	2.36958484052459	5.53577298788514	8.97724684487143
C	2.27343366467262	5.21451904486768	7.48109413004476
C	3.24922878445480	5.65379434045851	6.61774509730638
H	4.04536274763652	6.29756241739461	6.96849362719330
C	3.24121861152983	5.32715249542449	5.23103080192837
C	4.05968650034757	6.02583676976203	4.29996685914916
C	3.88353375309111	5.80648840194511	2.94803283572706
C	3.14571439193664	4.67549557377565	2.52741870908642
H	3.22923599299565	4.41430304436870	1.47754547409360
C	0.46501621323483	1.30917179697977	4.18602136985620
H	0.90367778377272	1.77138505016607	5.06535316414564
H	0.26416262605580	0.26343788150043	4.42687702831729

H	-0.49240957533407	1.79192571698577	3.98000442301471
C	2.47886633798055	7.07753641655127	9.15822073305737
H	3.35280181950543	7.47417664423361	8.63337992719473
H	2.57308163927695	7.34063619812379	10.21658685885125
H	1.58835016384829	7.57656442136070	8.76204379574623
C	3.65401528552474	4.85305484377300	9.52624095222387
H	3.58753034960243	3.76451437102533	9.42626453298351
H	3.81135037440877	5.08900458047803	10.58346426196872
H	4.53376564414869	5.19184228506041	8.97079888037187
C	5.88763632975831	2.16976187085383	1.52801728110702
H	6.08436808624244	1.16221376203686	1.91671009501650
H	6.68223428181714	2.84633516329759	1.84949174863402
H	5.87094065755089	2.13740592768639	0.42957564866002

Transition state 2

Cl	0.67950667599838	1.59156303243777	-0.92270687506983
Cl	3.29922867373626	8.16466007538879	3.82470912264025
O	2.34465139642074	0.99448935939619	4.65645261197382
O	1.09673519896326	-0.91787862814363	0.44271520744326
H	1.20097381205183	-1.73542083478577	0.95501094689579
O	3.88359877057963	2.41279019842576	5.94306177537475
H	3.87711627011303	2.07116431203844	6.88514555410762
O	3.33499505821213	2.15905565868612	8.38732146008957
O	2.73230307824941	1.80782586308685	10.83532994927099
H	3.10226541437223	1.63609284730445	9.92811925170865
O	0.55418558428014	5.23218772694908	13.17455508183376
H	0.21226822034983	6.13265236285263	13.05545035704875
O	4.34973803560995	6.57940260604005	1.60852354132197
H	4.63403738862341	6.00427982194921	0.88037093616399
C	1.68856537961977	2.56089643589116	1.45585922385187
C	1.32519626916537	1.43106266506319	0.70604433856824
C	1.42493806576679	0.13849683586272	1.22914276718080

C	1.78952513788630	-0.00572782438249	2.56431805251312
H	1.76416538073153	-0.99244938373855	3.01384378608703
C	2.15248498012929	1.10570895267039	3.33115462293998
C	2.28924437010127	2.40311991926736	2.75435600058158
C	2.98679738607406	3.60391683492503	3.34762577129802
C	2.92375871796591	4.17897514206435	4.69120369478729
C	3.13677797532821	3.51217157473048	5.94243557463539
C	2.70435464982972	4.08901118746423	7.16864667561252
C	2.79409594254859	3.31835032690443	8.39155333889449
C	2.23433283225320	3.85374561241087	9.62285302353682
C	2.21601191536087	3.04471522495635	10.80714140723458
C	1.64542493241927	3.52450448368072	11.98604528421088
H	1.63350208346289	2.90103883434259	12.87246070698188
C	1.10038950081944	4.80306457752665	12.00474344129892
C	1.12243971105944	5.61540631821800	10.85885800781090
H	0.70152834959734	6.61554832718974	10.92110031027307
C	1.68195696687288	5.15838882657144	9.66930772325678
C	1.72504005769206	6.10559976634791	8.46124007681299
C	2.27787365129317	5.45006868334067	7.19039307661005
C	2.45112631789670	6.19541421400241	6.04859066335034
H	2.28429861246165	7.26460813010664	6.05696739459891
C	2.86651745272217	5.61173398614368	4.81561993928950
C	3.28412267563207	6.41741034599381	3.71751639015891
C	3.78479958805214	5.81532242855388	2.57839988671356
C	3.57935585179225	4.42786472795165	2.39701997167692
H	3.76963048084298	4.02878572378033	1.40499045319535
C	1.26092290674084	3.90300865214816	0.88050721939090
H	0.22657307095577	3.81676822029454	0.53158713198840
H	1.85236358318501	4.20954335832841	0.00960585798960
H	1.30370363967414	4.69863784257243	1.62194893260111
C	0.28594527927475	6.60381959385405	8.14752542157884

H	0.29872016369054	7.29538235224770	7.29975536713331
H	-0.15124960970697	7.12831172889452	9.00312628478397
H	-0.36890850542061	5.76423837161249	7.89140153902207
C	2.62380472024879	7.31805337657798	8.84137111575959
H	3.64720403141022	6.99051898033073	9.05199820223522
H	2.23783491727268	7.82315700135387	9.73239731632524
H	2.66022845213893	8.04916021398647	8.02863117014130
C	2.35688248131947	-0.27288483126116	5.28950487260405
H	1.37996677970690	-0.76924749154224	5.21631304207790
H	2.58041146912288	-0.06896834614793	6.33791520366844
H	3.13311780744847	-0.92599530271671	4.86993679793430

Lactone intermediate B (12)

Lowest energy state

Cl	4.35851432601805	4.68382289620284	25.83874585106817
Cl	10.31964022357218	4.77835739025091	18.48470765271359
O	2.31297873905717	6.35552875873525	24.65178542320501
H	1.63092374321080	6.87886285259169	24.20234798199407
O	4.43252879267911	6.79150320375816	20.32785975078620
O	4.61241408459736	3.29955149959925	20.38686934196206
O	4.70011852830564	3.97505283360486	18.29094947200532
O	2.97139051416674	5.56222646680699	17.06232779615649
O	2.21776090000152	7.02975011981379	15.09199985245344
H	2.18282049187075	6.49390410204148	15.93248712049759
O	5.35970663144282	8.22811118606820	11.82030018537910
H	6.29376608860539	8.10728515108926	11.58615609376529
O	10.20206089258400	6.17920274318082	20.99066698073394
H	10.13552168271040	6.61513630864786	21.85551334977845
C	5.46316252757561	5.00675716678064	23.33966093136334
C	4.39237193401054	5.30992615368214	24.19767126339890
C	3.32098770476828	6.10164789555733	23.77467321074338
C	3.31242304838182	6.60694816007457	22.47075845307918

H	2.47500537051919	7.21574565650046	22.14656481741125
C	4.37147855823466	6.32745612563406	21.60894769520079
C	5.46425418382662	5.53971805607742	22.03541477193770
C	6.61334683010651	5.33063816281664	21.10073674257951
C	6.49970358688450	4.65250336166458	19.86686730299725
C	5.21847519088419	3.94145635381440	19.56614500702220
C	5.17216911899724	4.72837754848259	17.23665011767525
C	4.11459547005810	5.50924453165610	16.56577011335623
C	4.47816079208642	6.20298651917532	15.33970294042897
C	3.47647300000507	6.95098652815219	14.64120770447256
C	3.79237259619898	7.62118944212216	13.46005010885844
H	3.02944853489260	8.18224298772564	12.93295776686624
C	5.09496822105628	7.55851686078794	12.97415810081922
C	6.09158637399123	6.83583544959826	13.65229337634123
H	7.09729936960588	6.82285545097684	13.24008623385053
C	5.79775725328691	6.15596190668150	14.83161999275477
C	6.91767859277159	5.39074295960006	15.55300761519018
C	6.44627014554627	4.67281555770248	16.81096247610941
C	7.42046666484366	3.85740949070695	17.65144734005726
H	7.00626777899042	2.84872107172926	17.78785045229818
H	8.37983091896518	3.73947350709953	17.15237533086267
C	7.61340245602630	4.51687066080214	19.00803546329510
C	8.85487518506224	5.01247213999735	19.42738258058577
C	8.98065269006262	5.70025383273785	20.64455140534695
C	7.85214555719405	5.87305762568688	21.44757060691218
H	7.94404870351065	6.43069474941558	22.37718265869990
C	6.55148483129055	4.07622402645184	23.82473484166074
H	7.22739938929558	3.78907253141254	23.01900167965151
H	6.10931518229104	3.16536604649050	24.24117999400428
H	7.14199527799929	4.53439670468687	24.62822297490561
C	3.31479073185303	7.50056078887668	19.80532700579736

H	3.56298213734170	7.72768385476012	18.76876989403176
H	3.14539165245697	8.43577499270947	20.35550457809031
H	2.40890936487555	6.88383375197083	19.82495537477683
C	7.48658297597027	4.31455349673457	14.58073415183253
H	6.70604121277746	3.60364788229051	14.29077523513968
H	8.30992551384770	3.75795564800162	15.03894627146891
H	7.87391556284337	4.78321404550992	13.67147258149722
C	8.03811093533338	6.39909099576435	15.93492290969688
H	8.42284192317421	6.90191790075376	15.04246973956914
H	8.87829918527088	5.89682395584496	16.42069981764903
H	7.65286012621327	7.16519195191098	16.61571351721424

Transition state 1

Cl	5.10684632816091	6.57686755287059	25.92005002640041
Cl	10.04110494311388	3.70009397537074	18.37159757920637
O	2.48877370188907	5.90436928479437	24.93044763229415
H	1.61611560878332	5.71437682843831	24.55035530040148
O	3.73463791738363	5.71830284519972	20.30636584131564
O	4.38529850394563	3.09038248310476	19.97186186454648
O	4.40749906591352	4.18313187450982	18.05328697066989
O	2.91457214138672	5.99725865587532	16.76348950373345
O	2.53342015518849	7.79023103833765	14.96642676145645
H	2.35180111184539	7.16217248956422	15.71803954793920
O	6.09122899769877	8.97962796123133	12.15316118469690
H	7.02313206967611	8.77158255195473	11.97944881015775
O	10.16681001045712	4.51919714040658	21.13596230502756
H	10.17050731672098	4.80980766099915	22.06190081455948
C	5.76277242687457	6.09445352051109	23.27890724527631
C	4.75134696106500	6.14796847875744	24.25041870576702
C	3.41173450542906	5.89349946446322	23.93720269204379
C	3.07321160372050	5.72803448730323	22.59937368202538
H	2.02707444674251	5.66559507557518	22.32509045549848

C	4.06461429328410	5.66828174613567	21.61766898738952
C	5.45378881440706	5.62332329933755	21.95178884983947
C	6.49237201245109	5.08332211464998	21.00845743071684
C	6.26569895662413	4.52170000164517	19.70520864560233
C	4.94952798074494	3.91618217843859	19.29815463720928
C	5.00936731670086	4.94602900945956	17.07275698597454
C	4.10244883188793	5.88704758882514	16.39241484844420
C	4.65063110507430	6.65830414707376	15.28697974361949
C	3.81287605850913	7.60751648666472	14.61601617255293
C	4.31647583841173	8.37653012553591	13.56751578912021
H	3.67707378690494	9.09345098819218	13.06571769881476
C	5.64212382888840	8.20913808636471	13.17972840848277
C	6.47642126224624	7.27951865031115	13.82403236107020
H	7.50462746274647	7.17902371250467	13.48607306584666
C	5.99561105668723	6.50142486261334	14.87359883373760
C	6.93325711535297	5.48558185121604	15.54152520211906
C	6.27692337681029	4.72457214044550	16.68279961028110
C	7.04758556420006	3.66714026454562	17.45370638225657
H	6.44178860505161	2.75029870311848	17.48863217609636
H	7.97539910139335	3.40520043773853	16.95088144558882
C	7.34553581407993	4.13954455807748	18.86894642833140
C	8.66049215642140	4.19046865309336	19.33940193547473
C	8.89496088573332	4.57588536021516	20.65996787895447
C	7.83153946688999	5.01082253677428	21.44368365563556
H	8.08279731062987	5.24797859682463	22.46216521531062
C	7.08856175497494	6.72060068949203	23.68683048141576
H	7.75436902780146	6.04048387244993	24.23539883422289
H	6.88629693399463	7.55279549869404	24.36518724947533
H	7.62219197349857	7.12358166016886	22.82495309742689
C	2.35736813043182	5.60879583000606	19.92457999481560
H	2.35861974586523	5.62421679323854	18.83673796600767

H	1.78703102553275	6.46052762007251	20.31424424377269
H	1.93275140080515	4.66647449411789	20.28735126933587
C	7.38028986882174	4.45343203636221	14.46421649809507
H	6.51506672945826	3.92465902534742	14.05104812131244
H	8.07172657042917	3.71433091009360	14.88029449726989
H	7.89459335350249	4.95773478946009	13.64092335984694
C	8.17693472864014	6.23572515546748	16.09935061038308
H	8.70419487201238	6.75858617429774	15.29570388665350
H	8.88470097124927	5.54343362043272	16.56331866445995
H	7.87614709485472	6.97648936122950	16.84710391401838

Transition state 2

Cl	0.98476612325313	7.60591371512670	22.63484643382823
Cl	8.97029028731224	2.99077382107376	18.85741476032540
O	1.37425266607200	5.96895138170831	24.95633688449109
H	1.50840038879303	5.36845689673405	25.70668453779279
O	4.75602434763583	3.12164628609771	23.10628219387501
O	5.24500176206535	7.39778298710376	19.20247417918429
O	4.52549552606910	5.97562386100906	17.58710296938353
O	4.02650846835265	8.11070031868739	15.90842224372569
O	4.74030042680906	9.73803627593484	14.04415546996863
H	4.19368564599121	9.30935970147336	14.76033753187950
O	8.89761888122199	9.13215922721032	11.95205876655206
H	9.67450130073065	8.55086820498183	11.96807251555388
O	8.68076467204852	3.38091095096917	21.68651658740435
H	8.51923534205478	3.47638265839045	22.63874932117133
C	3.07327423532943	6.05172308768229	21.69360796551651
C	2.19813275007748	6.33924339401392	22.75998076493966
C	2.22740524112124	5.62626225653415	23.95842419773836
C	3.09316972311410	4.54077047003136	24.05460272626653
H	3.05137029791270	3.91344695869348	24.93810112489133
C	3.99872260515281	4.26187817149637	23.03352691093252

C	4.13733361162374	5.10864294825188	21.87758739263523
C	5.36176518236882	4.89444750387764	21.01137258586496
C	5.53431919994408	5.08065274366935	19.60043044681571
C	5.04799050992615	6.26420149217524	18.84432719861983
C	5.42941427347867	6.34384013431410	16.59251764463052
C	5.10416330544534	7.50663657367707	15.75784780688865
C	6.10816951096590	7.88109001341158	14.76805601179571
C	5.86864401846872	9.02494336243478	13.94243855528481
C	6.81759494874116	9.42869150763969	13.00282586036108
H	6.63182323128235	10.29733034713339	12.38184819795258
C	7.99832154556051	8.70353369566452	12.87968349095949
C	8.24704648181874	7.57474422477508	13.67933729846775
H	9.18151622833698	7.03579559805737	13.54425804910943
C	7.31486554685997	7.15285910218286	14.62452813433723
C	7.62082320870834	5.89744748174224	15.46210515904391
C	6.53137728225554	5.58885454708262	16.47670637614397
C	6.60319346326003	4.33769379808385	17.35100658562758
H	5.72297374784120	3.73538007963454	17.08409453476362
H	7.47224883335100	3.74854670478974	17.07061839289196
C	6.59293914863389	4.43917390368186	18.88525868072322
C	7.61457988177244	3.81713253189170	19.61336029001310
C	7.60740700760833	3.87169273570606	21.01181400693385
C	6.48966027245028	4.37199963996754	21.66918395536520
H	6.49871005775560	4.31879147802690	22.74933779340114
C	2.72271278183360	6.68449287833736	20.36855953789820
H	1.64860063355449	6.87225472677806	20.33289627090409
H	2.94657661600609	6.00860842520596	19.54225935244679
H	3.23137275923023	7.63348018966405	20.18469141427682
C	4.41720803462498	2.11478245504324	24.04779637319143
H	5.04162490505311	1.25577814642536	23.79333367190397
H	3.35925914363791	1.83335997030457	23.97208557577010

H	4.63851219837637	2.42356948069184	25.07866155308099
C	7.76260505397233	4.68652018826698	14.49411995139454
H	6.83264867105441	4.52364557533707	13.93932971457858
H	8.00977481562014	3.76711814603547	15.03447634554877
H	8.56180678544299	4.86694015807829	13.76920753306379
C	8.95042325001329	6.10973860259358	16.24097062480445
H	9.78107032365590	6.28401762109548	15.55015436838322
H	9.19894977550248	5.23190647761363	16.84555031220860
H	8.87372106284654	6.97587918570509	16.90612786649423

Formicapyridine A (13)

Lowest energy state

O	0.71844448145027	6.47659853167948	22.08091997671434
H	0.03885574605365	7.07313972158046	21.73035067018867
O	0.28223801709344	4.55597443243130	17.67015744169877
H	-0.35399303450340	5.28277419025584	17.58665795348237
O	3.34887917787874	5.11747871755674	17.42698913381565
H	3.77218158111913	5.90740123481374	16.97604016799200
O	4.45662403549503	6.55921372012092	15.71335426638708
O	5.54936738693191	8.12755302823046	14.04091809117922
H	5.13328734549266	7.80458284930427	14.88414843751958
O	6.74767786858893	6.49412536217430	9.82151108912485
H	6.73182447679327	5.68260922759038	9.28947260904227
N	1.77924117980691	1.37675320230455	18.55102786441811
C	2.42465813219489	3.66457477827597	20.49235333309850
C	2.03670635927233	4.62600442008299	21.42923768876999
H	2.49211478168453	4.65620527602709	22.41401930621216
C	1.05510358320775	5.56751938503584	21.11521453034333
C	0.45310525043524	5.55885417230160	19.85554602627472
H	-0.32075513747144	6.28492501168809	19.60525024536671
C	0.85008616618554	4.60477314373436	18.91572750801251
C	1.83567874459253	3.65165349809997	19.21234895698728

C	2.17038277180222	2.59568013641329	18.20401629503412
C	2.82751584965474	2.85492359760328	16.94984442577733
C	3.37994608524912	4.11824272391170	16.55269533483263
C	3.96613604681972	4.28533200069082	15.26701830653512
C	4.49970053141366	5.58644192605279	14.88973432097707
C	5.08006316238556	5.78760073477224	13.57193246408124
C	5.58479343494099	7.08104644190884	13.20584085277522
C	6.13861454674872	7.29570534254785	11.94366773642413
H	6.51466121129137	8.27746630685716	11.68059708291675
C	6.20011877089828	6.24145876528970	11.04042847924673
C	5.71416821036734	4.96830441241056	11.38197313503815
H	5.78429352233208	4.16999642094125	10.64783123222110
C	5.15560164434598	4.72900867120376	12.63387277181092
C	4.64735487140300	3.31789109389515	12.95602483963852
C	4.03684655452049	3.18120117448673	14.35880157201998
C	3.55185269403984	1.95974933390675	14.76180715911881
H	3.61040466333760	1.09646759516903	14.10625234470684
C	2.95943375861502	1.76627415085816	16.03816219452776
C	2.49802041944532	0.49244681503132	16.44475113046665
H	2.59244073666536	-0.35162524237546	15.76629689570399
C	1.95087200934499	0.32995576671767	17.69897961456890
C	3.49070492895013	2.65879545678452	20.86559644544501
H	3.08494192435484	1.64179671037663	20.86302794794197
H	3.89482250721802	2.87116593586672	21.86003692040240
H	4.32305019081097	2.67455736647530	20.15104364797740
C	1.48359364233032	-1.00780895303379	18.21156184091744
H	2.03300478555080	-1.27965174421035	19.12112316291441
H	1.62327124109740	-1.79973236831966	17.46920334361573
H	0.42291420150817	-0.96096113040590	18.48445018499779
C	5.84634884077061	2.33269194620777	12.84446317732130
H	6.62408304293463	2.58625161295088	13.57244684697279

H	6.29234266666930	2.36701540156155	11.84560633055210
H	5.52580586939049	1.30415607329744	13.03427256671615
C	3.55635712388309	2.93779674044056	11.91384876833603
H	3.18887361757190	1.92250753181648	12.08837505720620
H	3.95112789990613	2.98150101447820	10.89407759272814
H	2.70501987912967	3.62335633413244	11.97898268090404

Transition state 1

O	2.08184892193880	4.93778534059186	23.33989418718066
H	1.79957055344426	4.23145355564950	23.94227150914719
O	1.25314660619989	1.49041934418027	20.15884860763429
H	1.33572140701723	1.03407941655959	21.01195119423120
O	1.92998353641410	5.59061342587050	16.17824628071106
H	2.35921185355621	6.30536013584471	15.61753150089603
O	3.48717970999962	6.75655568195252	14.58673270508511
O	5.11805558931299	8.02865324825495	13.10381517148165
H	4.37364842104961	7.85414122676185	13.74005008859633
O	8.24918782855515	5.64079847161353	10.56840228057018
H	8.53564239935554	4.73353285152376	10.37701062007599
N	0.55248750052280	2.22230299695496	17.92756139124618
C	2.27316988010978	5.00179864754884	19.68504219656715
C	2.36042090294486	5.36267241177809	21.03298408969636
H	2.73497378136885	6.34335402039667	21.30545514122774
C	1.99309181597708	4.49380736226460	22.05457543355385
C	1.59470108479756	3.20736183513895	21.72014362612062
H	1.36227461553529	2.48178738716761	22.50018437530415
C	1.50991202687540	2.81542478367887	20.37987106544873
C	1.74968013745949	3.72734205373226	19.29125539990585
C	1.47408876635740	3.17197689662196	17.91107609933176
C	2.24176429901751	3.30016291056305	16.66608155978031
C	2.60466938950610	4.46735189281866	15.93442012443119
C	3.57063661668847	4.41105960864614	14.88922076332281

C	4.01686687471032	5.64023267553136	14.26381909775008
C	5.09520868849428	5.60542836467390	13.28731651597221
C	5.61340070765531	6.83313266248872	12.75653933627975
C	6.67313190663323	6.82242895589723	11.84901429279899
H	7.05843049005915	7.75751079063662	11.45942933637184
C	7.21829075540103	5.60609410677631	11.45590518054237
C	6.71521961487656	4.39305197701648	11.95515832540898
H	7.15906047325391	3.46330460526772	11.60893031031281
C	5.66215912847329	4.37736192207099	12.86513109175059
C	5.11342702561968	3.02341042463556	13.33779281048828
C	4.03392581997407	3.14277177579816	14.42141809162115
C	3.42026023972771	2.01391872013221	14.91022071734961
H	3.62648940782477	1.03776476567326	14.48208075070489
C	2.45294809725237	2.07022956620239	15.95497073903600
C	1.60455310106297	0.96991266136496	16.22278885866277
H	1.72758552897396	0.04007953091693	15.67467914779648
C	0.53201031829164	1.18308844660131	17.06841825326181
C	2.80770423154553	6.05809075804441	18.74796618335506
H	3.55111968713814	5.64856348267777	18.05915304291009
H	3.29657122851676	6.84485238032308	19.32910453037405
H	2.02100375275692	6.51134057738216	18.14595399831446
C	-0.64991532454902	0.25614822311302	17.16535139547518
H	-0.77251901230319	-0.09667035324223	18.19544844798264
H	-0.54130327210712	-0.60522694948634	16.49903945861478
H	-1.57166194121053	0.78694734466347	16.89538496668495
C	6.27585593943797	2.16972819313829	13.91712733327176
H	6.73072814878034	2.66773988139048	14.77989221276557
H	7.05846322022725	1.99839998801394	13.17129200155411
H	5.90760893544828	1.19318631460007	14.24539833335064
C	4.50418688312759	2.30551765744797	12.09868676291146
H	4.12495405891418	1.31457140283219	12.36612928502112

H	5.25560430216800	2.17896496028240	11.31263226667248
H	3.67437133982060	2.88805468102143	11.68509251308628

Transition state 2

O	0.72267520863174	7.05459166730849	22.03385654675187
H	1.49330763491121	7.56835929108382	22.32302636624951
O	4.04387852372504	5.02062515893440	19.26872718591604
H	4.55653159722150	5.70159381413835	19.73111298499483
O	5.12067679422591	3.29545490045899	17.85574993953987
H	5.99330919449111	3.65708678060451	17.52201875742340
O	6.87539082724219	4.25642355204611	16.32688517689315
O	8.66006490158826	5.31792199969458	14.86064346902122
H	8.19827421456117	4.96961734721640	15.67061612644379
O	7.95676626754643	5.60529405819603	10.23837083666617
H	7.30304550480144	5.38320130938264	9.55638232951688
N	1.27701038520094	1.94409583484276	18.45637333211612
C	0.46716016890961	4.31667659180835	19.62446609878505
C	0.14554019949311	5.30113770591337	20.55876180537435
H	-0.88641884066816	5.44935546086171	20.85928555264651
C	1.11807933220327	6.13396955313094	21.10950650331387
C	2.42737255134710	6.01633579022261	20.66658869745498
H	3.20256857014056	6.68635020739783	21.03920979119728
C	2.76579050286579	5.04543357976629	19.71490570220107
C	1.82980307575396	4.10735142287155	19.20863501346009
C	2.11262978823260	2.95609089121470	18.27000351134928
C	2.92421686145217	2.93477944057802	17.04721515040091
C	4.28828373647222	3.30906997452140	16.82598014299134
C	4.77031562108912	3.55146208889241	15.50450227361286
C	6.10749098510612	4.08014914904865	15.32187037546736
C	6.56869160476408	4.44062080747798	13.98841758359717
C	7.84732776229428	5.06944021327671	13.82441014933022
C	8.29146902538076	5.45543155803335	12.55934868509303

H	9.25736173197265	5.93474099676253	12.45018272283353
C	7.48469341821439	5.21466340325241	11.45361929005004
C	6.23579040345518	4.58723686395400	11.59320302856520
H	5.64304581074398	4.40234990979274	10.70107632063934
C	5.76803564805968	4.19856292132754	12.84547277697345
C	4.41598954881151	3.47756530057986	12.93838012946776
C	3.97100836073133	3.18851819256803	14.37783148166027
C	2.80769902824233	2.49389661095392	14.61216409515072
H	2.23714647293131	2.06690572344201	13.79324582611505
C	2.30839370214307	2.28125927556240	15.92864122253223
C	1.26727794060277	1.34966281420858	16.16814475249749
H	0.83224128737530	0.79857951115456	15.33895651502248
C	0.93521314394590	1.07312083832834	17.47742008825119
C	-0.74106530914207	3.58422644225502	19.06412348238844
H	-0.66205877905705	3.41266749267821	17.98804176777421
H	-1.63780098194674	4.18781921357307	19.24243361996583
H	-0.88453966249328	2.60954275967704	19.53778722574516
C	0.10919264719904	-0.11541370249699	17.89221614116391
H	0.67585431321472	-0.74265254939882	18.59169476955283
H	-0.17229350504802	-0.72853251819518	17.03059035564246
H	-0.80137475213020	0.20155508950174	18.41420463518518
C	4.54054655261040	2.13048078820346	12.16976396801912
H	5.29814024752432	1.48991714262131	12.63313890500089
H	4.82963288373627	2.30012570179346	11.12769202130009
H	3.58965076626194	1.58941775829562	12.17139605837763
C	3.32088703741468	4.35878159257595	12.27353113644606
H	2.34956936885542	3.85626661050194	12.30999177790335
H	3.55463938910998	4.56304171120718	11.22384594087123
H	3.22668228767760	5.31749795639706	12.79423185709637

12. REFERENCES

(1) Hu, G.; Doerksen, R. S.; Ambler, B. R.; Krische, M. J. Total Synthesis of the Phenylnaphthacenoid Type II Polyketide Antibiotic Formicamycin H via Regioselective Ruthenium-Catalyzed Hydrogen Auto-Transfer [4 + 2] Cycloaddition. *J. Am. Chem. Soc.* **2024**, *146* (38), 26351–26359. https://doi.org/10.1021/jacs.4c09068.

(2) Fukumoto, A.; Kim, Y. P.; Iwatsuki, M.; Hirose, T.; Sunazuka, T.; Hanaki, H.; Omura, S.; Shiomi, K. Naphthacemycins, Novel Circumventors of β-Lactam Resistance in MRSA, Produced by Streptomyces Sp. KB-3346-5. II. Structure Elucidation. *J. Antibiot. (Tokyo).* **2017**, *70* (5), 568–573. https://doi.org/10.1038/ja.2017.29.

(3) Huo, C.; Zheng, Z.; Xu, Y.; Ding, Y.; Zheng, H.; Mu, Y.; Niu, Y.; Gao, J.; Lu, X. Naphthacemycins from a Streptomyces Sp. as Protein-Tyrosine Phosphatase Inhibitors. *J. Nat. Prod.* **2020**, *83* (5), 1394–1399. https://doi.org/10.1021/acs.jnatprod.9b00417.

(4) Yuan, J.; Wang, L.; Ren, J.; Huang, J.-P.; Yu, M.; Tang, J.; Yan, Y.; Yang, J.; Huang, S.-X. Antibacterial Pentacyclic Polyketides from a Soil-Derived Streptomyces. *J. Nat. Prod.* **2020**, *83* (6), 1919–1924. https://doi.org/10.1021/acs.jnatprod.0c00161.

(5) Qin, Z.; Munnoch, J. T.; Devine, R.; Holmes, N. A.; Seipke, R. F.; Wilkinson, K. A.; Wilkinson, B.; Hutchings, M. I. Formicamycins, Antibacterial Polyketides Produced by Streptomyces Formicae Isolated from African Tetraponera Plant-Ants. *Chem. Sci.* **2017**, *8* (4), 3218–3227. https://doi.org/10.1039/C6SC04265A.
